# Targeting TWEAK to enhance mitochondrial biogenesis and attenuate age-related muscle mass loss

**DOI:** 10.1038/s41598-026-64401-2

**Published:** 2026-08-14

**Authors:** Zhuoya Maimaitiwusiman, Saiyare Xuekelati, Anyan Wang, Nadila Airiken, Yanbin Xu, Shuke Guo, Yining Yang, Hongmei Wang

**Affiliations:** 1 Xinjiang Medical University, Urumqi, 830001 Xinjiang Uygur Autonomous Region China; 2 Comprehensive Health Internal Medicine Ward ll of People’s Hospital of Xinjiang Uygur Autonomous Region, Urumqi, 830002 China; 3 Xinjiang Key Laboratory of Cardiovascular Homeostasis and Regeneration Research, Urumqi, 830001 China; 4 Department of Cardiology, People’s Hospital of Xinjiang Uyghur Autonomous Region, Urumqi, 830001 China

**Keywords:** Gastrocnemius, Atrophy, TWEAK, Fn14, Mitochondria, Cell biology, Diseases, Molecular biology, Physiology

## Abstract

Mitochondrial dysfunction is an important cause of sarcopenia, and TWEAK/Fn14, as one of the major muscle wasting cytokines, its role in the development of sarcopenia by regulating mitochondrial biogenesis remains unclear. Expression of TWEAK in old and young mice was both detected. TWEAK was silenced in C2C12 myocytes using lentiviral vectors. Immunofluorescence, western blot, real-time polymerase chain reaction (RT-PCR), and ELISA were enrolled to analyze the effects of TWEAK on myotube size, mitochondrial content, mitochondrial ROS, and inflammatory factors. Additionally, aged mice received two injections of AAV9 vectors at 15 and 17 months of age. Upon reaching 18 months of age, the effects of TWEAK knockdown on grip strength, muscle mass, and gastrocnemius muscle indices were evaluated. TWEAK/Fn14 expression was significantly increased in old mice (*p* < 0.001). Compared with young controls, old mice exhibited a significant decrease in grip strength (*p* < 0.001) and a significant increase in lean mass (*p* < 0.05), whereas no significant difference was observed in fat content. In DEX-treated C2C12 myotubes, TWEAK knockdown significantly increased myotube diameter, enhanced ATP content and mitochondrial quantity, upregulated protein expression of SIRT1, PGC-1α, and p-AMPK, and inhibited mitochondrial ROS, Ca^2+^ levels, p-p38 expression, and the secretion of inflammatory cytokines (TNF-α, IL-1β, IL-6, and iNOS). In aged mice, TWEAK knockdown did not significantly alter forelimb grip strength or lean mass, but significantly increased fat mass (*p* < 0.05). Mechanistically, TWEAK knockdown promoted AMPK signaling, inhibited p38 MAPK activation, enhanced mitochondrial biogenesis, and reduced serum levels of IL-1β, IL-6, and iNOS (*p* < 0.05), whereas serum TNF-α levels showed no significant difference. TWEAK knockdown attenuates age-related skeletal muscle mass loss and improves mitochondrial biogenesis in skeletal muscle, accompanied by modulated inflammatory factor release and altered AMPK-p38 MAPK signaling activity. However, no significant improvement in forelimb grip strength was observed in the in vivo experiment. These findings indicate that TWEAK suppression may represent a promising strategy for preserving muscle mass and metabolic homeostasis during aging, though its capacity to fully restore functional capacity requires further investigation.

**Supplementary Information:**

The online version contains supplementary material available at https://doi.org/10.1038/s41598-026-64401-2.

## Introduction

Sarcopenia, characterized by a significant decline in muscle mass, strength, and physical function, affects the quality of daily life in older adults and may lead to serious health issues such as fractures, frailty, and increased mortality^1,2^. The incidence of sarcopenia in elderly people remains high, especially among female^3,4^. Notably, as the global aging trend intensifies, strategies targeted at reducing the high incidence of sarcopenia and alleviate obstacles in daily activities for elderly people is urgently needed.

TNF-like weak inducer of apoptosis (TWEAK) and its receptor Fibroblast growth factor-inducible 14 (Fn14) emerged as key regulators of muscle mass and function. Increased TWEAK/Fn14 levels were observed in muscle atrophy, indicating that this receptor may mediate the effects of catabolic stimuli on muscle tissue^5,6^. Moreover, the interaction between TWEAK and Fn14 can activate downstream signaling pathways to promote mitochondrial biogenesis. It is well known that mitochondria play a crucial role in maintaining skeletal muscle health, providing the necessary energy for cells and regulating key cellular functions^7^. In sarcopenia, the activation of TWEAK signaling may suppress the expression of PGC-1α and other related factors, thereby reducing mitochondrial biogenesis and muscle function^8^. Peroxisome proliferator-activated receptor gamma coactivator 1-alpha (PGC-1α) is an important transcriptional coactivator regulating mitochondrial biogenesis and oxidative metabolism^9^. The up-regulated PGC-1α expression could improve muscle fatigue by increasing muscle endurance and oxidative capacity^10^. Previous evidence showed, sirtuin 1 (SIRT1), as an NAD+-dependent deacetylase, could promote the activity of PGC-1α and further enhance mitochondrial function and muscle endurance. Both PGC-1α and SIRT1 are important in improving skeletal muscle mitochondrial function and endurance^11,12^.

Additionally, in sarcopenia, the interaction between inflammation and the TWEAK/Fn14 signaling pathway^13^, typically characterized by elevated pro-inflammatory cytokines, has been proven to exacerbate muscle loss and impair muscle regeneration^14^. In muscle regeneration, TWEAK/Fn14 signaling affects myoblast fusion and differentiation^6^ via targeting TWEAK and AMPK influences energy homeostasis^15^. Activation of AMPK has been shown to enhance mitochondrial biogenesis and improve mitochondrial function^16^. In skeletal muscle, the p38 MAPK pathway is involved in the regulation of muscle cell growth and differentiation^17^. Our previous research found low DNA methylation in the promoter region of TWEAK/Fn14 in sarcopenia patients, with elevated gene and protein levels in peripheral blood^18^. However, the specific role of TWEAK in sarcopenia remains unclear due to the complexity and interrelationship of these molecular pathways.

Thus, this study was designed to explore the role of TWEAK in the progression of sarcopenia and its underlying mechanisms. Using C2C12 myotube cells and naturally aging mouse models, we observed a significant increase in the expression levels of TWEAK and its receptor Fn14. Moreover, immunofluorescence, western blot, RT-PCR, and ELISA were enrolled to analyze the effects of TWEAK on myotube size, mitochondrial content, mitochondrial ROS, and inflammatory factors. Meanwhile, aged mice received two administrations of AAV9 vectors at 15 and 17 months of age (with a 2-month interval), and were maintained until 18 months of age to examine the impacts of TWEAK knockdown on forelimb grip strength, lean muscle mass, and molecular biomarkers in the gastrocnemius muscle. Finally, our data suggest that TWEAK might be a valuable therapeutic target for attenuating age-related muscle mass loss and preserving mitochondrial homeostasis, with its effects correlated with changes in mitochondrial biogenesis, inflammatory cytokine release, and AMPK-p38 MAPK signaling.

## Methods

### Animal

Male C57BL/6J mice at 4 months (*n* = 8) and 15 months (*n* = 24) were purchased from the Animal Center of Xinjiang Medical University and Finoc Bio-tech Co., Ltd., respectively. Three to four mice were housed per cage with free access to standard food and water under conditions of 50–60% relative humidity, 24 ± 1 °C temperature, and a 12-hour light/dark cycle. A total of 24, 15-month-old mice were randomly sub-grouped into three groups: (1) Control group: multiple injections of 25 µL PBS into both gastrocnemius muscles (Control, *n* = 8); (2) AAV9-sh-Ctrl group: 2.5 × 10^11 gc of sh-Ctrl diluted with PBS to 25 µL and injected into multiple sites of the bilateral gastrocnemius muscles (AAV9-Ctrl, *n* = 8); (3) AAV9-sh-TWEAK group: similar injection scheme as the sh-Ctrl group, but injecting sh-TWEAK (AAV9-TWEAK, *n* = 8). The first AAV9 vector injection was administered when mice reached 15 months of age. Following a 2-month interval, a booster injection was performed at 17 months of age. After completion of both injections, all mice were maintained under standard housing conditions until 18 months of age. Behavioral assessments and body composition analyses were conducted at 18 months, after which mice were immediately euthanized for tissue collection. This two-injection regimen, spaced 2 months apart, ensured sustained knockdown of TWEAK expression throughout the entire experimental period. All experiments were approved by the ethics boards of People’s Hospital of Xinjiang Uygur Autonomous Region (Approval Number: KY2021031726). All experiments were performed in accordance with relevant guidelines and regulations, and complied with the ARRIVE guidelines.

Prior to experimentation, a subgroup allocation scheme was pre-established according to the distinct tissue-processing requirements of each assay, rather than being determined post hoc. Within each experimental group of aged mice (*n* = 8 per group), six mice were randomly selected for behavioral grip strength testing, dual-energy X-ray absorptiometry (DEXA) body composition analysis, serum ELISA, and quantitative RT-PCR (qRT-PCR) analysis of gastrocnemius muscle; these assays could be performed on the same animals or conducted sequentially. An additional three mice were randomly assigned to Western blotting and immunofluorescence staining, as these procedures are tissue destructive and preclude the use of the same muscle specimen for the aforementioned analyses. The remaining two mice served as backup samples to account for unexpected events such as intraoperative mortality; no animal deaths occurred during the actual experiment, and the backup samples remained unused. Additionally, a young control group of eight mice was included for baseline comparison, with data derived from an independent cohort conducted in parallel. Investigators performing all behavioral assessments, body composition analyses, and subsequent molecular biological assays were blinded to group allocation. Group codes were maintained by an independent researcher and were only revealed after completion of data analysis.

Behavioral tests were conducted 1 month after AAV9 injection. Grip strength was detected by a mouse grip strength meter. A live body composition analyzer was applied to measure body composition. Finally, all mice were euthanized by cervical dislocation.

The sh-TWEAK encoding AAV9 vector (AAV9-U6-sh-TWEAK-CAG-EGFP) and the AAV9 control vector (AAV9-U6-sh-Ctrl-CAG-EGFP) were constructed by Gikai Gene Company (Shanghai, China).

### Animal experiment

#### Grip strength test

Grip strength was measured using a force meter (Huabei, China) to assess the forelimb grip strength of experimental and control mice. The mouse’s tail was held while its forelimbs gripped the metal mesh of the force meter, and the mouse was gently pulled backward. Then, the reading on the force meter was recorded. This procedure was repeated five times, and the average of the five valid readings was taken as the mouse’s grip strength^19^.

#### Dual-energy X-ray absorptiometry (DEXA) testing

After mice were anesthetized with the prepared mixed anesthetic solution. The anesthetic solution was prepared as follows: 250 mg Zoletil 50 was mixed with 2.5 mL xylazine hydrochloride (100 mg/mL), and diluted with 22.5 mL sterile normal saline to obtain a mixture of Zoletil 50 (10 mg/mL) and xylazine hydrochloride (10 mg/mL) for subsequent use. After complete anesthesia was achieved, DEXA was used to analyze body composition, including fat, water, and lean tissue content based on specific signal processing and algorithms. Thereafter, muscle and fat tissue contents in mice were further analyzed^20^.

### Cell experiment

#### Cell treatment

Mouse C2C12 myoblast cell line (purchased from Puno Bio, Wuhan, China) was cultured at 37 °C in a 5% CO2 atmosphere with saturated humidity in DMEM (high glucose) medium, and the medium was supplemented with 10% FBS, and 1% penicillin/streptomycin. When C2C12 cells reached 70% confluence, the growth medium was discarded. After washing by pre-warmed PBS twice, cells were suspended by differentiation medium (DMEM + 2% horse serum) and cultured for further 5 days, with the medium changed every 24 h to induce myotube formation^20^. A muscle atrophy established by treating with 5 µM dexamethasone (DEX) for 24 h^21^. It should be noted that dexamethasone-induced C2C12 myotube atrophy primarily mediates muscle atrophy through the glucocorticoid receptor pathway, which differs from the pathophysiological mechanisms of natural aging-related sarcopenia; these two models were used in a complementary manner to validate the biological function of TWEAK. All cell experiments were repeated at least three times.

#### Myotube size

Myotube size was assessed by measuring the diameter of the myotubes. Digital images of myotube cultures were captured under a microscope at 100x magnification. ImageJ software (National Instruments) was used to measure the average diameter of myotubes in *n* = 5 randomly selected fields, with n myotubes measured in each field^22^.

#### ATP measurement

ATP levels in the gastrocnemius and C2C12 cells were measured using an ATP detection kit (A095-1-1, Nanjing Jiancheng) according to the manufacturer’s instructions.

#### ROS measurement

ROS detection kit (E004-1-1, Nanjing Jiancheng) was used to measure the total ROS generated in C2C12 cells and gastrocnemius muscle. Briefly, treated cells and gastrocnemius muscle samples were incubated with DCF-DA as recommended by the manufacturer. The fluorescence values were then measured using a fluorescence microplate reader with an excitation wavelength of 500 nm and an emission wavelength of 525 nm.

#### Fluo-4AM measurement of calcium ion concentration

According to the manufacturer’s guidelines, Fluo-4AM dye (F14201, Thermo Fisher) was used to load the fluorescence probe by incubating C2C12 cells and gastrocnemius tissue for 30 min. Subsequently, calcium ion concentration changes were assessed by detecting the fluorescence of Fluo-4 using a flow cytometer (Hangzhou Powclin Medical Technology Co., Ltd).

#### Immunostaining and confocal laser scanning microscopy

Mitochondrial quantity and mitochondrial ROS (MitoSOX Red) levels in cells were detected using MitoTracker (M7512, Thermo Fisher) and MitoSOX Red (M36008, Thermo Fisher) kits. First, 500 µL of 100 nM MitoTracker (red) and 500 µL of 5 µM MitoSOX (red) were added and incubated at 37 °C for 30 min. After fixing with 4% paraformaldehyde for 20 min, 500 µL of 1 µg/mL DAPI was added for staining, followed by observation of red fluorescence under a laser scanning confocal microscope. Finally, ImageJ software was used to count the number of mitochondria and analyze the mitochondrial ROS levels in each cell.

### ELISA

Mouse ELISA kits were applied to measure the levels of IL-1β (70-EK201B/3, Multi Science, China), TNF-α (70-EK282/4), IL-6 (70-EK206/3), and iNOS (CSB-E08326m, CUSABIO, China) in serum and cell supernatants, following the manufacturer’s instructions.

### Real-time quantitative PCR

Total RNA was extracted from gastrocnemius tissue or C2C12 myotubes using TRIzol reagent (15596026, Ambion). Subsequently, RNA was reverse transcribed into cDNA using a reverse transcription kit (G492, abm). Real-time quantitative PCR was performed using the EvaGreen Express 2×qPCR MasterMix-Low Rox kit (G891). Relative gene expression was calculated using the 2^−ΔΔCT^ method with β-actin as the reference gene. PCR primers are listed in Table 1.

**Table 1:** The sequence of primers used in the study.

Primer	Primer sequences (5’-3’)	
	Forward	Forward
TWEAK	TGAGGGAAAGGCTGTCTACC	AGCCTTAAGATGAGCCCAGG
Fn14	TCCTCGTGTTGGGATTCGG	TAGAAACCAGCGCCAAAACC
Mouse actin	GGCTGTATTCCCCTCCATCG	CCAGTTGGTAACAATGCCATGT

### Western blot

C2C12 myotubes and gastrocnemius muscle tissue samples were lysed with RIPA buffer (AR0105, Boster) supplemented with a protease inhibitor cocktail (AR1178, Boster) to extract total protein. The protein concentration was determined using a BCA protein assay kit (DQ111-01, Comin). Equal amounts of protein were separated by SDS-PAGE (A100227, Sangon Biotech) and subsequently transferred onto polyvinylidene fluoride (PVDF) membranes (IPVH00010, Millipore). Following transfer, membranes were blocked at room temperature for 1.5 h in TBST (Tris-buffered saline containing 0.1% Tween-20) supplemented with 5% non-fat milk to prevent non-specific binding. Primary antibodies were diluted in the same blocking buffer according to the ratios indicated in Table 2. The membranes were incubated with the diluted primary antibodies at 4 °C overnight. The following day, the membranes were washed three times with TBST for 10 min each at room temperature. Thereafter, they were incubated with corresponding horseradish peroxidase (HRP)-conjugated secondary antibodies (diluted in blocking buffer as specified in Table 2) for 1 h at room temperature. After incubation, the membranes were washed again three times with TBST, each for 10 min. Finally, protein bands were visualized using an ECL chemiluminescence kit (AR1171, Boster Biological Technology) and imaged with a chemiluminescence imaging analysis system (Chemiscope 3000, Shanghai Qinxiang) for quantification.

**Table 2:** Antibody dilution ratio in western blot.

Primary antibody	Dilution ratio	Secondary antibodies	Dilution ratio
β-actin	1:1000	Goat anti mouse IgG H&L (HRP)	1:10000
SIRT1	1:1000		1:5000
TWEAK	1:800	Goat anti rabbit IgG H&L (HRP)	1:5000
Fn14	1:800		1:5000
PGC1-α	1:1000		1:5000
AMPK	1:800		1:5000
p-AMPK	1:800		1:5000
p38	1:1000		1:5000
p-p38	1:800		1:5000

### Statistical analysis

Data are presented as mean ± standard deviation or median with interquartile range. Statistical analysis was performed using SPSS 19.0 software (IBM, USA). For normally distributed data, Student’s t-test (two-tailed) was applied to calculate differences between two groups, and one-way analysis of variance (ANOVA) was performed among more than two groups, followed by Dunnett’s post-hoc multiple comparisons. For non-normally distributed data, Mann-Whitney test was enrolled to detect differences between two groups, and Kruskal-Wallis test was applied among more than two groups, followed by Dunn’s post-hoc multiple comparisons. *P* < 0.05 was considered statistically significant.

## Results

### Upregulation of TWEAK expression in the sarcopenia mouse model

**Fig. 1:**
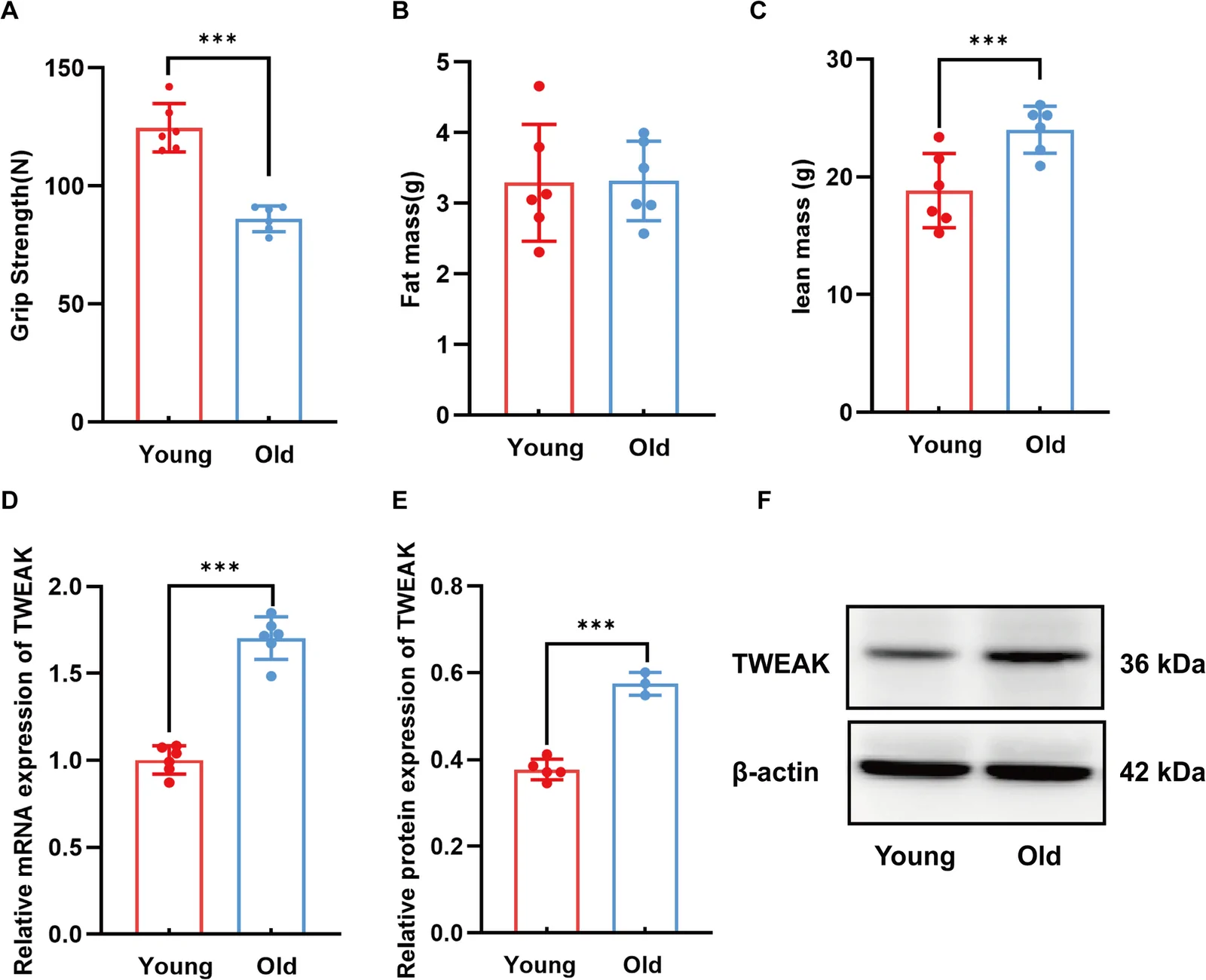
Upregulation of TWEAK expression in the sarcopenia mouse model. (**A**) Forelimb grip strength (N) of young (*n* = 6) and old (*n* = 6) mice; (**B**) Fat mass (g) in young (*n* = 6) and old (*n* = 6) mice; (**C**) Lean mass (g) in young (*n* = 6) and old (*n* = 6) mice; (**D**) TWEAK mRNA levels in the muscles of young (*n* = 6) and old (*n* = 6) mice; (**E**) Quantitative analysis of TWEAK mRNA levels in the muscles of young (*n* = 3) and old (*n* = 3) mice; (**F**) Western blot of TWEAK protein levels in the muscles of young (*n* = 3) and old (*n* = 3) mice. Data are presented as mean ± standard deviation. * *P* < 0.05, ** *P* < 0.01, *** *P* < 0.001, Student’s t-test was used for statistical analysis.

Fifteen-month-old C57BL/6J mice were maintained under standard husbandry conditions until 18 months of age to establish a naturally aged sarcopenia model. Forelimb grip strength was significantly reduced in aged mice relative to young controls (Fig. 1A). No significant difference in fat mass was observed between the two groups (Fig. 1B), whereas lean mass was significantly greater in aged mice than in young controls (Fig. 1C). To investigate age-related alterations in TWEAK expression, we evaluated its expression profile. qRT-PCR revealed that TWEAK messenger RNA (mRNA) levels were significantly elevated in aged mice compared with young controls (Fig. 1D). Western blot analysis further indicated a significant increase in TWEAK protein levels in aged mice (Fig. 1E and F). Collectively, these findings demonstrate that TWEAK expression is upregulated in this sarcopenia mouse model.

### Knockdown of TWEAK could reduce dexamethasone-induced myotube atrophy and the response of inflammatory in C2C12 cells

**Fig. 2:**
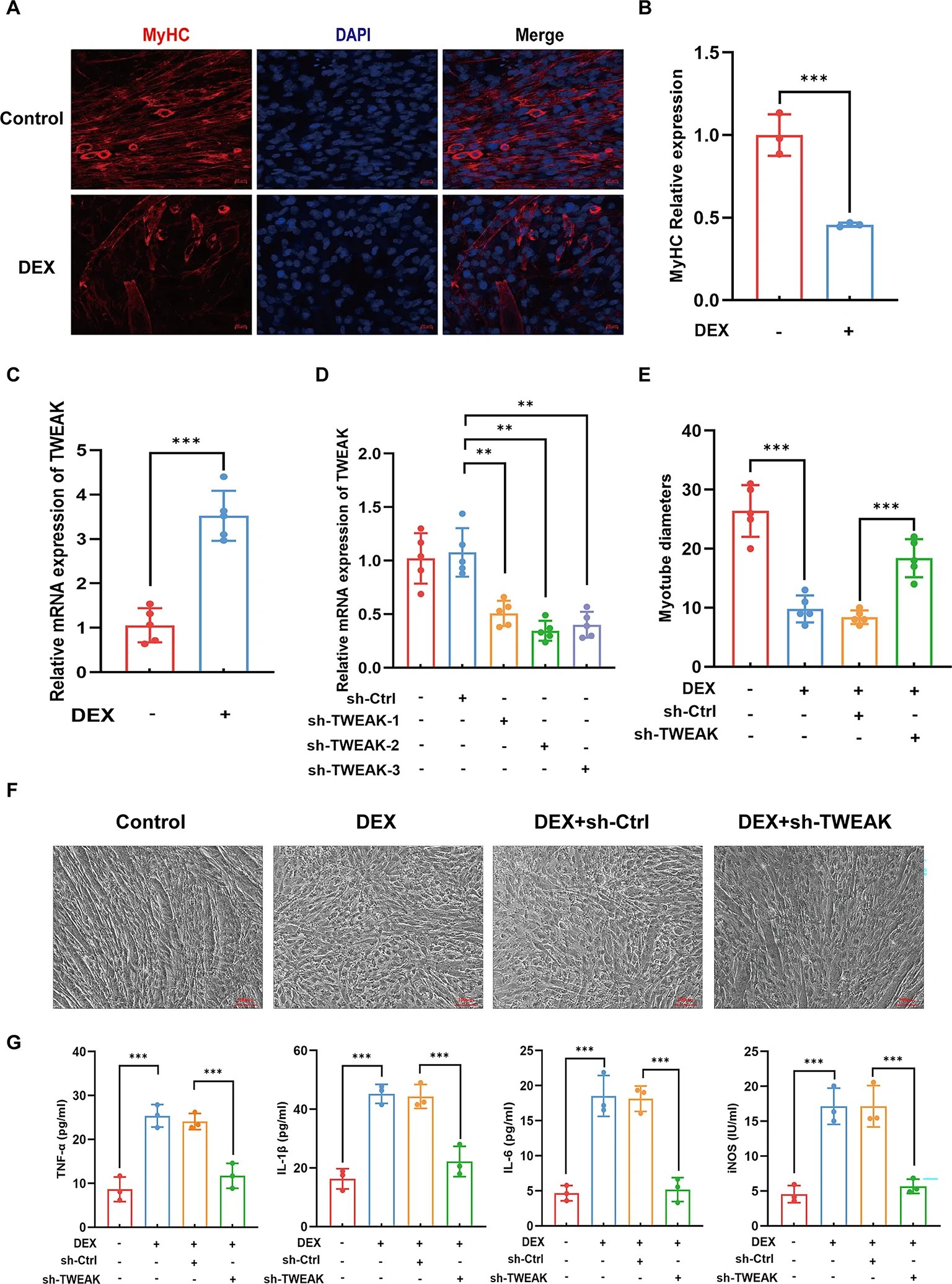
Knockdown of TWEAK reduces dexamethasone-induced myotube atrophy and inflammatory response in C2C12 cells. (**A**) Representative immunofluorescence images of the control group and DEX group (*n* = 3), Scale bar = 40 μm; (**B**) MyHC protein levels in the DEX group compared to the control group; (**C**) TWEAK mRNA levels after 24 h of 5 µM dexamethasone treatment, measured by qRT-PCR; (**D**) mRNA levels of sh-Ctrl, sh-TWEAK1, 2, 3 groups after 48 h of transfection, measured by qRT-PCR (*n* = 3); (**E**) C2C12 myotube diameter of the control group, DEX group, DEX + sh-Ctrl group, and DEX + sh-TWEAK group (*n* = 3); (**F**) Myotube images of the control group, DEX group, DEX + sh-Ctrl group, and DEX + sh-TWEAK group (*n* = 3), Scale bar = 50 μm; (**G**) ELISA analysis of TNF-α, IL-1β, IL-6, and iNOS levels in the control group, DEX group, DEX + sh-Ctrl group, and DEX + sh-TWEAK group (*n* = 3). Data are presented as mean ± standard deviation. * *P* < 0.05, ** *P* < 0.01, *** *P* < 0.001, Student’s t-test was used for two-group comparisons, and one-way ANOVA followed by Dunnett’s post-hoc test was used for multiple-group comparisons.

It should be acknowledged that dexamethasone-induced myotube atrophy and aging-related sarcopenia involve distinct pathogenic pathways. Accordingly, this section employed a pharmacologically induced muscle atrophy cell model to preliminarily investigate the regulatory effects of TWEAK on myotube atrophy and inflammation. Immunofluorescence results demonstrated that the MyHC was significantly down-regulated in the DEX group (Fig. 2A,B), indicating that dexamethasone induced atrophy in C2C12 myotubes. In the DEX group, TWEAK mRNA level was significantly up-regulated (Fig. 2C). In the C2C12 cell line, a TWEAK-EGFP lentiviral vector was constructed to knock down TWEAK (sh-TWEAK) or the control (sh-Ctrl). QRT-PCR results showed that, compared to sh-Ctrl, sh-TWEAK-2 significantly reduced TWEAK mRNA expression (Fig. 2D). Microscopic observation revealed that, compared to sh-Ctrl, sh-TWEAK promoted the formation of myotubes (Fig. 2E,F). Additionally, we examined the effect of TWEAK on inflammatory factors. The levels of TNF-α, IL-1β, IL-6, and iNOS in the culture supernatant of DEX-treated cells were significantly increased, whereas sh-TWEAK inhibited this effect caused by dexamethasone (Fig. 2G). These results indicate that knockdown of TWEAK reduces dexamethasone-induced myotube atrophy and inflammatory response in C2C12 cells.

### Knockdown of TWEAK promotes mitochondrial biogenesis in C2C12 Cells

**Fig. 3:**
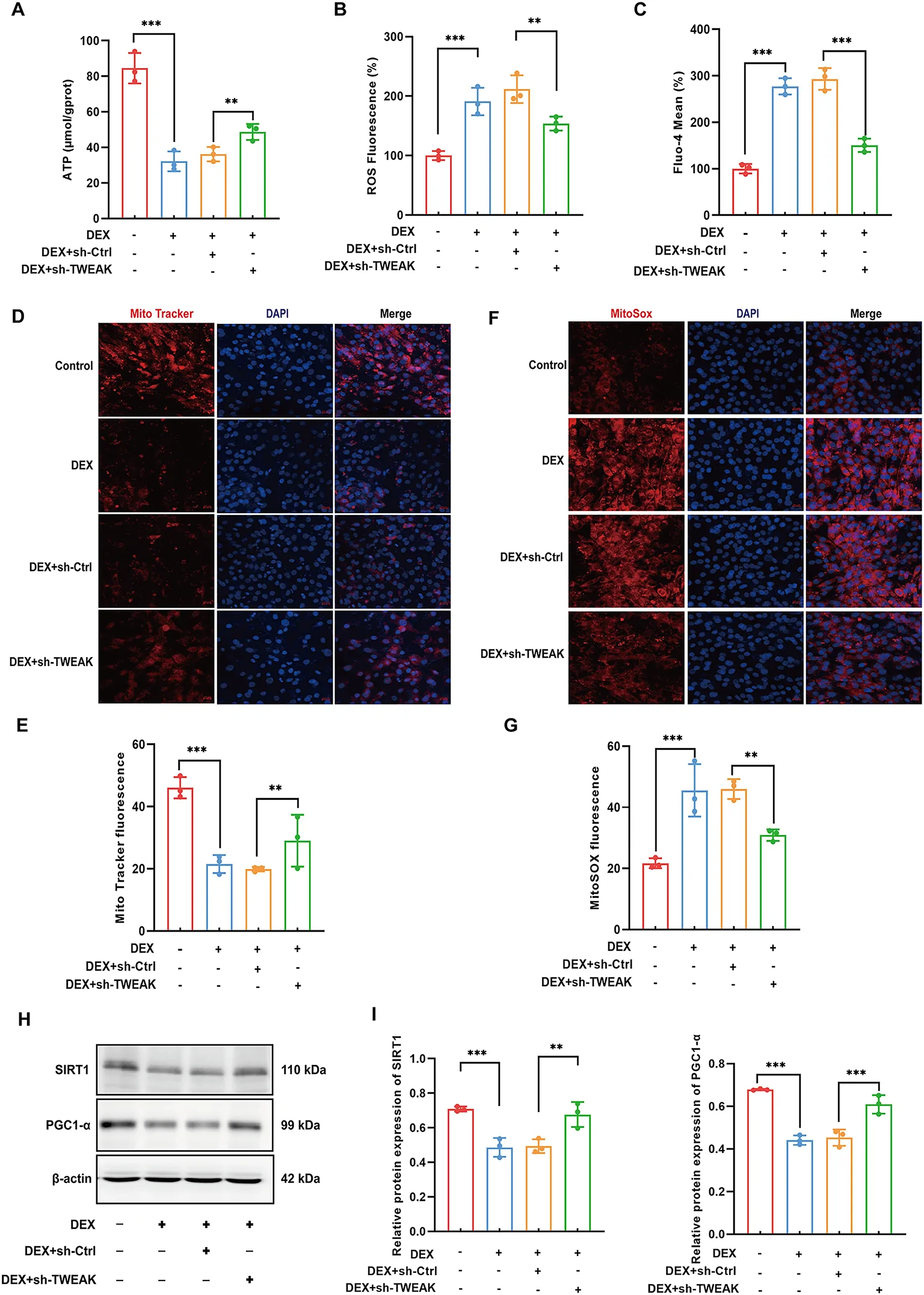
TWEAK inhibits mitochondrial biogenesis in C2C12 Cells. (**A**) ATP content in cells from the control, DEX, DEX + sh-Ctrl, and DEX + sh-TWEAK groups measured by a microplate reader (*n* = 3); (**B**) ROS levels in cells from the control, DEX, DEX + sh-Ctrl, and DEX + sh-TWEAK groups analyzed by a fluorescence microplate reader (*n* = 3); (**C**) Ca2 + levels in cells from the control, DEX, DEX + sh-Ctrl, and DEX + sh-TWEAK groups analyzed by Fluo-4 AM staining (*n* = 3); (**D**) Mitochondrial content in cells from the control, DEX, DEX + sh-Ctrl, and DEX + sh-TWEAK groups, scale bars = 20 μm; (**E**) Mitochondrial content in cells from the control, DEX, sh-Ctrl, and sh-TWEAK groups analyzed by MitoTracker staining (*n* = 3), scale bars = 40 μm; (**F**) Mitochondrial ROS levels in cells from the control, DEX, DEX + sh-Ctrl, and DEX + sh-TWEAK groups; (**G**) Mitochondrial ROS levels in cells from the control, DEX, DEX + sh-Ctrl, and DEX + sh-TWEAK groups analyzed by MitoSOX staining (*n* = 3). Scale bar = 40 μm; (**H**) Immunoblotting of SIRT1 and PGC-1α protein levels in cells from the control, DEX, DEX + sh-Ctrl, and DEX + sh-TWEAK groups; (**I**) Quantitative analysis of SIRT1 and PGC-1α protein levels in cells from the control, DEX, DEX + sh-Ctrl, and DEX + sh-TWEAK groups. Data are presented as mean ± standard deviation. * *P* < 0.05, ** *P* < 0.01, *** *P* < 0.001, One-way ANOVA followed by Dunnett’s post-hoc test was used for statistical analysis.

In the pathogenesis of sarcopenia, TWEAK inhibits mitochondrial biogenesis through downregulation of PGC-1α and its associated regulatory factors. Our experimental findings demonstrated that the DEX group exhibited significantly reduced ATP levels compared to the control group, accompanied by marked elevations in ROS and Ca²⁺ concentrations (*p* < 0.05, Fig. 3A–C). Notably, the DEX + sh-TWEAK group showed enhanced ATP production and suppressed ROS/Ca²⁺ accumulation relative to the DEX + sh-Ctrl group (*p* < 0.05, Fig. 3A–C), suggesting involvement of TWEAK in mitochondrial functional regulation. MitoTracker and MitoSOX staining analyses further revealed that the DEX group displayed decreased mitochondrial mass and elevated mitochondrial-specific ROS levels compared to controls (*p* < 0.05, Fig. 3D–G). In contrast, the DEX + sh-TWEAK group exhibited increased mitochondrial content and reduced mitochondrial ROS generation compared to the DEX + sh-Ctrl group (*p* < 0.05, Fig. 3D–G). As key regulators of mitochondrial biogenesis, SIRT1 and PGC-1α protein expression levels were significantly downregulated in the DEX group versus controls (*p* < 0.05, Fig. 3H,I). However, TWEAK knockdown effectively restored the expression of both proteins in the DEX + sh-TWEAK group compared to the DEX + sh-Ctrl group (*p* < 0.05, Fig. 3H,I). These collective findings indicate that TWEAK suppression promotes mitochondrial biogenesis in C2C12 myoblasts.

### The regulation of C2C12 myotube atrophy by TWEAK is accompanied by changes in the AMPK-p38 MAPK pathway

**Fig. 4:**
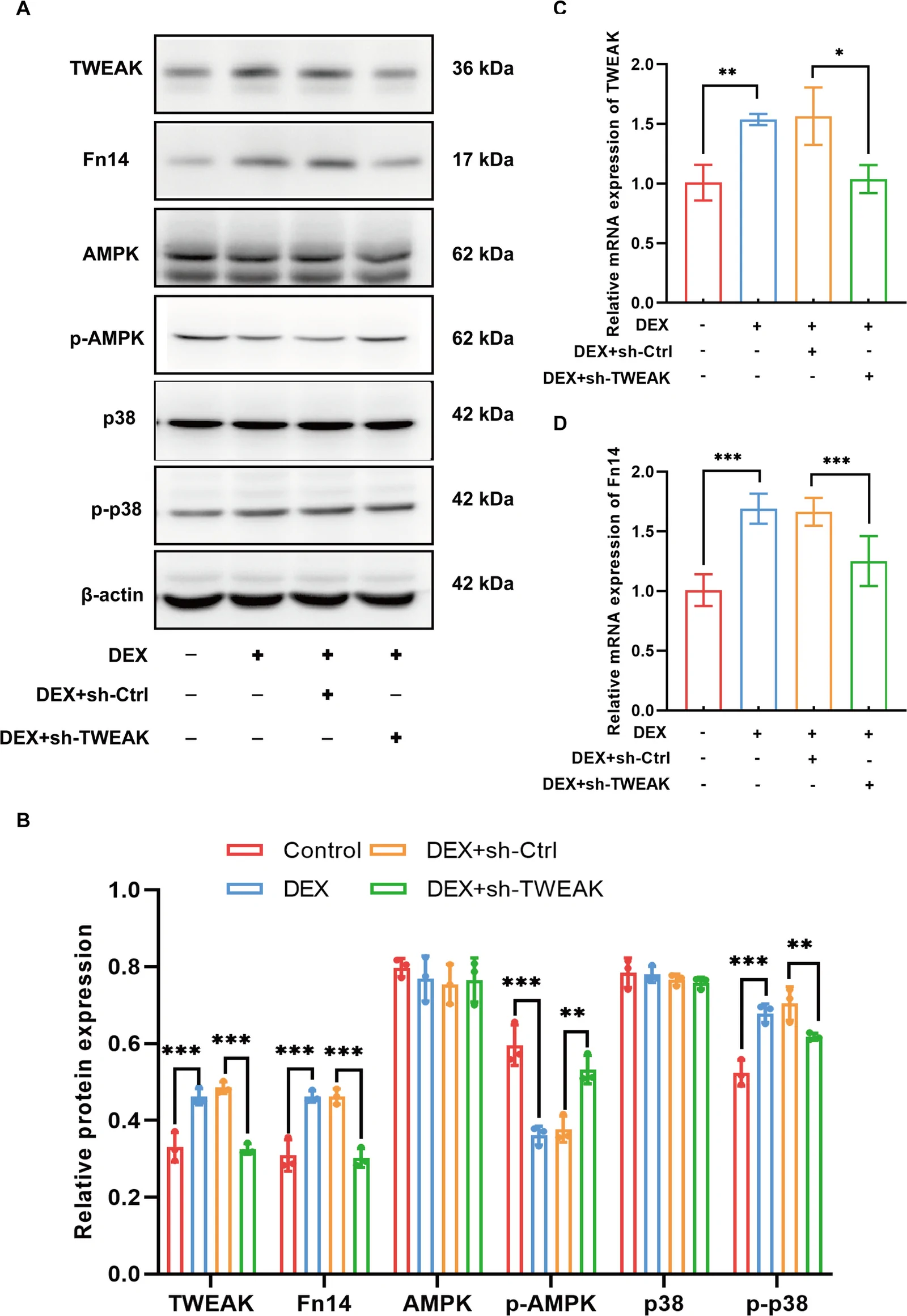
TWEAK is associated with C2C12 myotube atrophy, accompanied by changes in the activity of the AMPK-p38 MAPK pathway. (**A**) Immunoblotting of TWEAK, Fn14, AMPK, p-AMPK/AMPK, p-p38 and p38 protein levels in cells from the control, DEX, DEX + sh-Ctrl, and DEX + sh-TWEAK groups (*n* = 3); (**B**) Quantification of immunoblot signals normalized to β-actin loading control (*n* = 3); (**C**) QRT-PCR detection of TWEAK mRNA levels in cells from the control, DEX, DEX + sh-Ctrl, and DEX + sh-TWEAK groups (*n* = 3); (**D**) QRT-PCR detection of Fn14 mRNA levels in cells from the control, DEX, DEX + sh-Ctrl, and DEX + sh-TWEAK groups (*n* = 3). Data are presented as mean ± standard deviation. * *P* < 0.05, ** *P* < 0.01, *** *P* < 0.001, One-way ANOVA followed by Dunnett’s post-hoc test was used for statistical analysis.

TWEAK and Fn14 play crucial roles in various biological processes, and both of them significantly up-regulated in the DEX group. As compared with sh-Ctrl group, the protein (Fig. 4A,B) and mRNA level (Fig. 4C,D) of TWEAK and Fn14 were significantly down-regulated in sh-TWEAK group. Furthermore, we found that protein level of p-AMPK was reduced, and p-p38 protein expression was increased in the DEX group. In contrast, compared to the sh-Ctrl group, the protein expression of p-AMPK was increased and p-p38 protein expression was decreased in the sh-TWEAK group (Fig. 4A,B). These results indicate that TWEAK may promote the atrophy of C2C12 myotubes upon binding to its receptor, an effect associated with altered AMPK-p38 MAPK signaling.

### Knockdown of TWEAK enhances muscle mass and inhibits inflammatory response in old mice

**Fig. 5:**
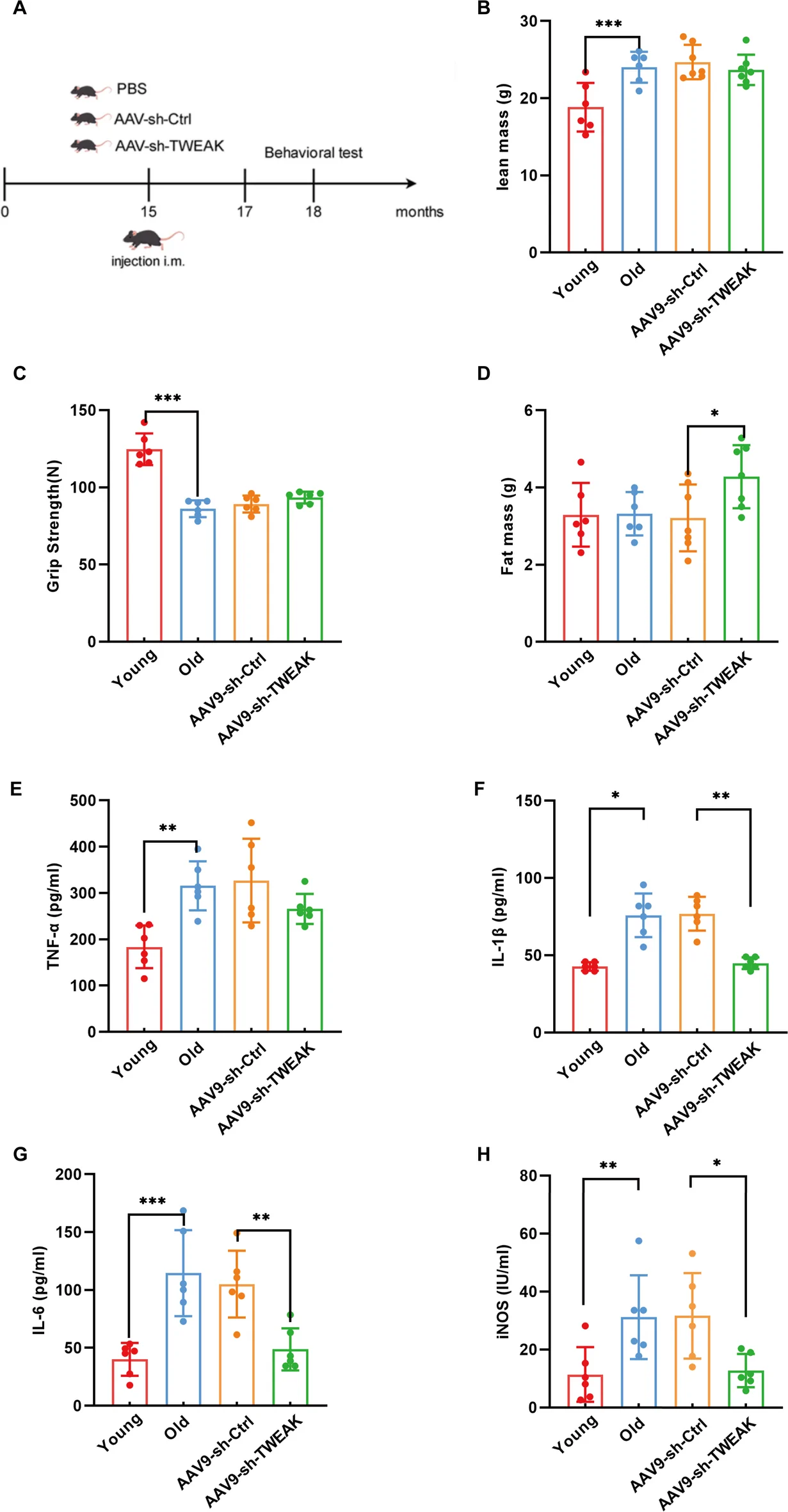
Knockdown of TWEAK enhances muscle mass and inhibits inflammatory response in old mice. (**A**) Schematic diagram of the time schedule for AAV-sh-Ctrl and AAV-TWEAK injections; (**B**) Forelimb grip strength (N) of young (*n* = 6), old (*n* = 6), AAV-sh-Ctrl (*n* = 6), and AAV-TWEAK (*n* = 6) groups; (**C**) Fat content (g) in young (*n* = 6), old (*n* = 6), AAV-sh-Ctrl (*n* = 6), and AAV-TWEAK (*n* = 6) groups; (**D**) Muscle mass (g) in young (*n* = 6), old (*n* = 6), AAV-sh-Ctrl (*n* = 6), and AAV-TWEAK (*n* = 6) groups; (**E**) ELISA analysis of serum TNF-α level in young (*n* = 6), old (*n* = 6), AAV-sh-Ctrl (*n* = 6), and AAV-TWEAK (*n* = 6) groups; (**F**) ELISA analysis of serum IL-1β level in young (*n* = 6), old (*n* = 6), AAV-sh-Ctrl (*n* = 6), and AAV-TWEAK (*n* = 6) groups; (**G**) ELISA analysis of serum IL-6 level in young (*n* = 6), old (*n* = 6), AAV-sh-Ctrl (*n* = 6), and AAV-TWEAK (*n* = 6) groups; (**H**) ELISA analysis of serum iNOS level in young (*n* = 6), old (*n* = 6), AAV-sh-Ctrl (*n* = 6), and AAV-TWEAK (*n* = 6) groups. Data are presented as mean ± standard deviation. * *P* < 0.05, ** *P* < 0.01, *** *P* < 0.001, One-way ANOVA followed by Dunnett’s post-hoc test was used for statistical analysis.

We further conducted in vivo studies to investigate whether knockdown of TWEAK increases muscle mass and inhibits the inflammatory response in mice. As shown in the animal experimental flow chart in Fig. 5A, AAV-GFP control or AAV-TWEAK shRNA viral vectors were injected into the bilateral gastrocnemius muscles of old mice at 15 and 17 months of age. After completing behavioral tests at 18 months of age, all mice were sacrificed and tissue samples were harvested. Compared with the AAV-sh-Ctrl group, old mice in the AAV-TWEAK group exhibited a significant increase in fat mass (*P* < 0.05; Fig. 5D), whereas forelimb grip strength and lean mass showed no significant differences (*P* > 0.05; Fig. 5B and C). Subsequently, the impact of TWEAK on systemic inflammation was evaluated. Serum levels of IL-1β, IL-6, and iNOS were significantly reduced in the AAV-TWEAK group relative to the AAV-sh-Ctrl group. Conversely, TNF-α levels did not differ significantly between the two groups (Fig. 5E–H). These results suggest that knockdown of TWEAK enhances muscle mass and inhibits the inflammatory response in old mice.

### Knockdown of TWEAK improves mitochondrial biogenesis in the skeletal muscle of aged mice, and is accompanied by regulatory changes in the AMPK-p38 MAPK pathway

**Fig. 6:**
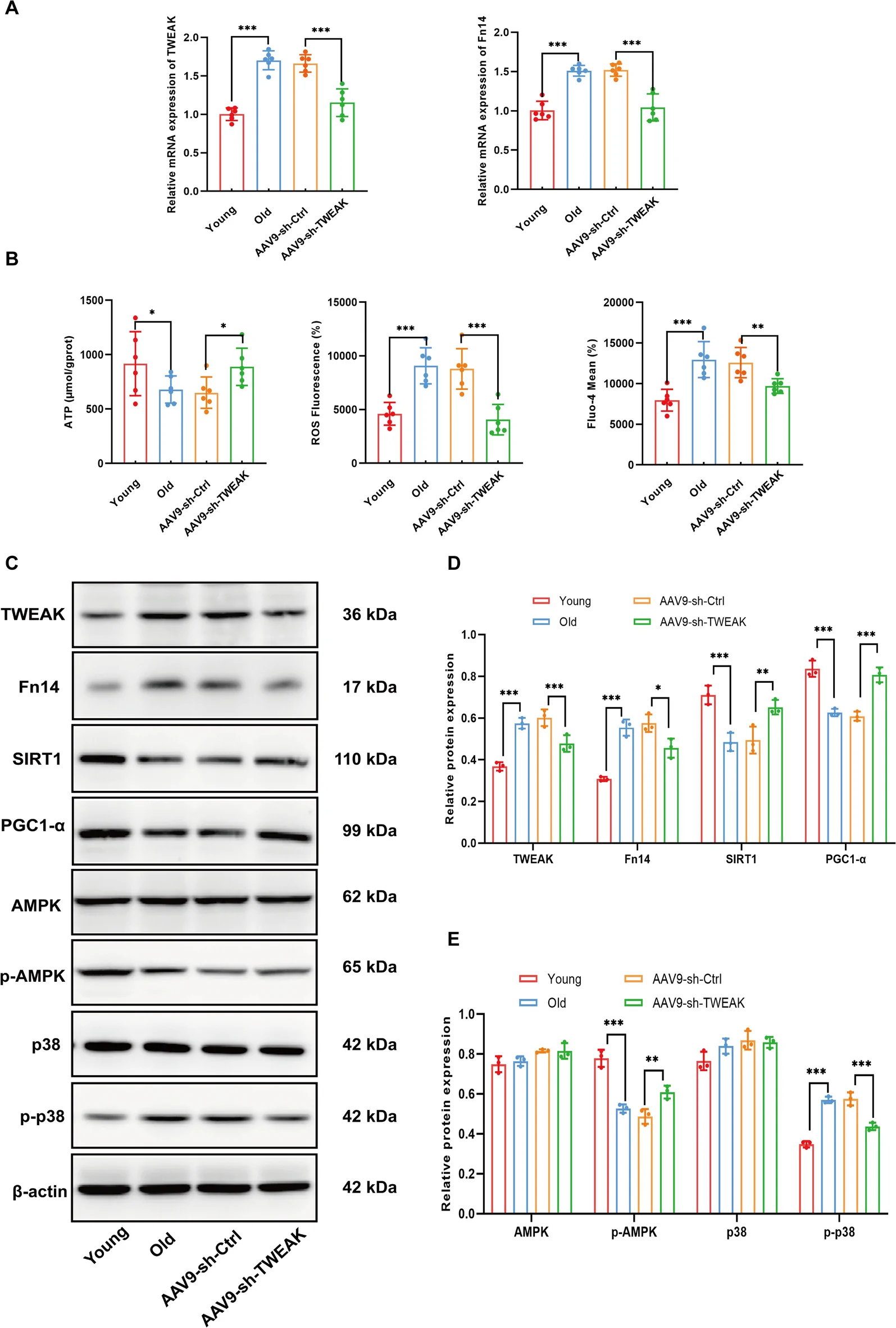
Knockdown of TWEAK is associated with improved mitochondrial biogenesis in the skeletal muscle of aged mice, as well as with altered AMPK-p38 MAPK signaling. (**A**) QRT-PCR detection of TWEAK and Fn14 mRNA levels in the gastrocnemius muscle of young (*n* = 6), old (*n* = 6), AAV-sh-Ctrl (*n* = 6), and AAV-TWEAK (*n* = 6) groups; (**B**) ATP, ROS, and Ca2 + levels in the gastrocnemius muscle of young (*n* = 6), old (*n* = 6), AAV-sh-Ctrl (*n* = 6), and AAV-TWEAK (*n* = 6) groups; (**C**) Immunoblotting of TWEAK, Fn14, SIRT1, PGC1-α protein levels in the gastrocnemius muscle of young (*n* = 3), old (*n* = 3), AAV-sh-Ctrl (*n* = 3), and AAV-TWEAK (*n* = 3) groups; (**D**) Immunoblotting of p-AMPK/AMPK and p-p38/p38 protein levels in the gastrocnemius muscle of young (*n* = 3), old (*n* = 3), AAV-sh-Ctrl (*n* = 3), and AAV-TWEAK (*n* = 3) groups; (**E**) Quantitative analysis of TWEAK, Fn14, SIRT1, PGC1-α, p-AMPK/AMPK, and p-p38/p38 protein levels in the gastrocnemius muscle of young (*n* = 3), old (*n* = 3), AAV-sh-Ctrl (*n* = 3), and AAV-TWEAK (*n* = 3) groups. Data are presented as mean ± standard deviation. * *P* < 0.05, ** *P* < 0.01, *** *P* < 0.001, One-way ANOVA followed by Dunnett’s post-hoc test was used for statistical analysis.

To determine the specific mechanistic role of TWEAK in sarcopenia mice, we further analyzed changes in the AMPK-p38 MAPK signaling pathway and mitochondrial biogenesis-related proteins. Real-time quantitative PCR and western blotting results demonstrated that both mRNA and protein levels of TWEAK and Fn14 were significantly upregulated in the Old group compared with the Young group (*p* < 0.05, Fig. 6A,C and D). Conversely, TWEAK and Fn14 mRNA and protein levels were significantly reduced in old mice of the AAV9-sh-TWEAK group compared with the AAV9-sh-Ctrl group (*p* < 0.05; Fig. 6A and C and D). Functional analysis revealed that skeletal muscle ATP content was significantly decreased in old mice compared with the Young group, whereas ROS and Ca²⁺ concentrations were elevated (*p* < 0.05; Fig. 6B). Notably, AAV9-sh-TWEAK intervention effectively restored ATP levels and attenuated oxidative stress and calcium dysregulation (Fig. 6B). Further western blot analysis identified significant upregulation of SIRT1, PGC-1α, and p-AMPK protein expression, along with decreased p-p38 levels in the AAV9-sh-TWEAK group compared with the AAV9-sh-Ctrl group (*p* < 0.05, Fig. 6C and E). These results suggest that knockdown of TWEAK was associated with improved mitochondrial biogenesis in the skeletal muscle of old mice, as well as with changes in AMPK and p38 MAPK activity that may contribute to this process.

## Discussion

Muscle strength remains a primary clinical parameter for diagnosing the severity of sarcopenia. In our study, naturally old mice exhibited a marked decline in forelimb grip strength compared with young controls. Besides, no significant difference in fat mass between young and old mice, whereas lean mass was significantly greater in the aged group. These phenotypic changes coincided with a robust upregulation of TWEAK and Fn14 expression in skeletal muscle, supporting a pathogenic role for this signaling axis in age-related muscle deterioration. Both dexamethasone-induced C2C12 myotube atrophy and the impaired muscle performance observed in aged mice were associated with elevated TWEAK/Fn14 levels. Consistent with our findings, Tajrishi et al. reported elevated Fn14 expression in the skeletal muscle of 18-month-old (aged) mice compared with adult counterparts, and demonstrated that Fn14 ablation significantly increased specific muscle protein levels while ameliorating age-related myofiber atrophy^23^. Notably, our results also differ from previous observations in denervation-induced muscle atrophy models, in which only Fn14 upregulation was detected without significant alterations in TWEAK expression^24–26^. This discrepancy may be explained by the heterogeneity of model types: distinct differences exist in the pathological microenvironment, inflammatory status, and molecular regulatory network among denervation-induced muscle atrophy, drug-induced muscle atrophy, and natural aging-related sarcopenia, which consequently result in inconsistent expression patterns of TWEAK and Fn14. These findings suggest that TWEAK knockdown can attenuate age-related muscle mass loss in old mice, although it did not significantly improve forelimb grip strength or alter fat content in the in vivo intervention experiment. Therefore, inhibiting the TWEAK/Fn14 axis may represent a potential strategy for preserving muscle mass and mitochondrial function during aging, though its capacity to restore neuromuscular strength remains to be established.

Notably, the TWEAK-Fn14 axis also exerts important physiological roles in skeletal muscle. TWEAK has been reported to promote myotube formation and enhance muscle regeneration, whereas loss of TWEAK reduces myoblast fusion efficiency^27^. Fn14 signaling positively regulates myoblast fusion and skeletal muscle regeneration; deletion of Fn14 in myoblasts impairs fusion despite accelerating initial differentiation, while overexpression of Fn14 promotes the formation of myotubes with increased diameter^28^. Moreover, Fn14 is required for satellite cell self-renewal and proliferation, and its conditional ablation in Pax7-expressing satellite cells drastically reduces their expansion and skeletal muscle regeneration following injury, exacerbates myopathy, whereas Fn14 overexpression improves engraftment of exogenous muscle progenitor cells into dystrophic muscle^29^. These observations collectively suggest that the TWEAK-Fn14 system possesses context-dependent functions: it is indispensable for myogenic fusion and satellite cell-mediated regeneration under physiological or injury conditions, yet its chronic elevation in aged muscle appears to contribute to catabolic signaling and atrophy, consistent with the protective effects of TWEAK knockdown observed in the present work.

Mitochondria play a crucial role in energy metabolism within skeletal muscle cells. Mitochondrial damage leads to various cellular dysfunctions, exacerbating muscle loss and weakness^30^. Although evidence indicates that TWEAK and Fn14 expression is significantly increased in denervation-induced muscle atrophy^6^, and the TWEAK/Fn14 pathway is involved in muscle proliferation and differentiation^5^, the relationship between mitochondria and the activation of TWEAK/Fn14 pathway in sarcopenia remains unclear. Interestingly, our study found that the knockdown of TWEAK promoted an increase in mitochondrial number and reduced mitochondrial ROS levels. During aging, reduced RNA and protein expression of PGC-1α have previously been reported^31^. The activity of SIRT1, as a key regulator promoting the stabilization of mitochondria in skeletal muscle, also decreases with aging^32^. Furthermore, our results show down-regulated SIRT1 and PGC-1α in the sarcopenia model. Meanwhile, TWEAK knockdown could upregulate the protein levels of both SIRT1 and PGC-1α. These results provide evidence for the hypothesis that TWEAK regulates mitochondrial biogenesis by suppressing SIRT1 and PGC-1α protein levels.

Increasing evidence suggests that AMPK plays a specific regulatory role in mitochondrial biology and various aspects of homeostasis. AMPK is a highly conserved serine/threonine protein kinase that regulates bioenergy metabolism^33^. During energy stress, AMPK, as an upstream regulator, could upregulate PGC-1α, and then, control mitochondrial biogenesis^34^. Activation of AMPK is associated with various beneficial effects on skeletal muscle, while AMPK phosphorylation inhibition might promote the onset of sarcopenia by inhibiting PGC-1α expression^35–37^. Furthermore, mitochondrial translocation and apoptosis could be caused by activation of p38^33^. Activation of the MAPK pathway can inhibit the upregulation of mitochondrial biogenesis proteins, thereby reducing mitochondrial function and energy metabolism in skeletal muscle^38^. Our results show that in the sarcopenia model, p-AMPK expression was reduced, while p-p38 expression was increased, and TWEAK knockdown reversed these alterations. It has been reported that the TWEAK/Fn14 axis can regulate the activation of the p38 MAPK and AMPK pathways^39,40^. These findings indicate that in the context of sarcopenia, TWEAK/Fn14 signaling may modulate mitochondrial biogenesis and inflammatory responses through coordinated regulation of the AMPK-p38 MAPK axis. Additionally, TNF-α and IL-1β, as pro-inflammatory factors, could also be increased mediated by these two signaling pathways. Therefore, our findings support a potential link between TWEAK/Fn14 and the AMPK-p38 MAPK pathway in sarcopenia, where TWEAK may modulate mitochondrial biogenesis and inflammatory responses in coordination with this signaling axis.

Notably, the absence of grip strength recovery, despite significant gains in gastrocnemius muscle mass, indicates that TWEAK knockdown partially attenuates age-related muscle atrophy rather than fully reversing sarcopenia under the present experimental conditions. Several factors may account for this dissociation. First, the AAV9-sh-TWEAK vector was delivered specifically into the gastrocnemius, whereas forelimb grip strength depends on the coordinated function of multiple forelimb muscle groups, tendons, and connective tissues that were not targeted by the intervention. Second, aging-related declines in muscle strength are not always proportional to loss of muscle mass; the phenomenon of dynapenia underscores the contribution of neuromuscular deterioration (including motor unit remodeling, neuromuscular junction instability, and impaired neural drive) that may persist even after partial restoration of muscle mass. Localized TWEAK silencing in muscle fibers is unlikely to reverse these upstream neurological deficits. Third, the 18-month-old mouse model represents relatively early-stage aging, and the neuromuscular decrements present at this stage or in more advanced old age may be refractory to localized myocellular interventions alone. This suggests that the metabolic phenotype of our aged model was characterized by stable adiposity rather than obesity, and that TWEAK targeting specifically influenced muscle mass without substantially affecting fat deposition. Collectively, these considerations emphasize that while TWEAK–Fn14-mediated signaling is an important mediator of muscle catabolism and mitochondrial decline, effective restoration of strength in age-related muscle wasting may require systemic or multimodal interventions that concurrently address both myofiber preservation and neuromuscular integrity.

Dexamethasone-induced C2C12 myotube atrophy and natural aging sarcopenia are two distinct pathological models. The former is mainly driven by activation of the glucocorticoid receptor–Foxo/atrogin pathway, while the latter involves multifactorial alterations including chronic inflammation, neuromuscular junction degeneration, and satellite cell dysfunction. This study found that TWEAK expression was upregulated in both models, confirming that TWEAK is a common regulatory molecule in the progression of muscle atrophy. Nevertheless, the downstream regulatory networks of the two models are not completely equivalent. Therefore, the results of in vitro cell experiments can only serve as a mechanistic reference for the in vivo aging model, and cannot be directly extrapolated to fully explain the entire pathological process of age-related sarcopenia.

This study has several limitations. First, there is a mismatch between the functional assessment parameters and the site of muscular intervention. Although AAV9-sh-TWEAK was locally injected into the bilateral gastrocnemius muscles of the hindlimbs, forelimb grip strength was employed as the sole indicator of overall muscular function. Forelimb grip strength reflects only the integrated function of the systemic skeletal musculature and cannot directly quantify the contractile capacity or motor performance of the intervened gastrocnemius muscles. Consequently, this approach inadequately demonstrates the ameliorative effects of local TWEAK knockdown on target muscle function. Future experiments should therefore incorporate plantar flexor strength measurements and treadmill endurance tests to specifically assess gastrocnemius contractile force and exercise endurance, thereby comprehensively evaluating the beneficial effects of TWEAK silencing on local function of the hindlimb target muscles. Second, although our data demonstrate associations among TWEAK knockdown, AMPK-p38 MAPK pathway activity, and mitochondrial biogenesis, direct mechanistic validation through pharmacological or genetic inhibition of AMPK or p38 MAPK is required to confirm pathway dependency. Third, the upstream regulatory mechanisms governing TWEAK expression, such as DNA methylation, as well as its broader effects on mitochondrial dynamics and autophagy, were not fully elucidated. Fourth, the present study utilized 18-month-old C57BL/6J mice as an aging model. While mice at this age already displayed decreased grip strength and muscle mass relative to young controls, 18 months corresponds to the early onset of the aging process in mice, and 22–26 months is generally regarded as the more appropriate age for investigating advanced, senescence-associated sarcopenia. Consequently, the pathological phenotypes and molecular alterations observed in our model may not completely mirror those seen in more advanced old age, and further studies employing older mice are needed to confirm the generalizability and therapeutic potential of TWEAK inhibition in late-onset sarcopenia. Fifth, only male mice were used in in vivo experiments, which limits the sex-related generalizability of our results. Given the higher prevalence of sarcopenia in females and the modulatory effects of sex hormones on muscle homeostasis, our conclusions cannot be directly extrapolated to female populations. Future studies including both sexes are needed to evaluate potential sex-specific responses to TWEAK targeting. Additionally, this study employed both dexamethasone-induced muscle atrophy and natural aging sarcopenia models; given the divergent pathological pathways between these two models, the mechanistic interpretations derived from in vitro experiments have certain limitations when extrapolated to in vivo aging sarcopenia.

## Conclusion

Altogether, our data demonstrated that silencing TWEAK attenuates age-related loss of muscle mass and improves mitochondrial biogenesis in aged mice. These beneficial effects correlate with altered expression of SIRT1 and PGC-1α and are accompanied by changes in AMPK-p38 MAPK signaling activity. However, TWEAK knockdown did not yield a statistically significant improvement in forelimb grip strength under the current experimental conditions. Therefore, while TWEAK may represent a valuable biological target for ameliorating age-related muscle atrophy and mitochondrial dysfunction, its therapeutic potential for fully reversing sarcopenia and for restoring overall physical function requires further investigation.

## Supplementary Information

Below is the link to the electronic supplementary material.

Supplementary Material 1

Supplementary Material 2

## Data Availability

All data generated or analysed during this study are included in this article.

## Abbreviations

RT-PCR: Real-time polymerase chain reaction
Fn14: Factor-inducible 14
PGC-1α: Peroxisome proliferator-activated receptor gamma coactivator 1-alpha
SIRT1: Sirtuin 1
DEXA: Dual-energy X-ray absorptiometry
DEX: Dexamethasone
PVDF: Polyvinylidene fluoride
TBS: Tris-buffered saline
ANOVA: Analysis of variance
