# Supplementary material for: Targeting TWEAK to enhance mitochondrial biogenesis and attenuate age-related muscle mass loss
Source: Sci Rep. 2026 Aug 14;16:28221. doi: 10.1038/s41598-026-64401-2 (PMC13558719; doi:10.1038/s41598-026-64401-2)
Supplement: Supplementary file 1 [file 41598_2026_64401_MOESM1_ESM.pdf]

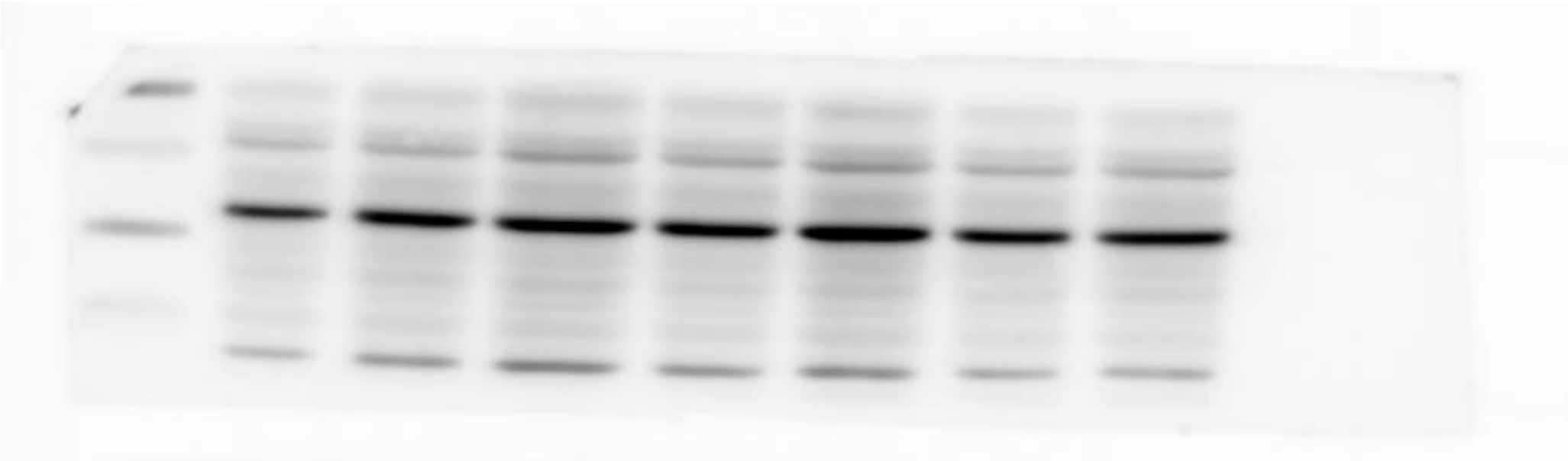

Figure 1G-TWEAK Repeat 1

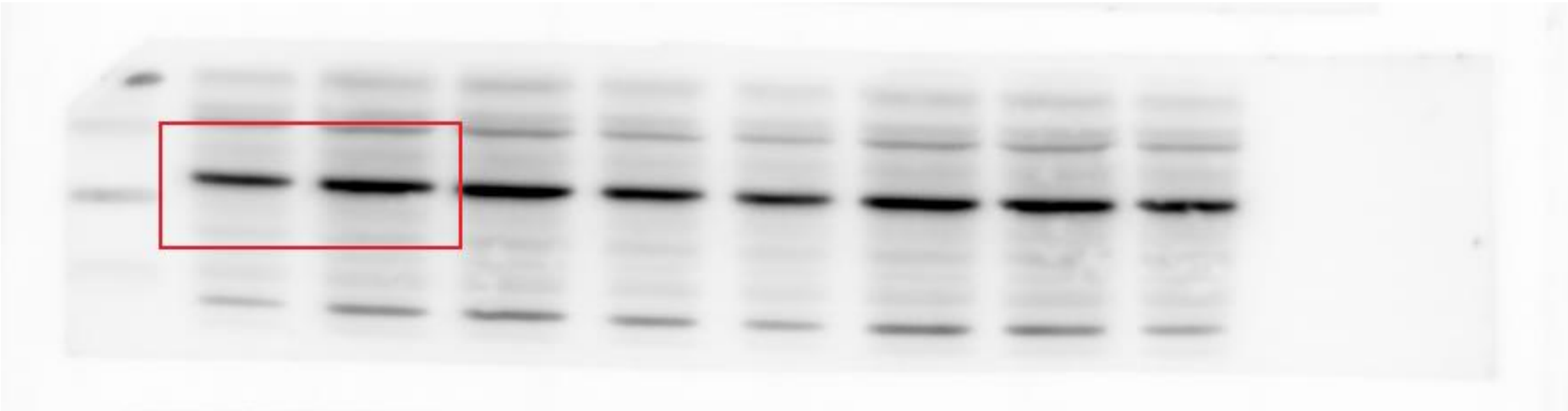

Figure 1G-TWEAK Repeat 2 and 3

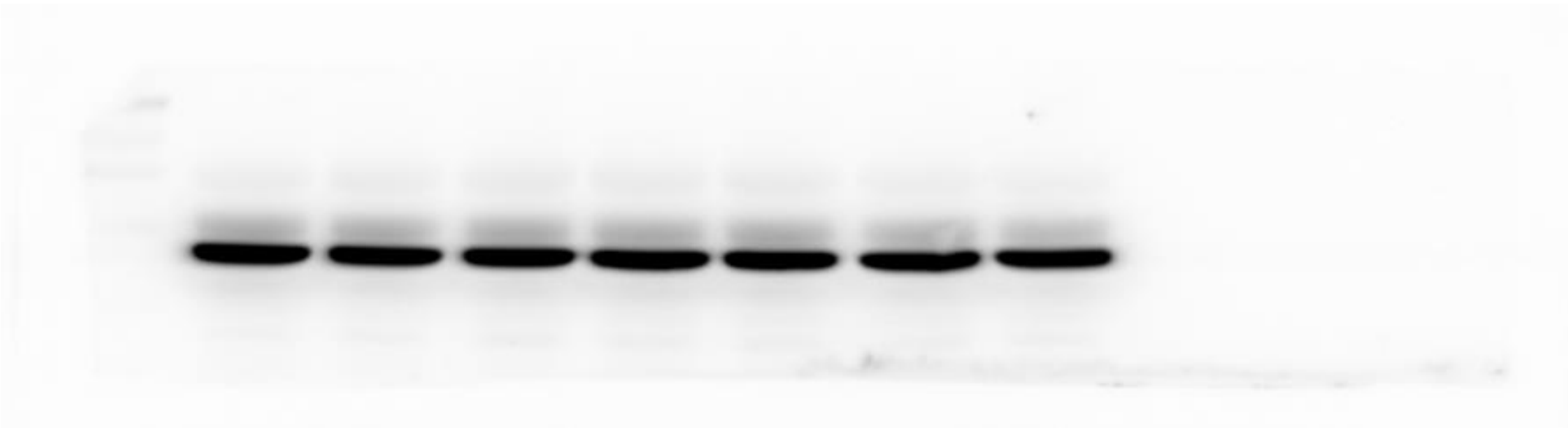

Figure 1G-β-actin Repeat 1

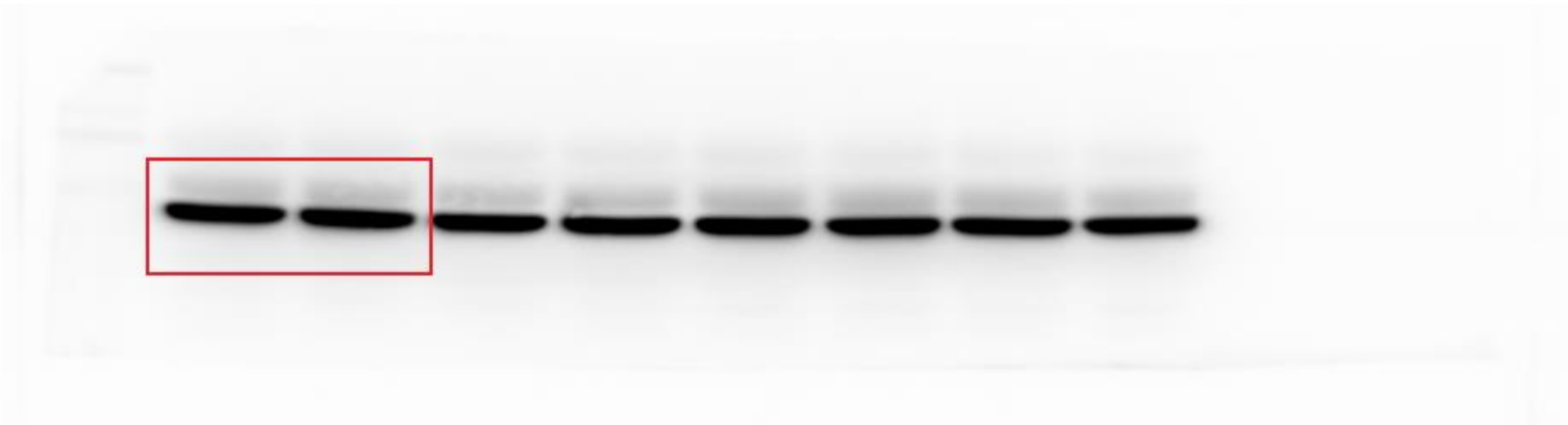

Figure 1-G-β-actin Repeat 2 and 3

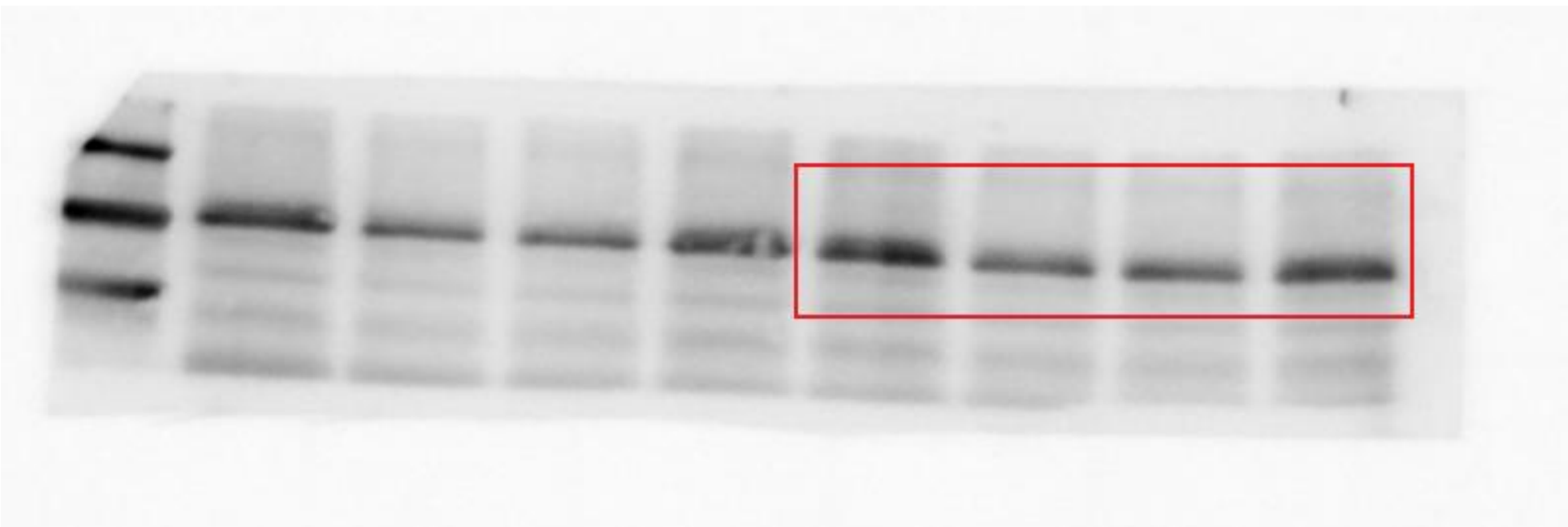

Figure 3H-SIRT1-Repeat 1

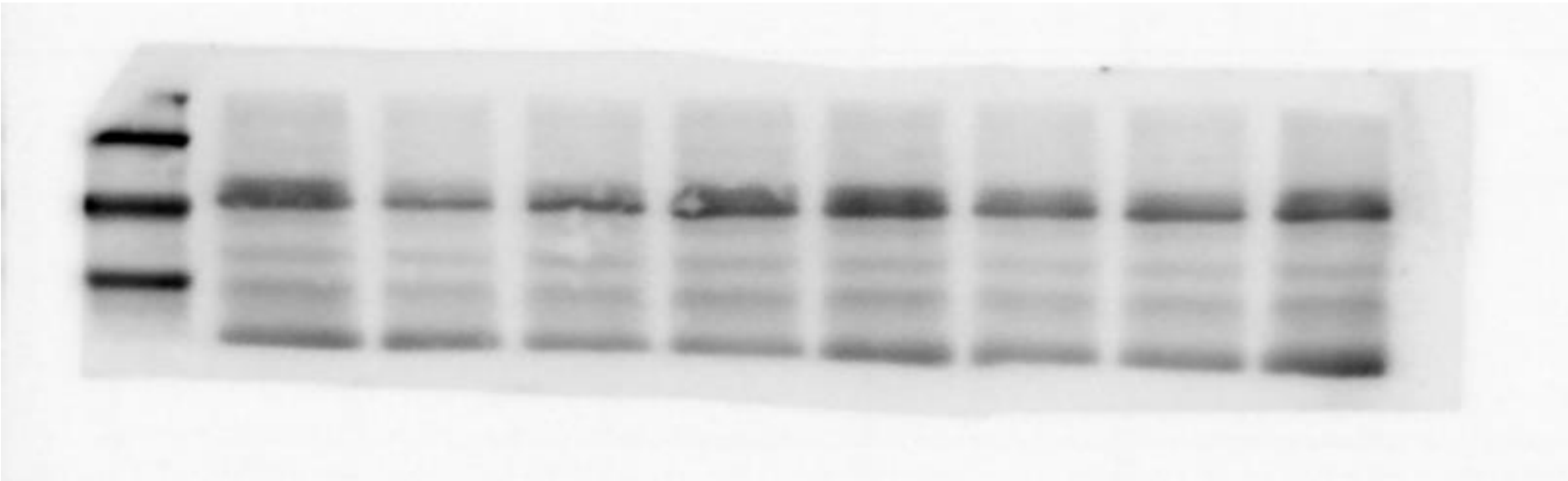

Figure 3H-SIRT1-Repeat 2

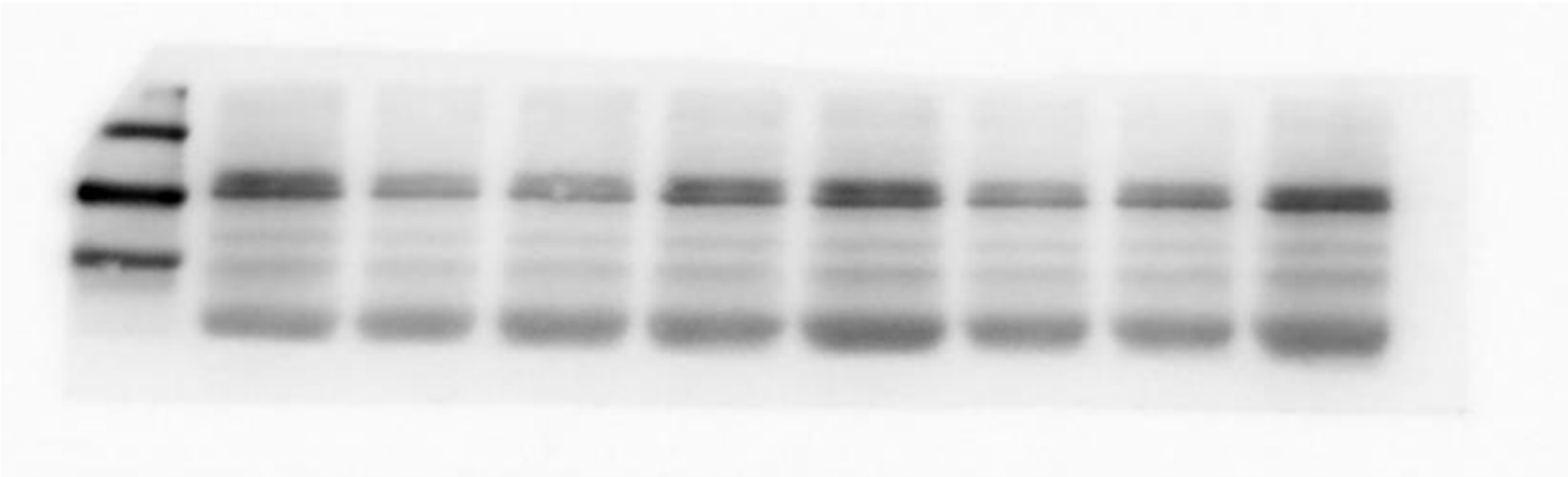

Figure 3H-SIRT1-Repeat 3

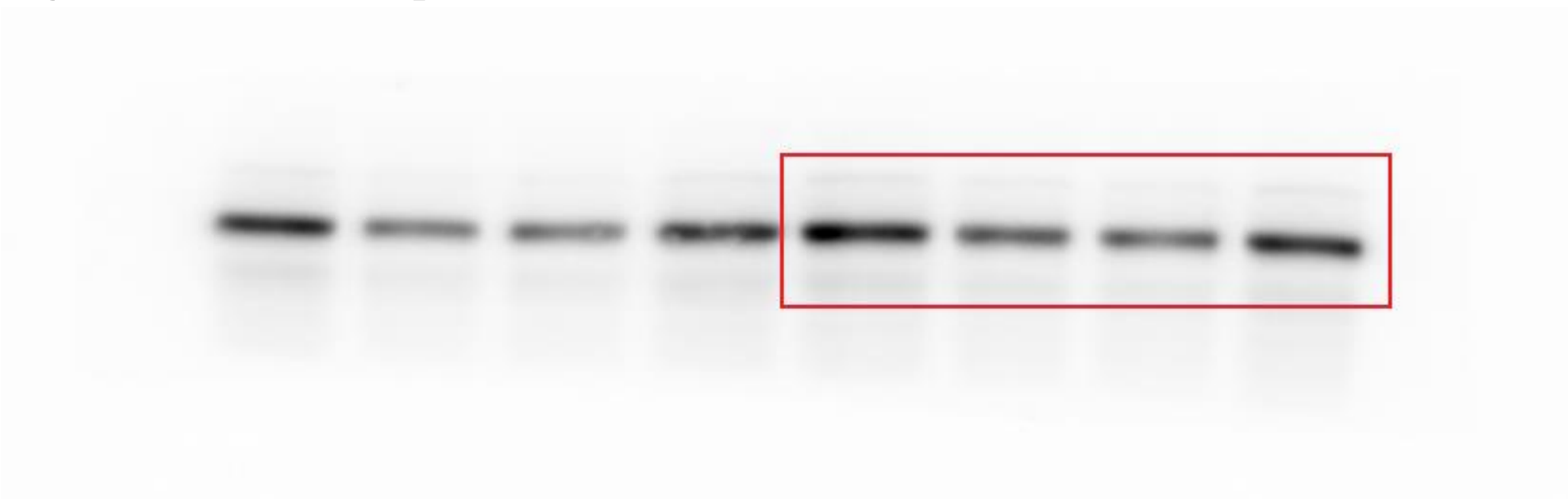

Figure 3H-PGCα-Repeat 1

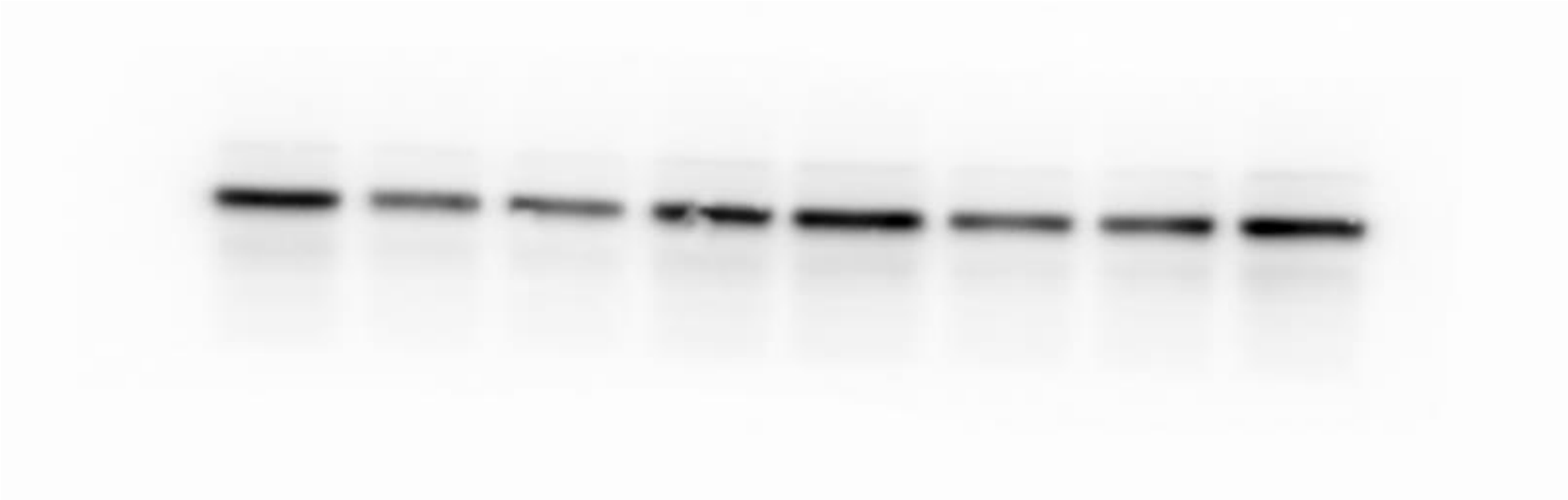

Figure 3H-PGCα-Repeat 2

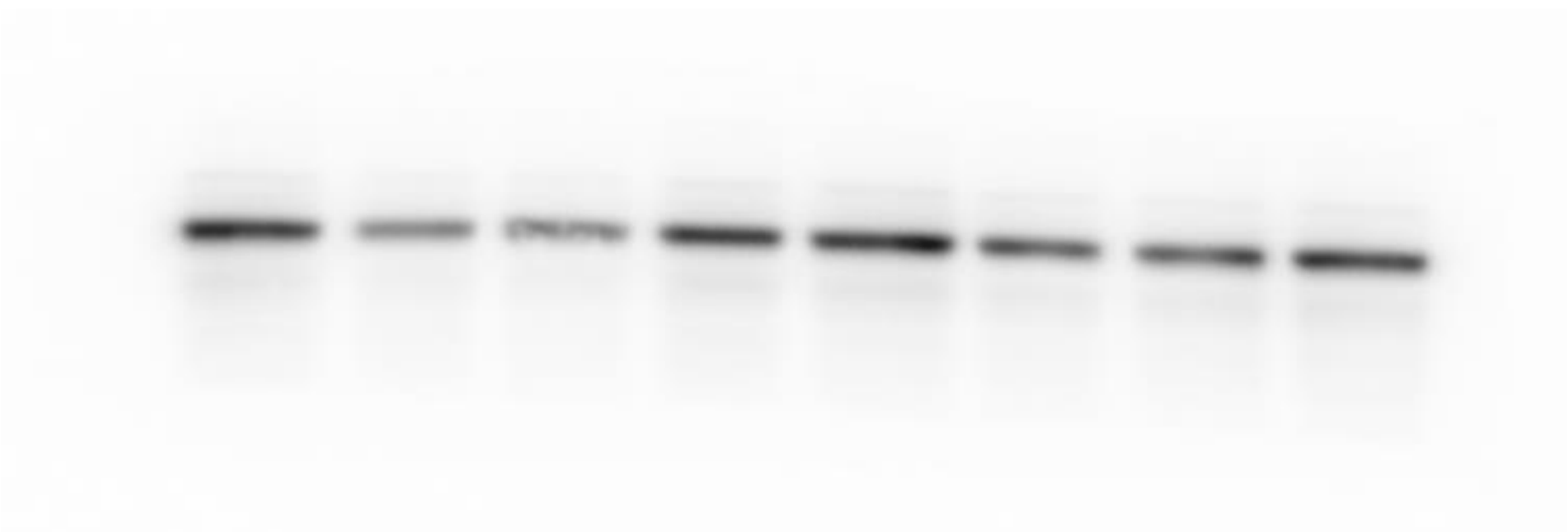

Figure 3H-PGCα-Repeat 3

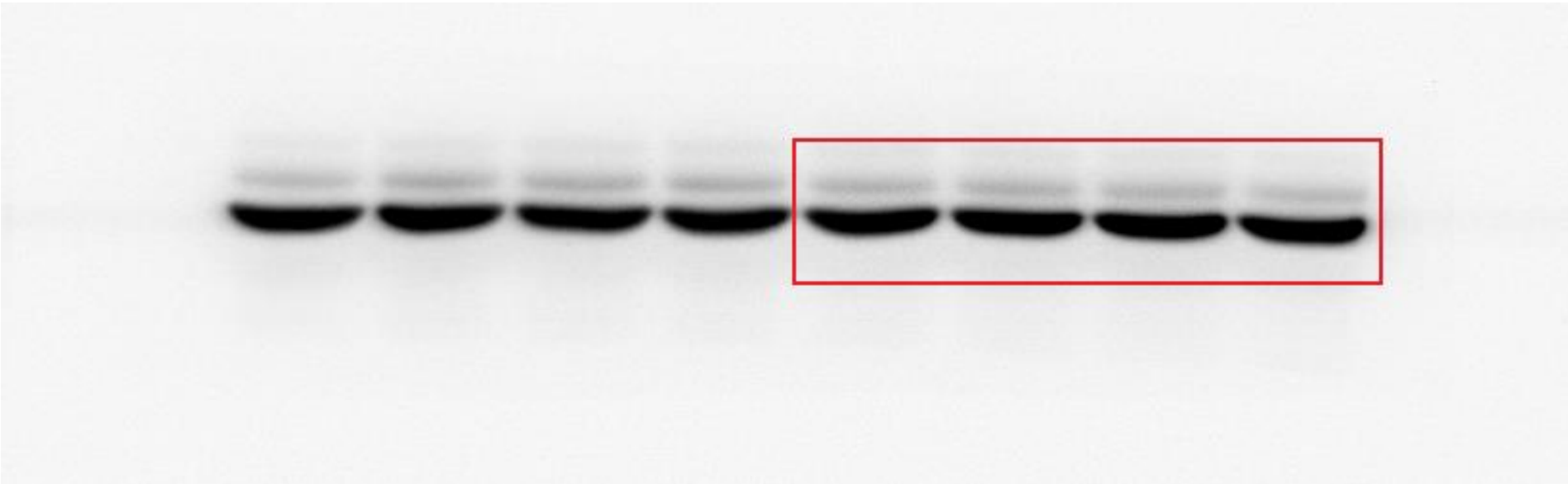

Figure 3H-β-actin-Repeat 1

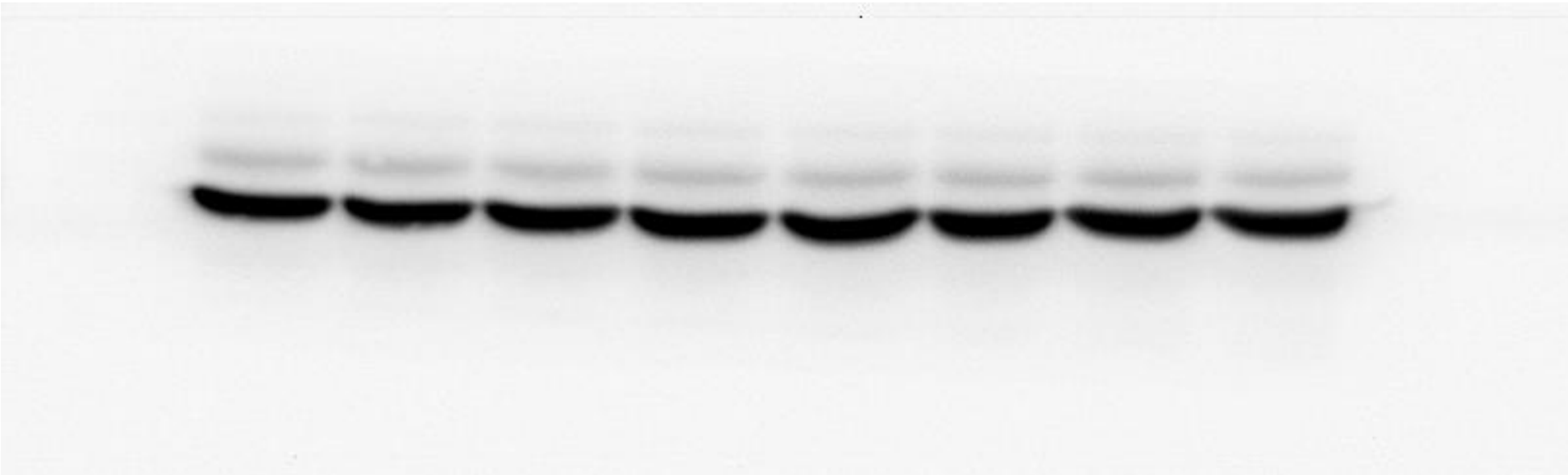

Figure 3H-β-actin-Repeat 2

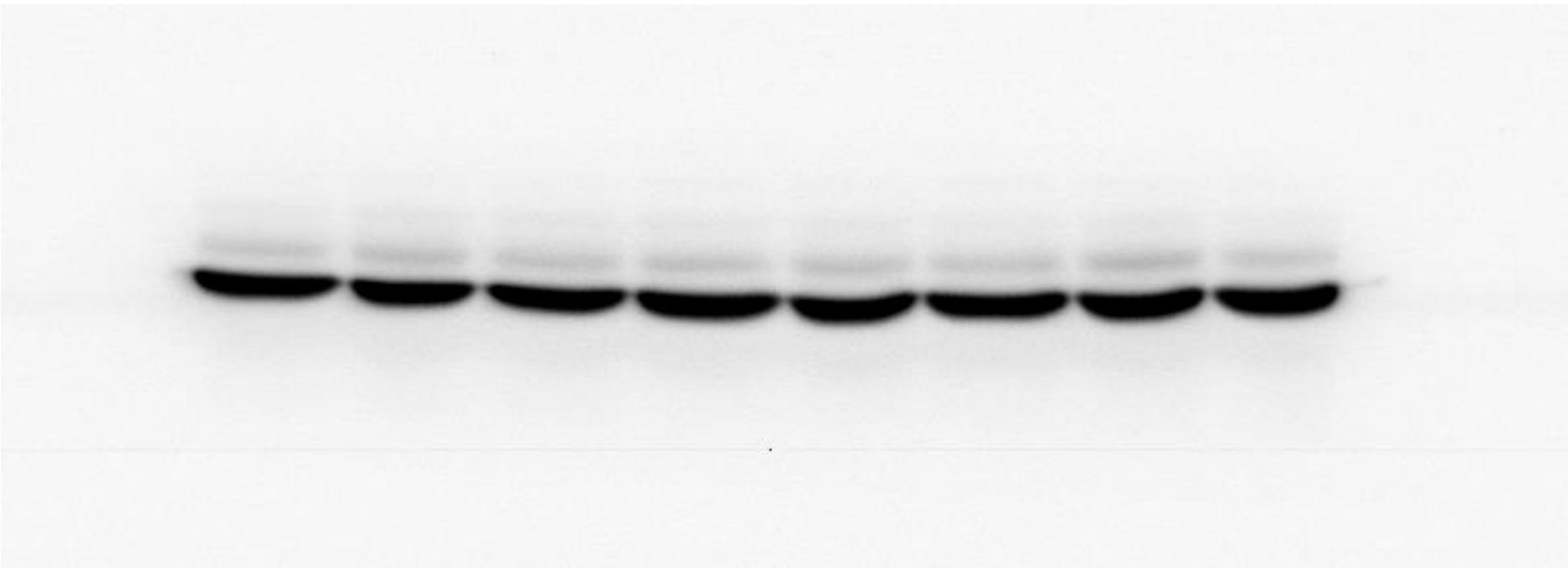

Figure 3H-β-actin-Repeat 3

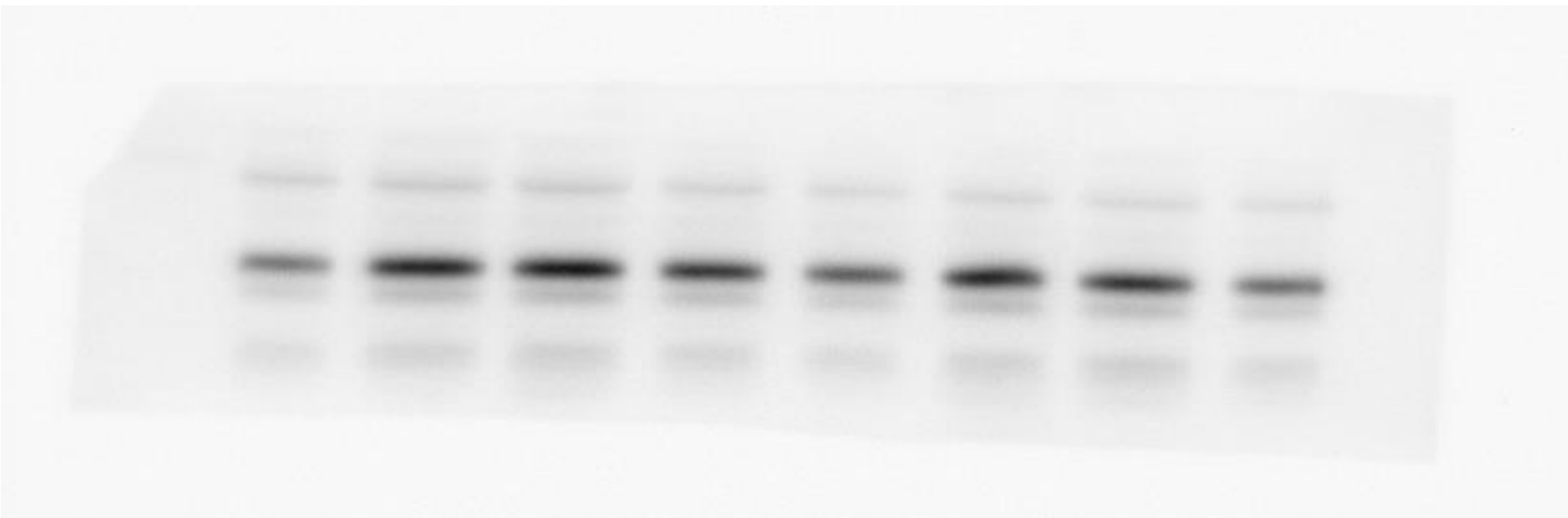

Figure 4A-TWEAK-Repeat 1

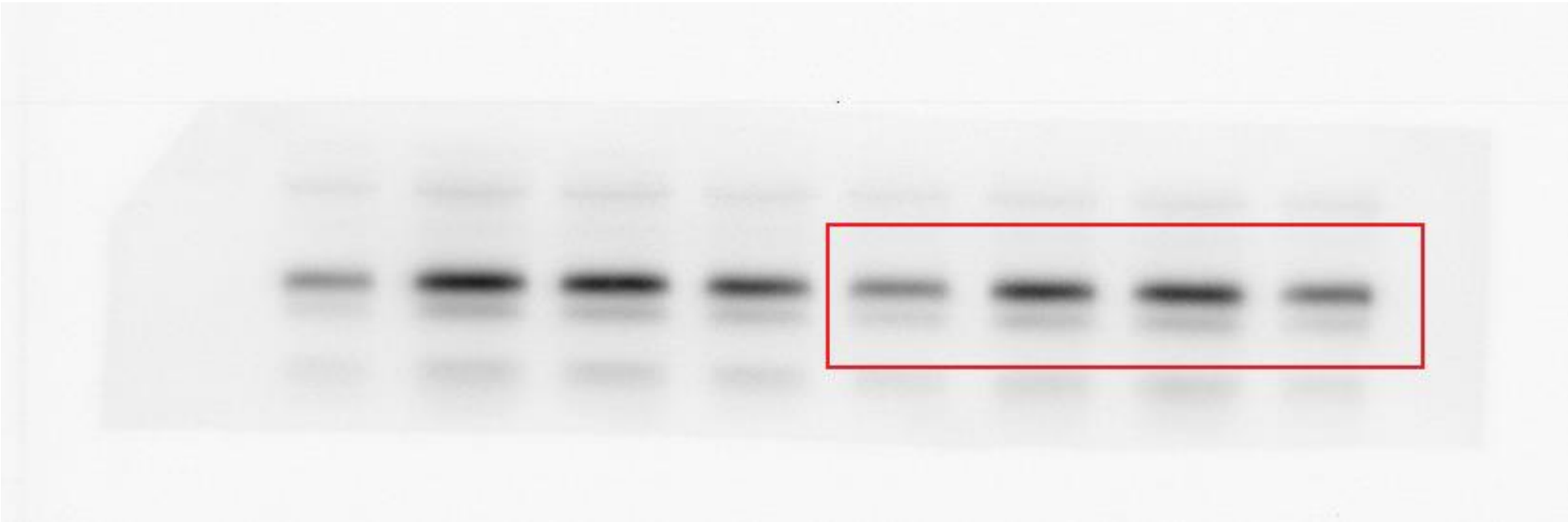

Figure 4A-TWEAK-Repeat 2

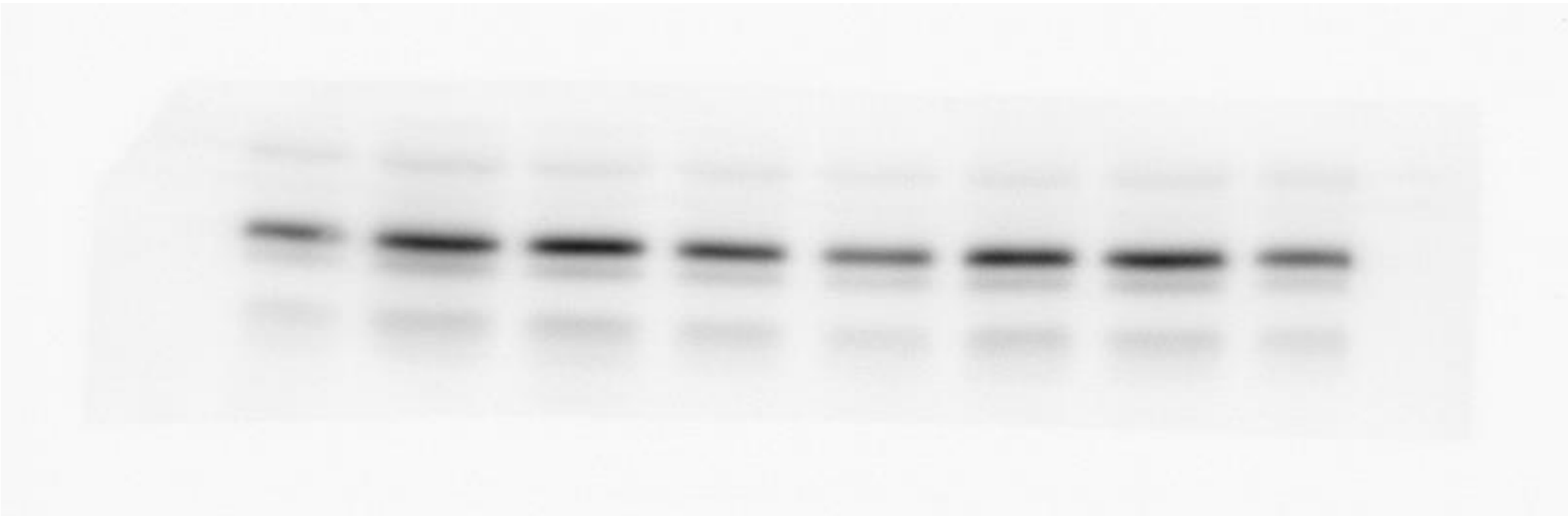

Figure 4A-TWEAK-Repeat 3

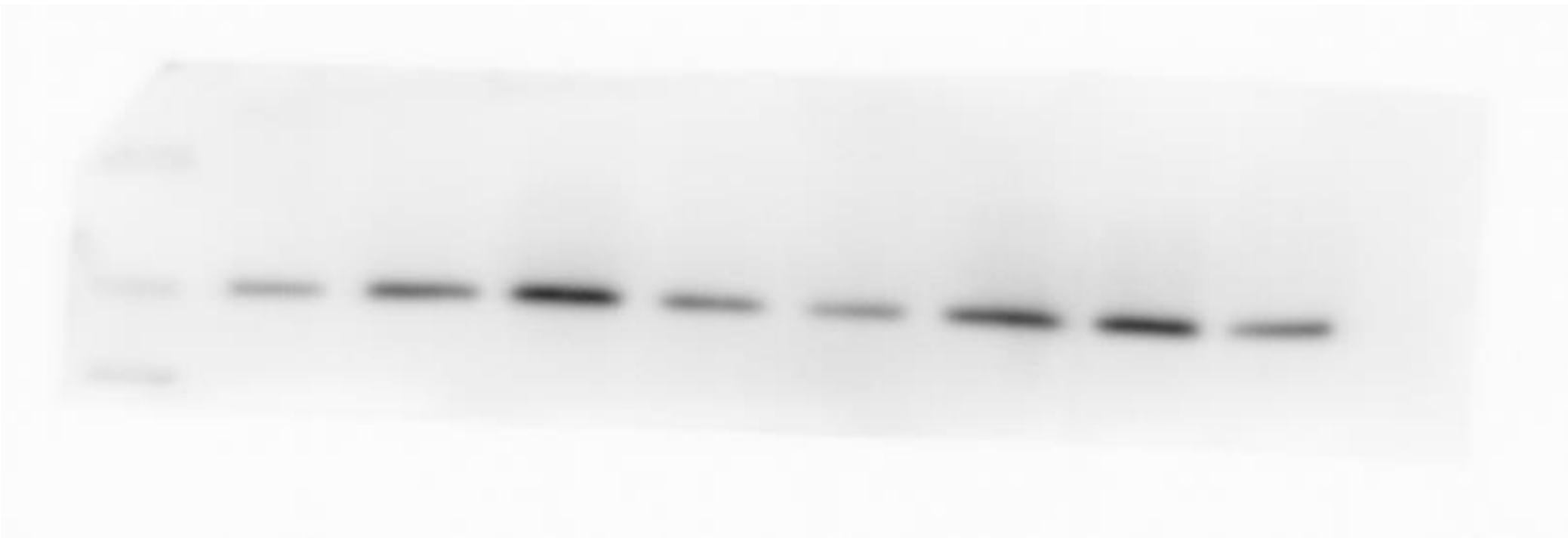

Figure 4A-Fn14-Repeat 1

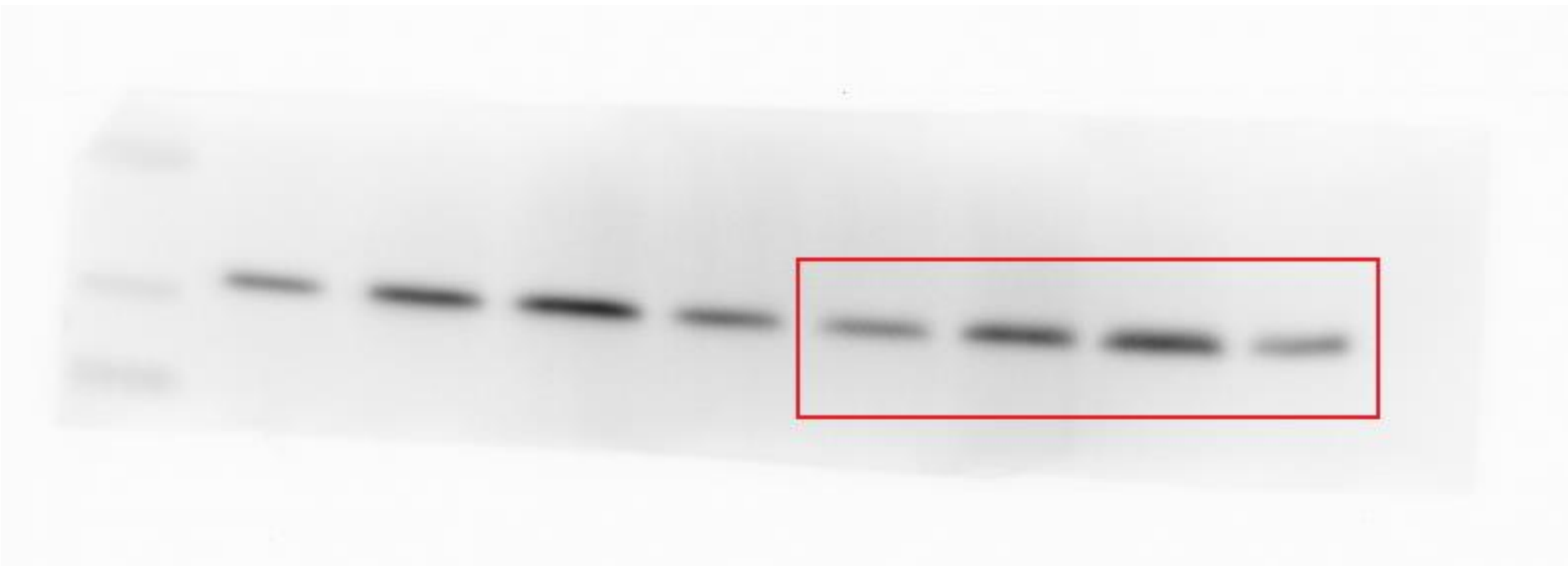

Figure 4A-Fn14-Repeat 2

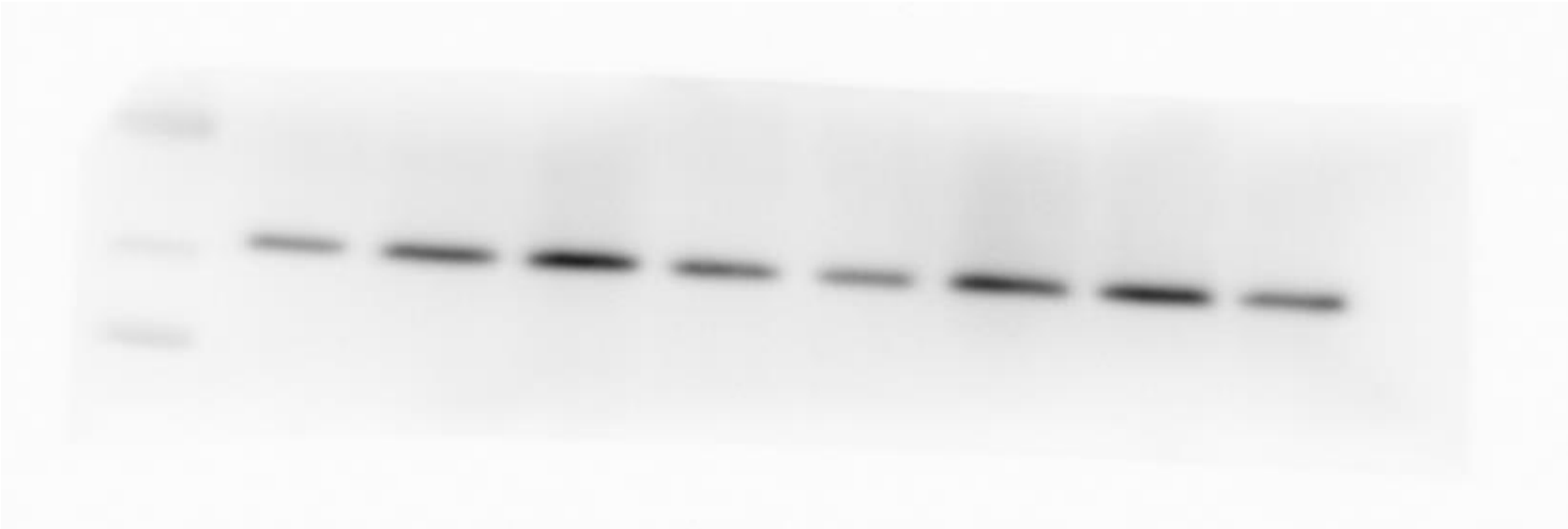

Figure 4A-Fn14-Repeat 3

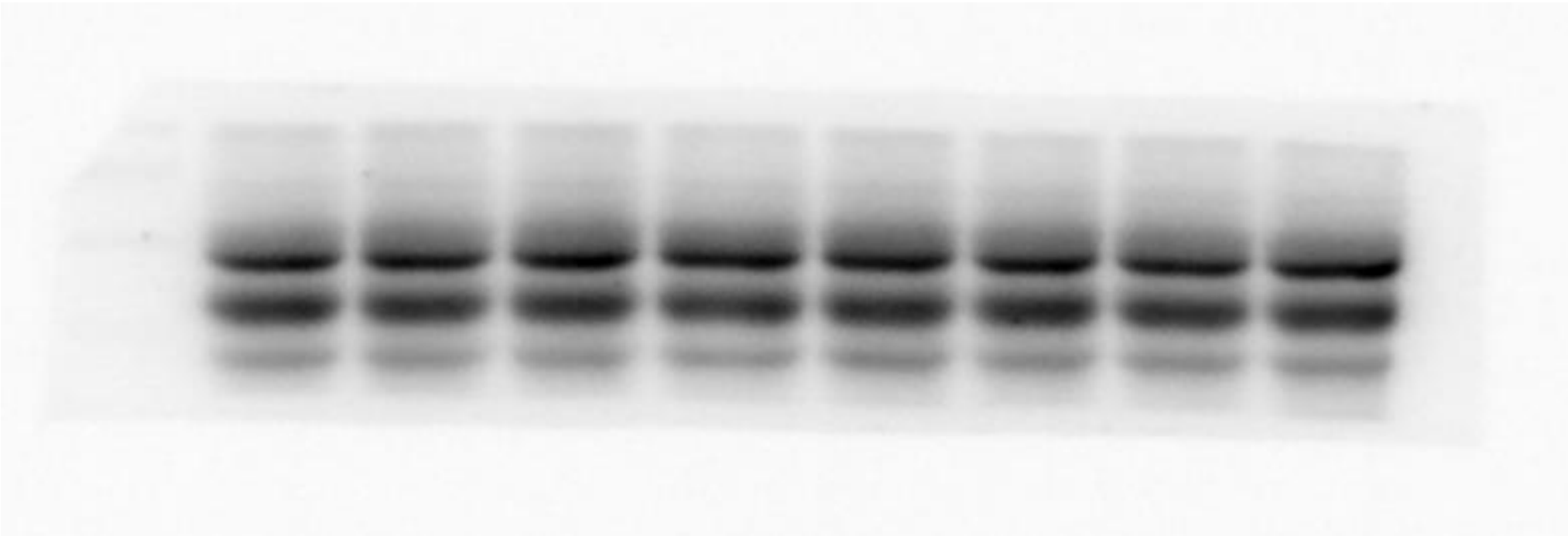

Figure 4A-AMPK-Repeat 1

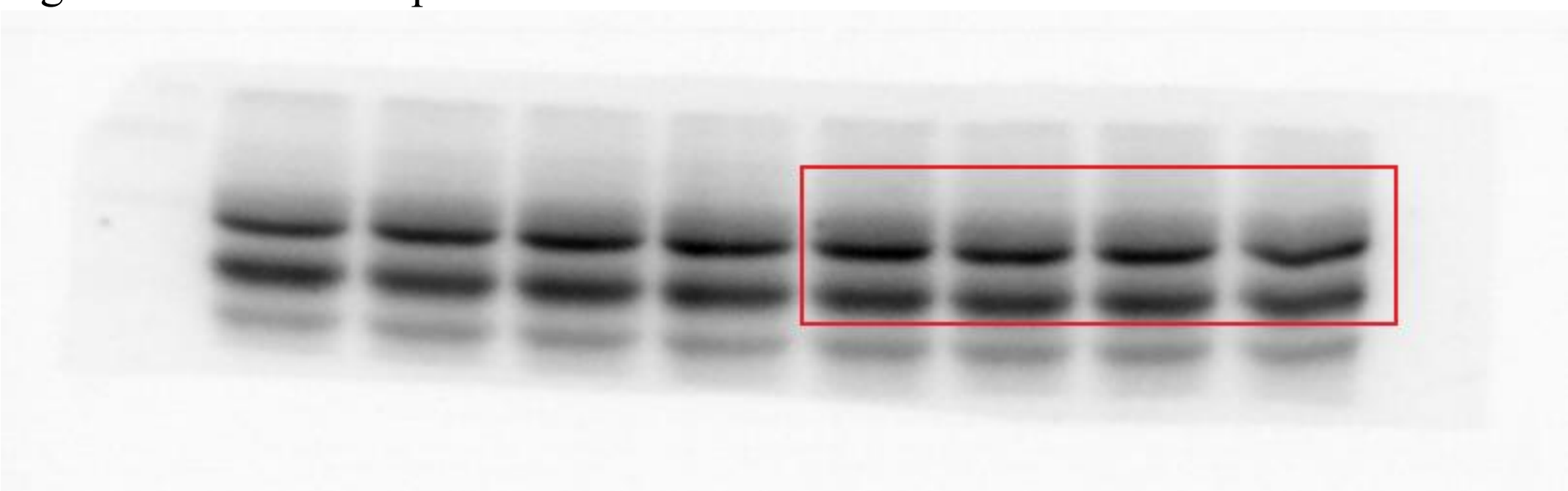

Figure 4A-AMPK-Repeat 2

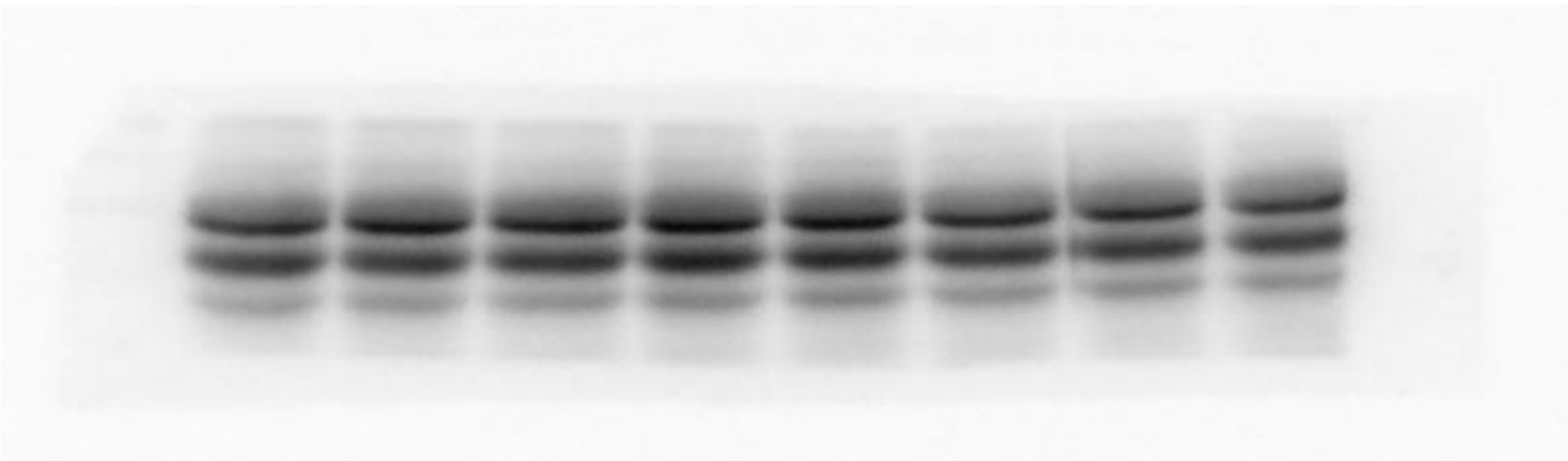

Figure 4A-AMPK-Repeat 3

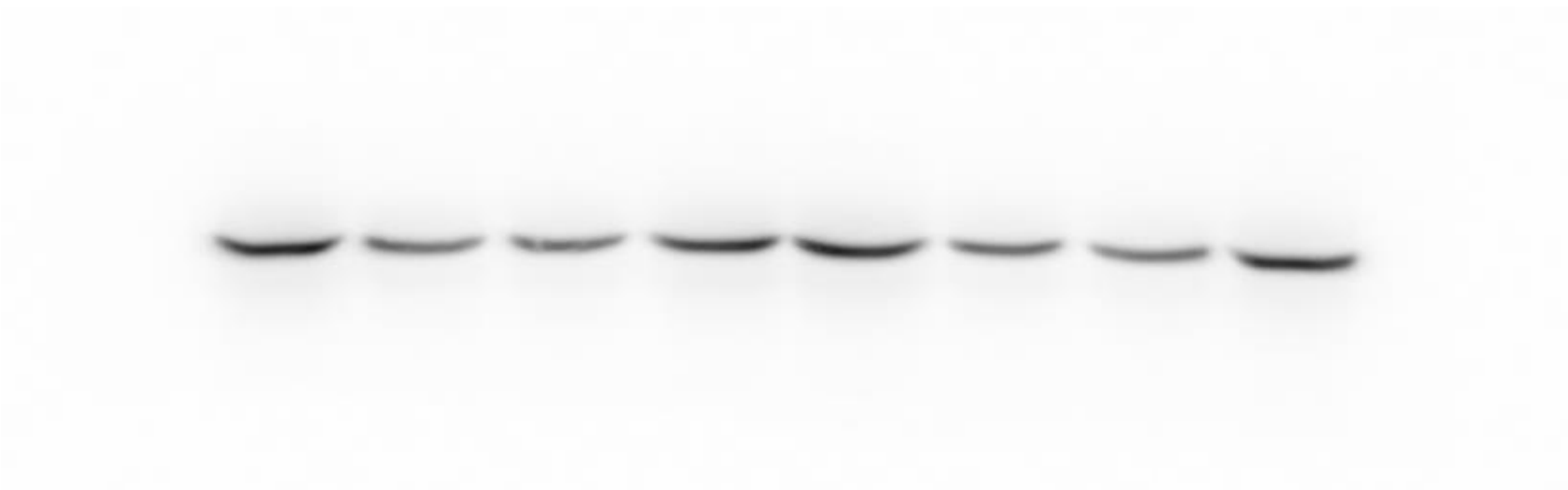

Figure 4A-p-AMPK-Repeat 1

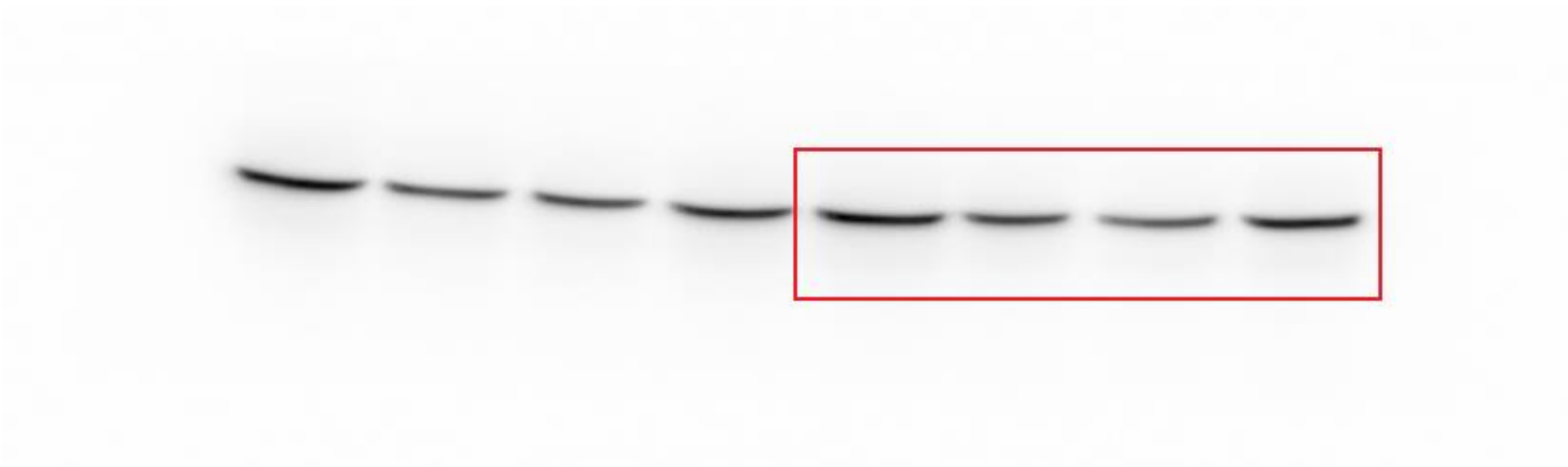

Figure 4A-p-AMPK-Repeat 2

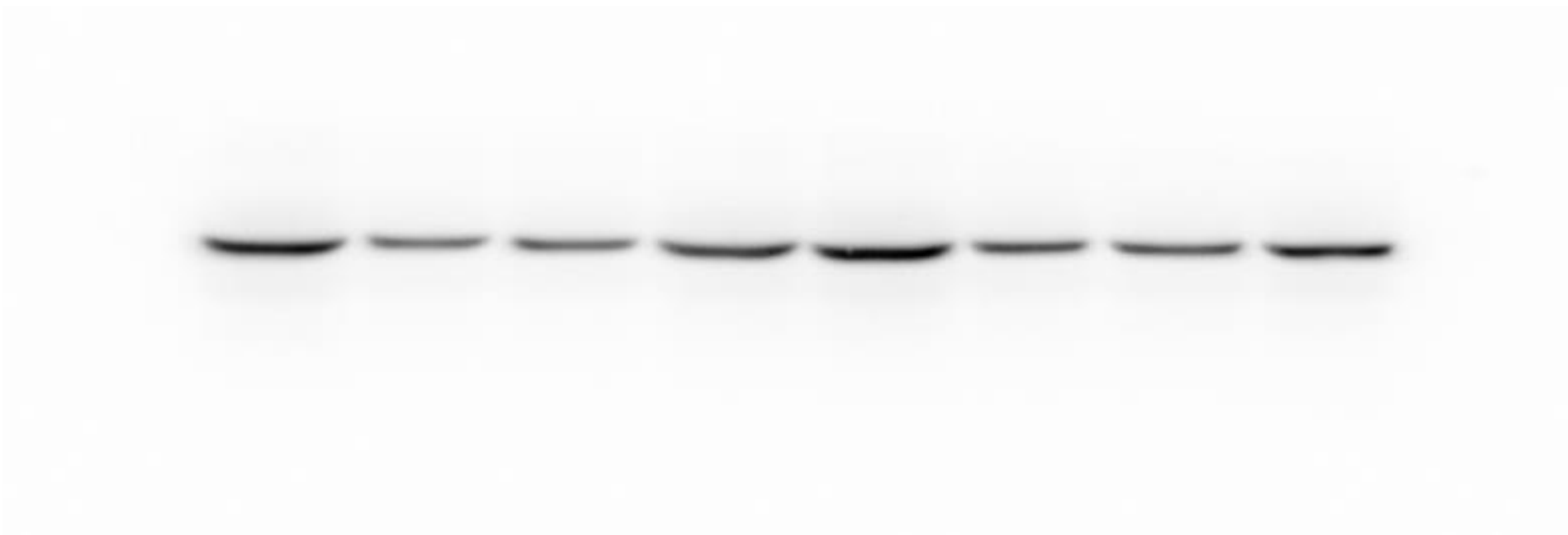

Figure 4A-p-AMPK-Repeat 3

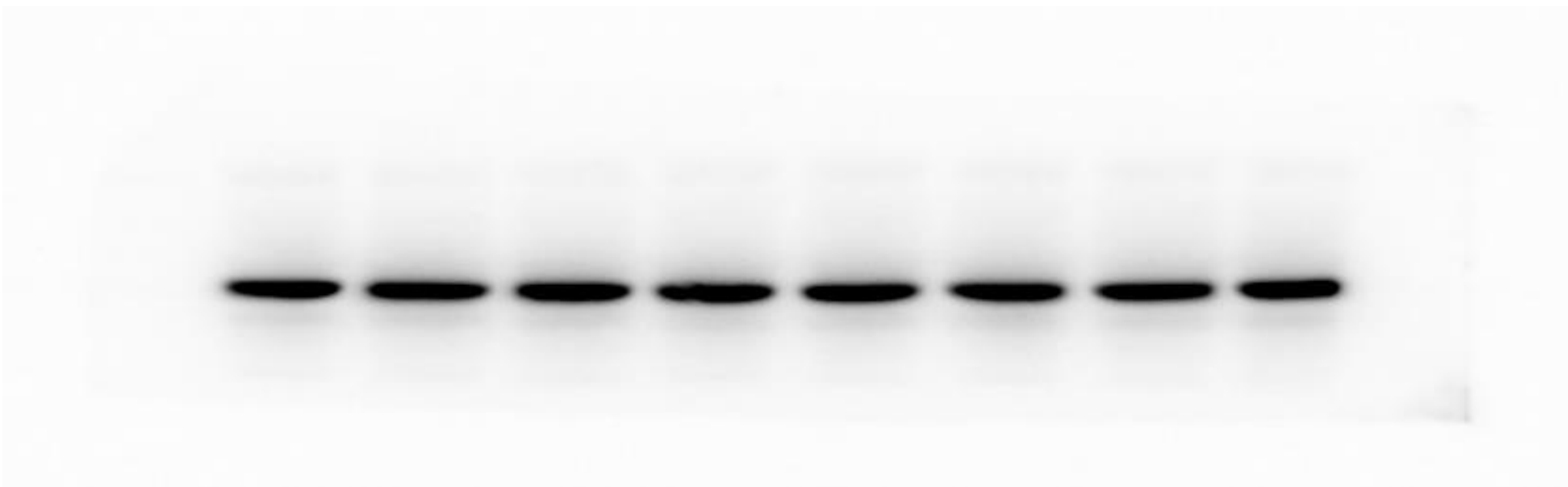

Figure 4A-p38-Repeat 1

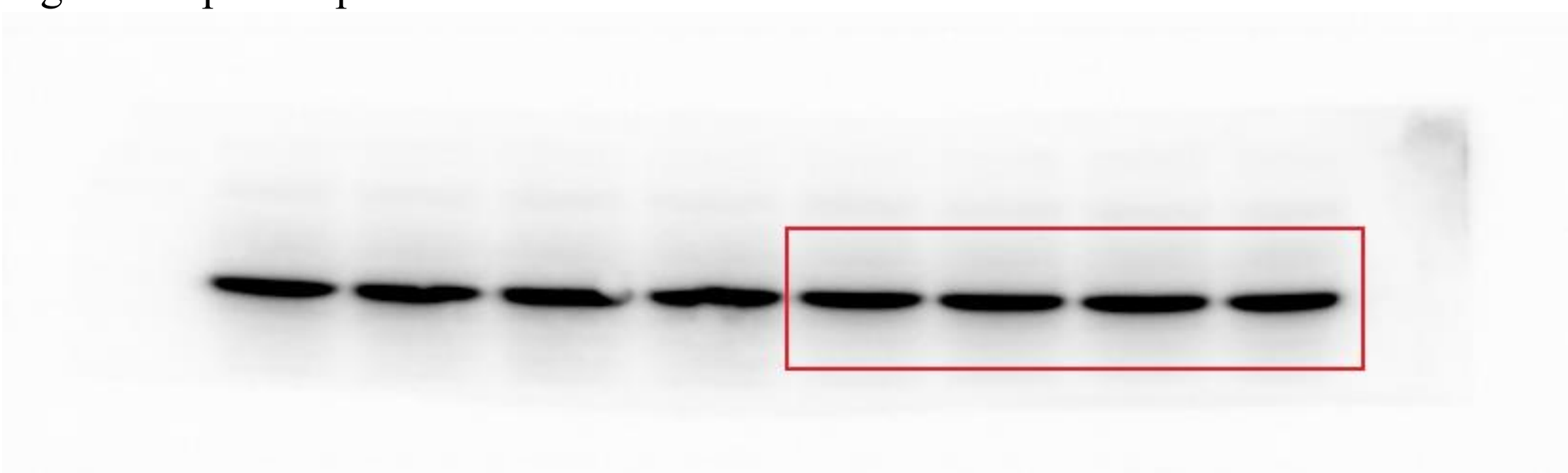

Figure 4A-p38-Repeat 2

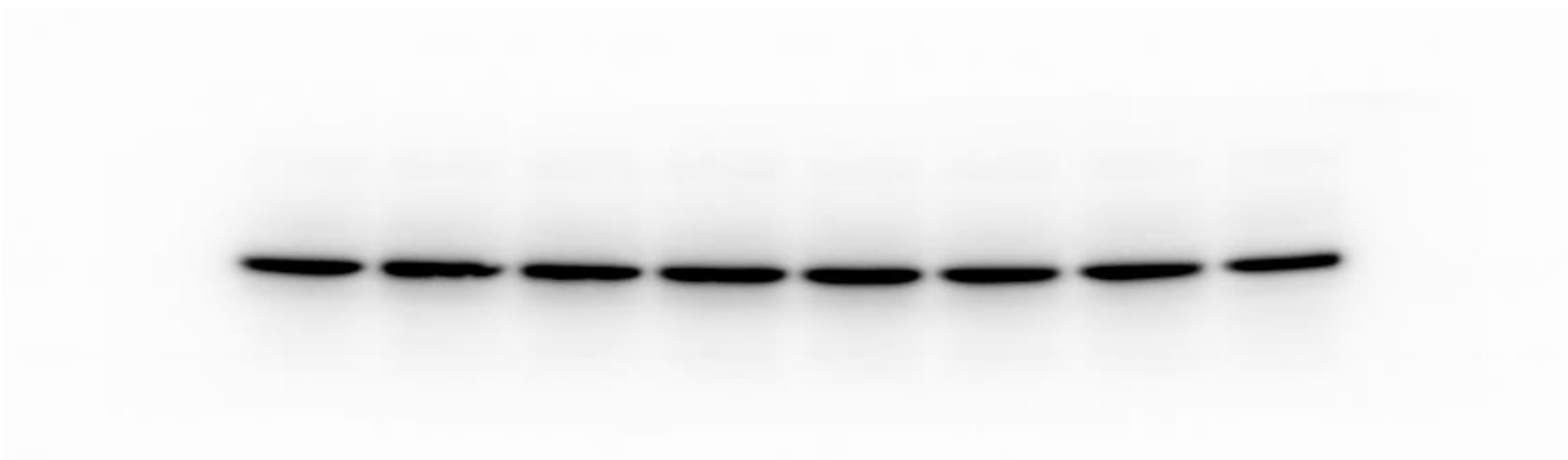

Figure 4A-p38-Repeat 3

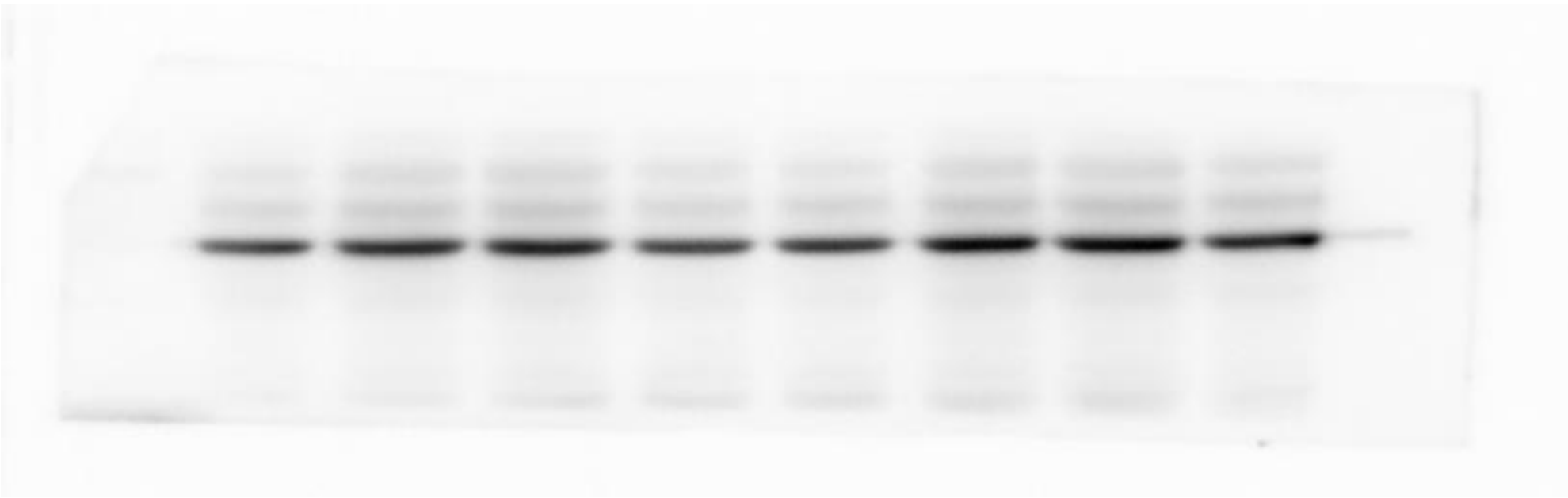

Figure 4A-p-p38-Repeat 1

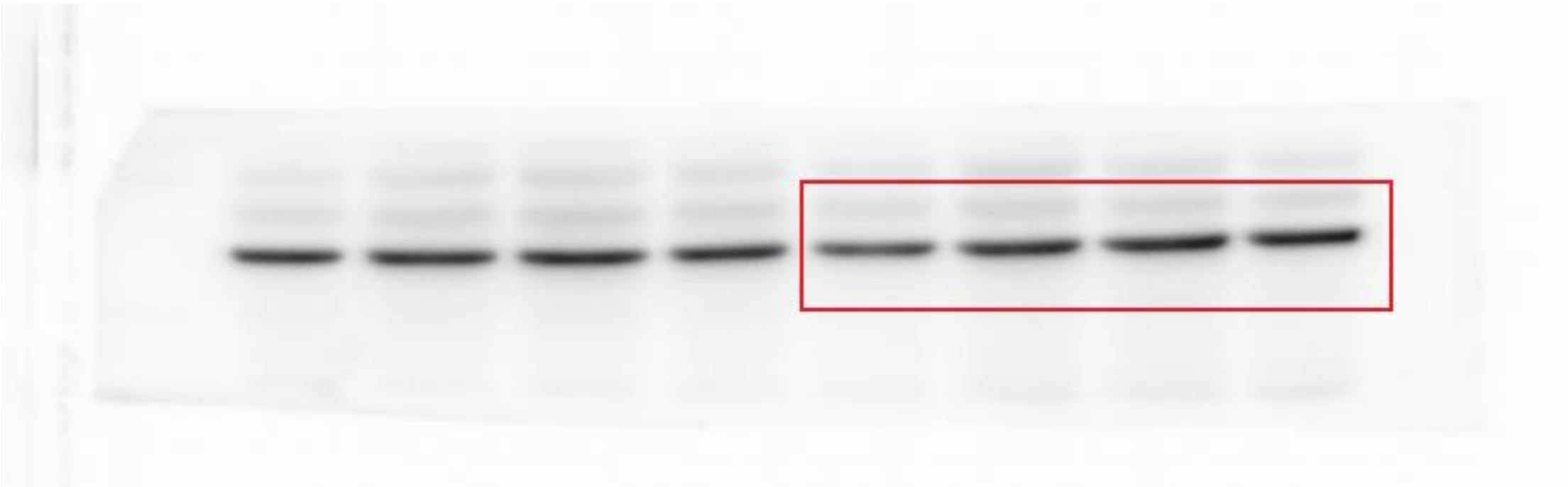

Figure 4A-p-p38-Repeat 2

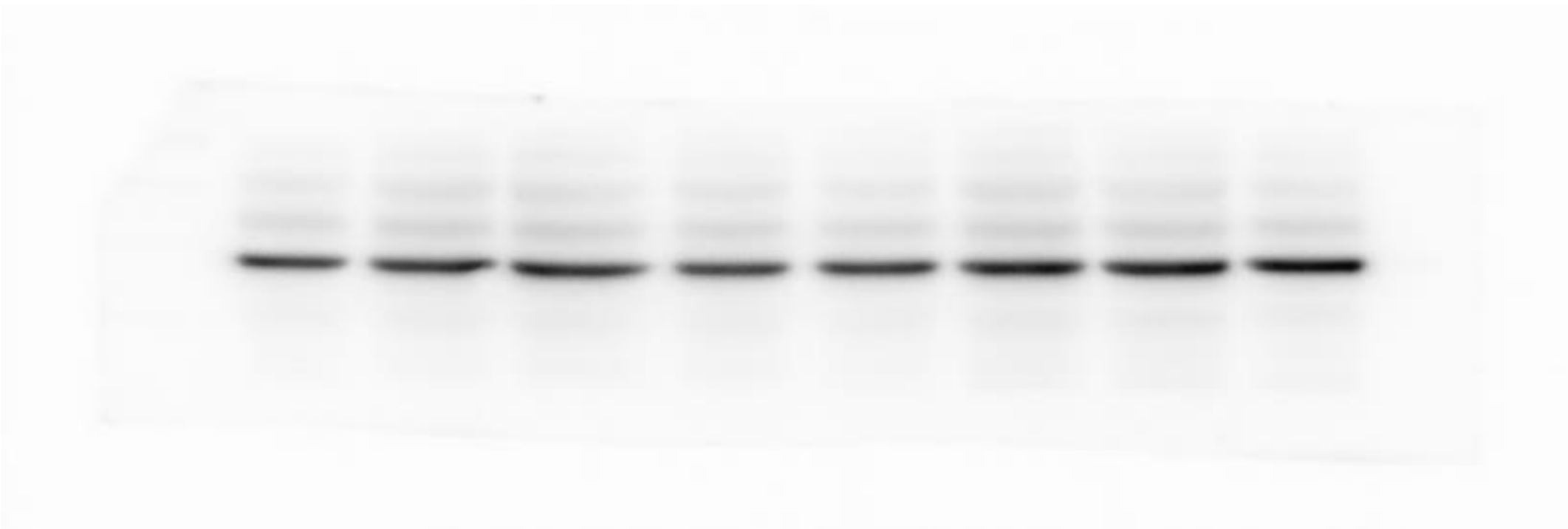

Figure 4A-p-p38-Repeat 3

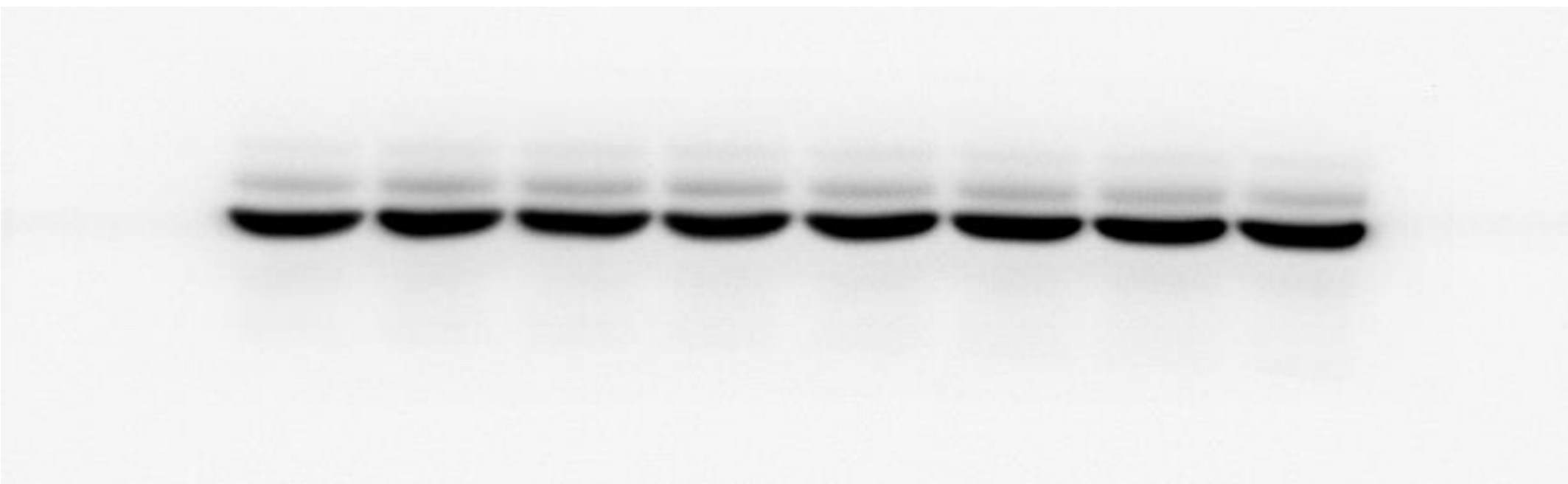

Figure 4A-β-actin-Repeat 1

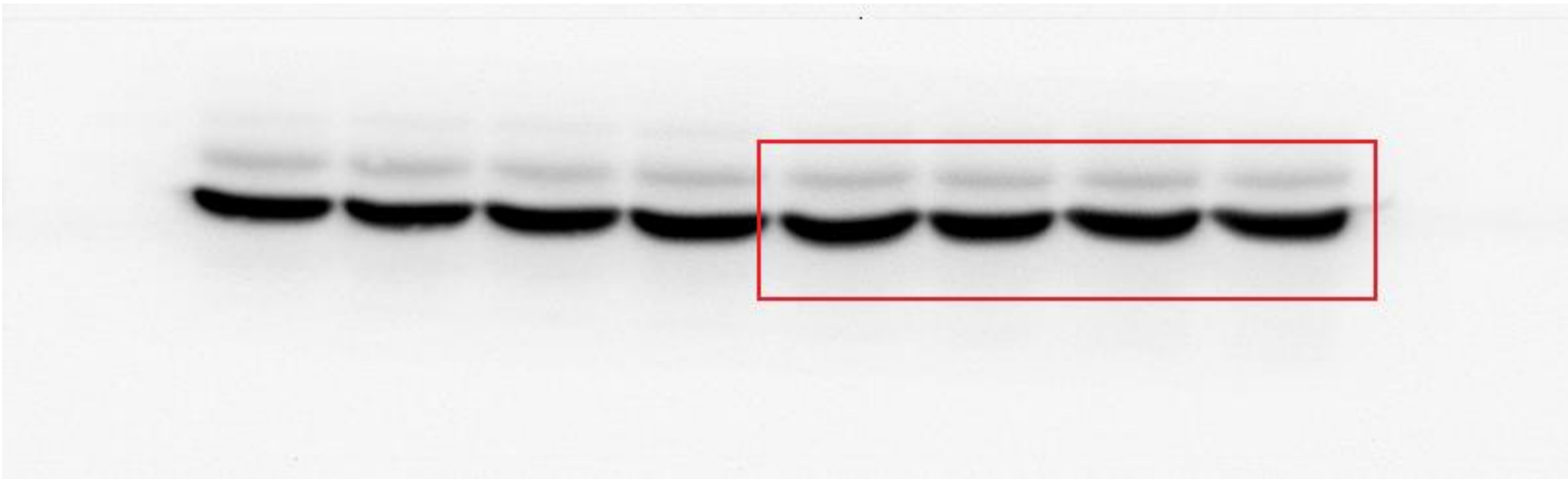

Figure 4A-β-actin-Repeat 2

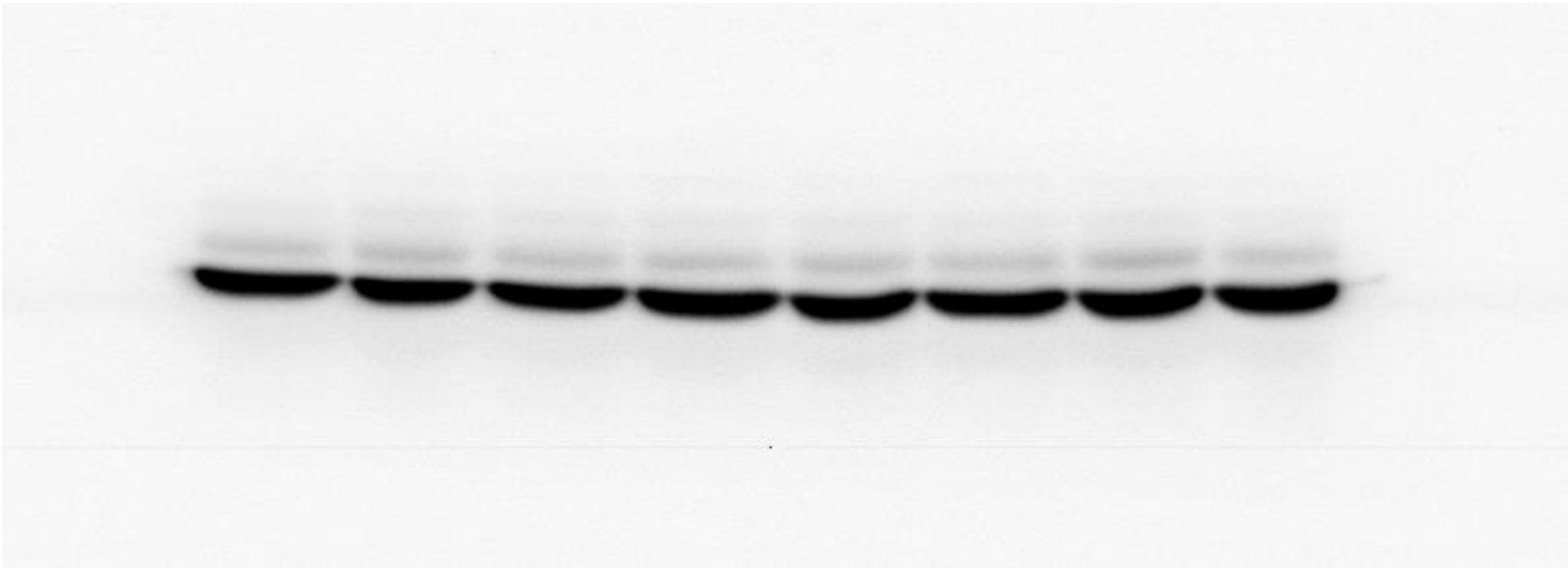

Figure 4A-β-actin-Repeat 3

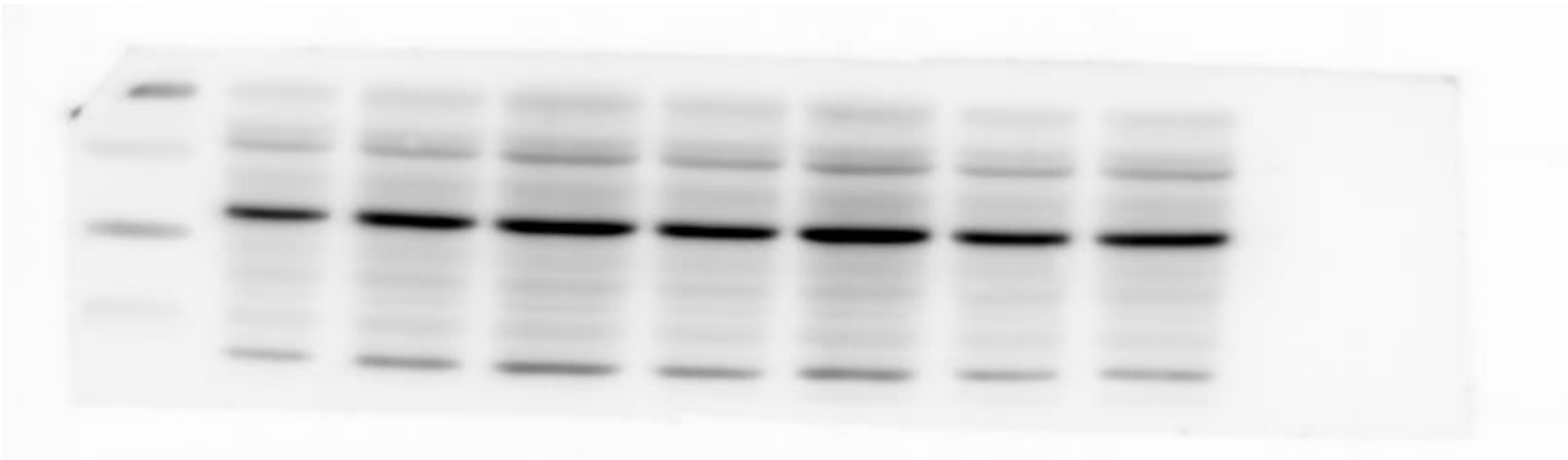

Figure 6C-TWEAK Repeat 1

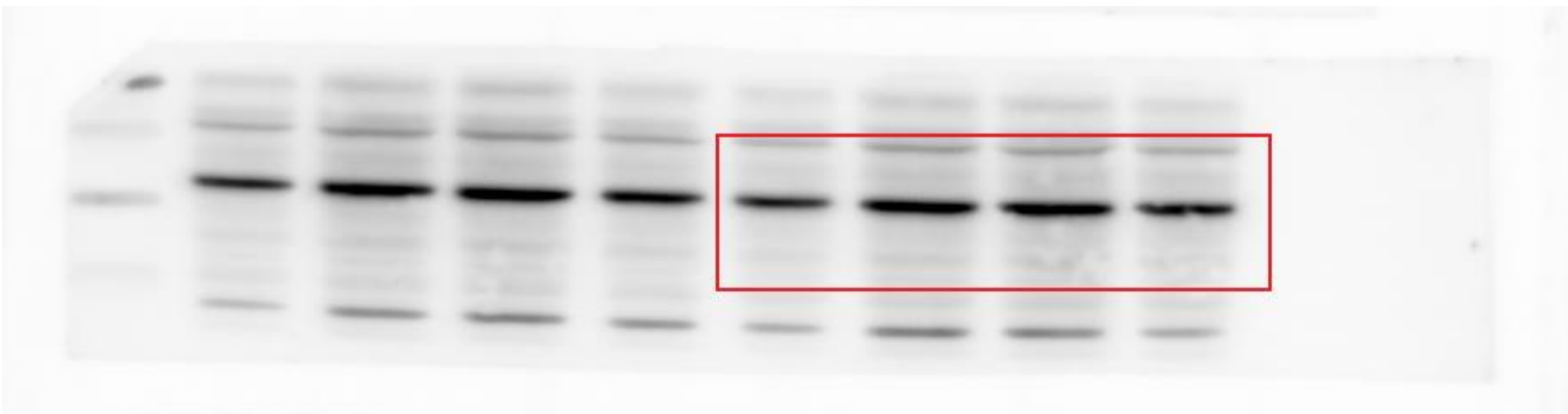

Figure 6C-TWEAK Repeat 2 and 3

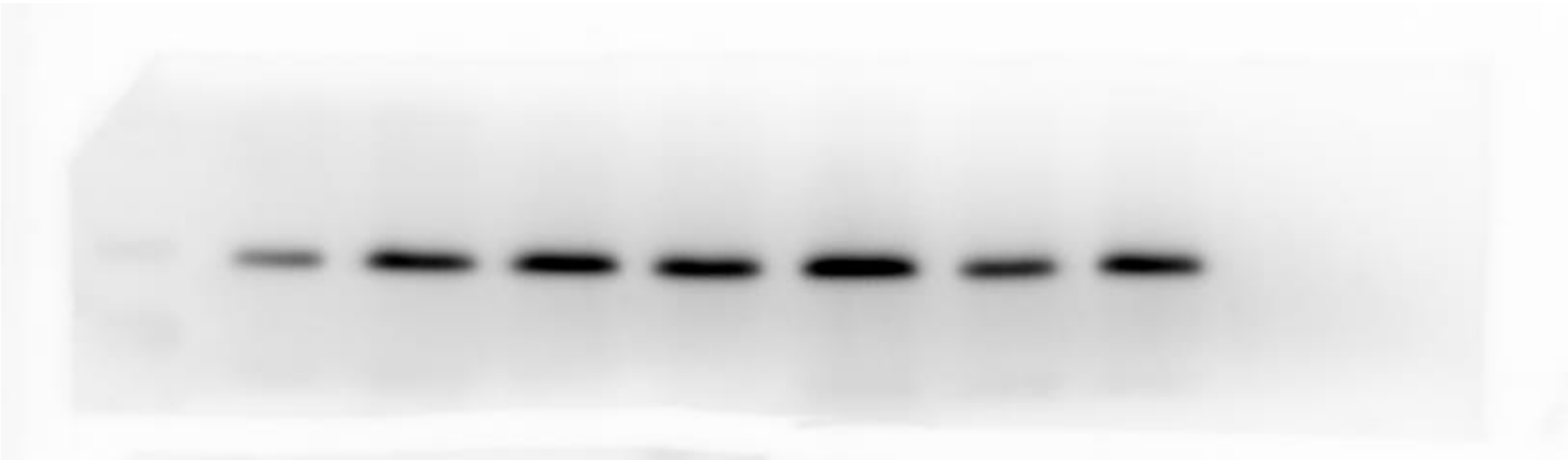

Figure 6C-Fn14 Repeat 1

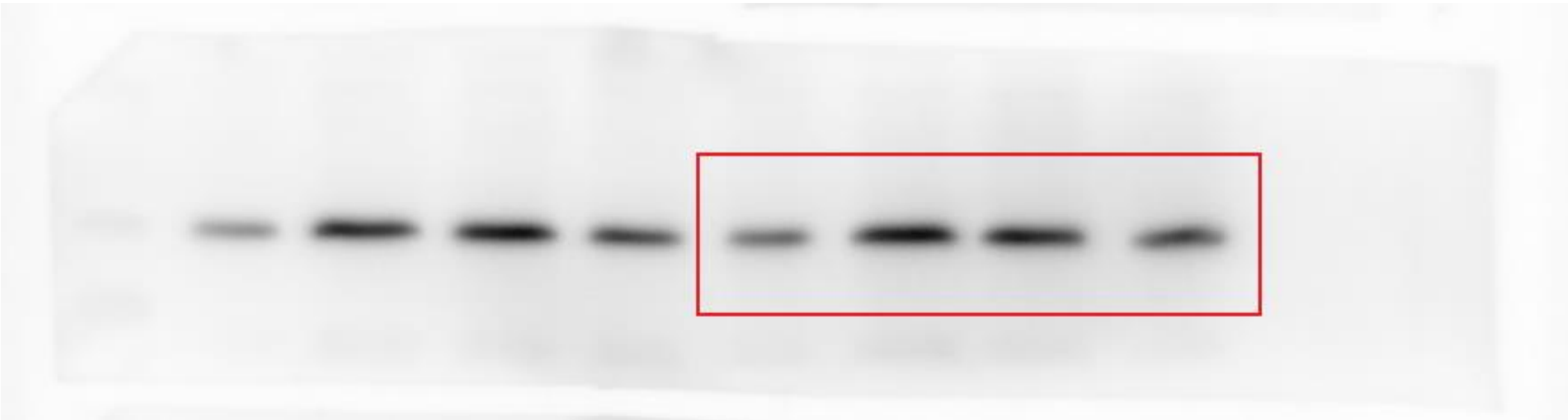

Figure 6C-Fn14 Repeat 2 and 3

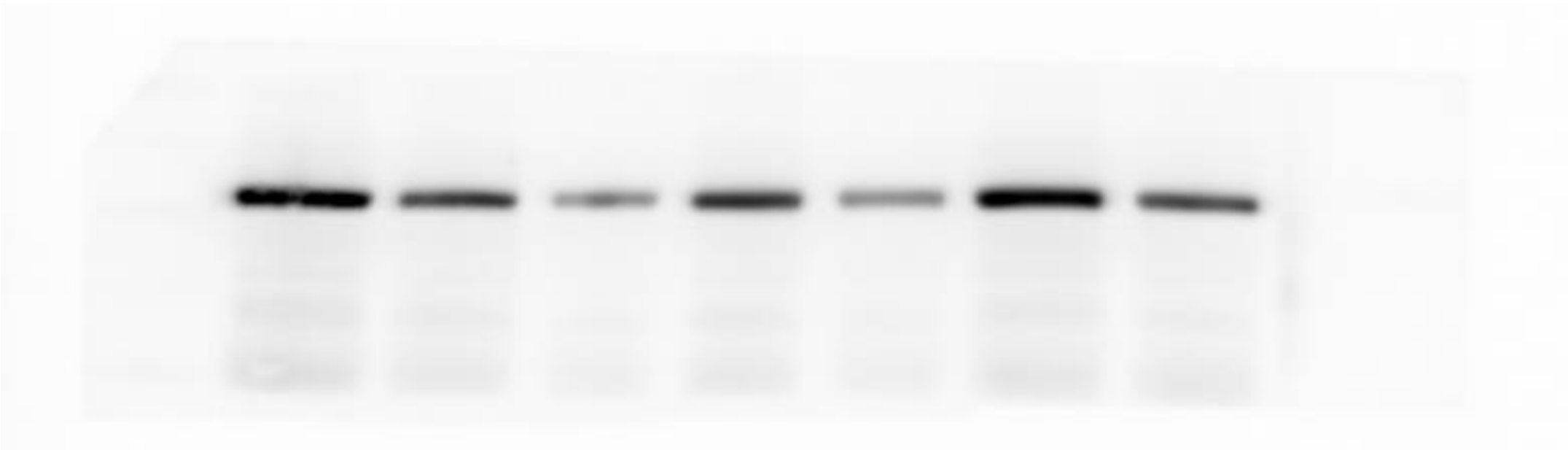

Figure 6C-SIRT1 Repeat 1

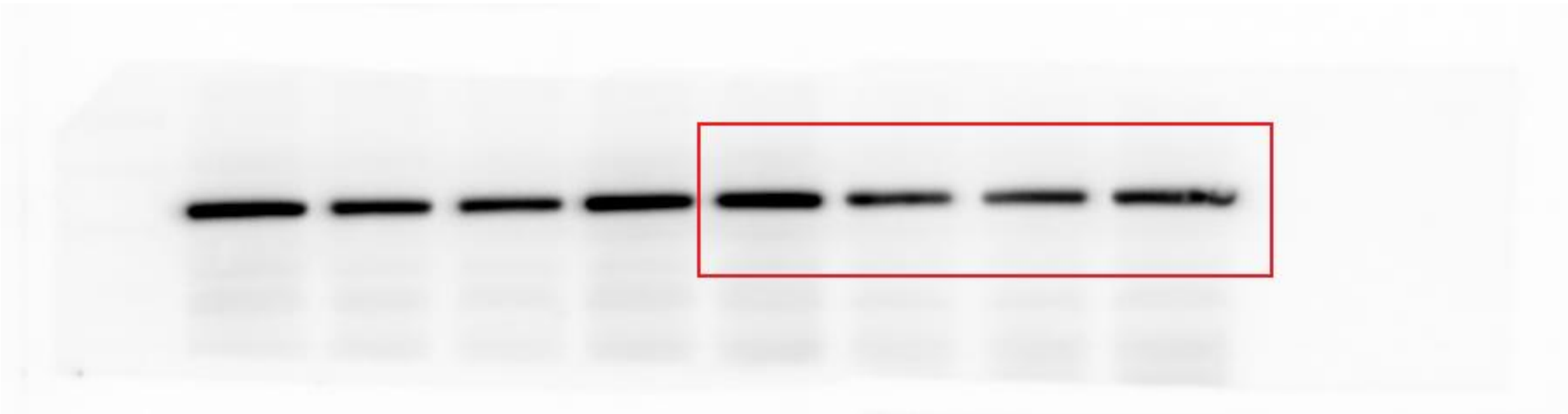

Figure 6C-SIRT1 Repeat 2 and 3

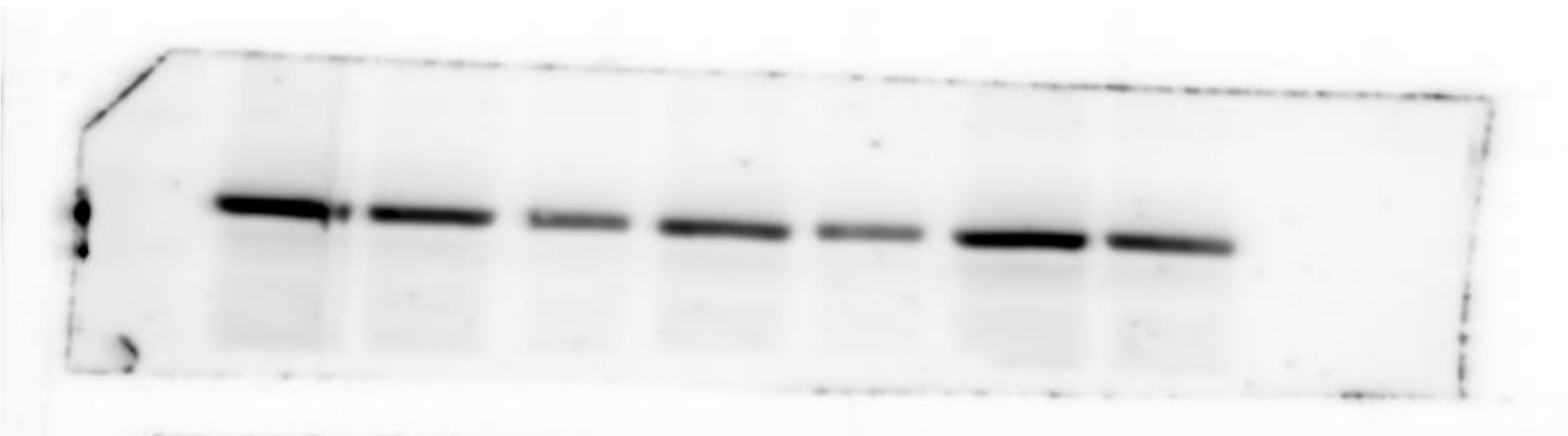

Figure 6C-PGC1-α -1 Repeat 1

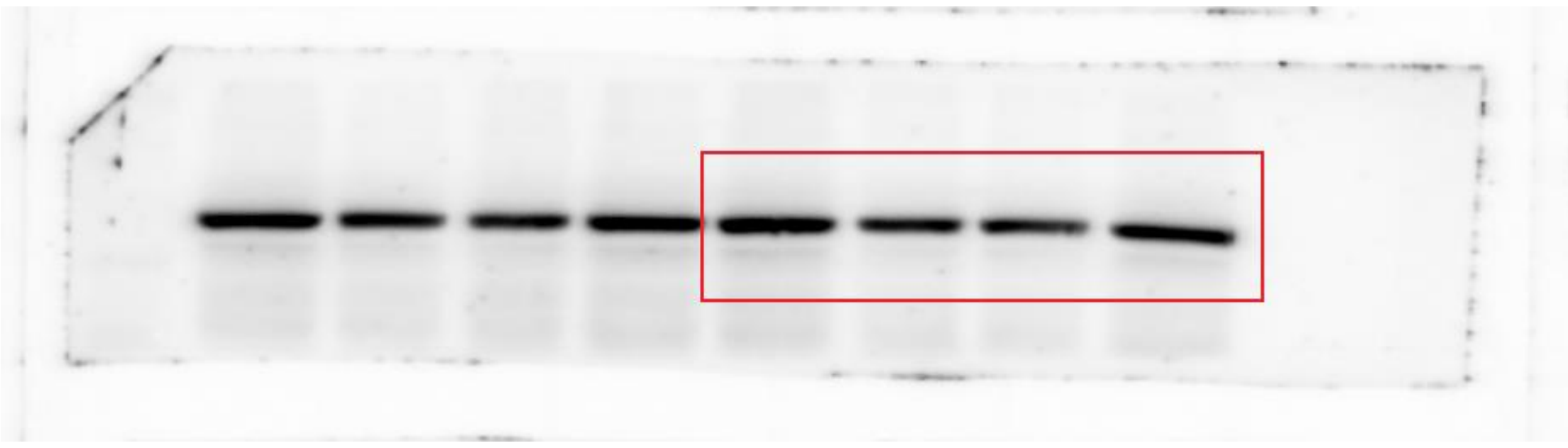

Figure 6C-PGC1-α -1 Repeat 2 and 3

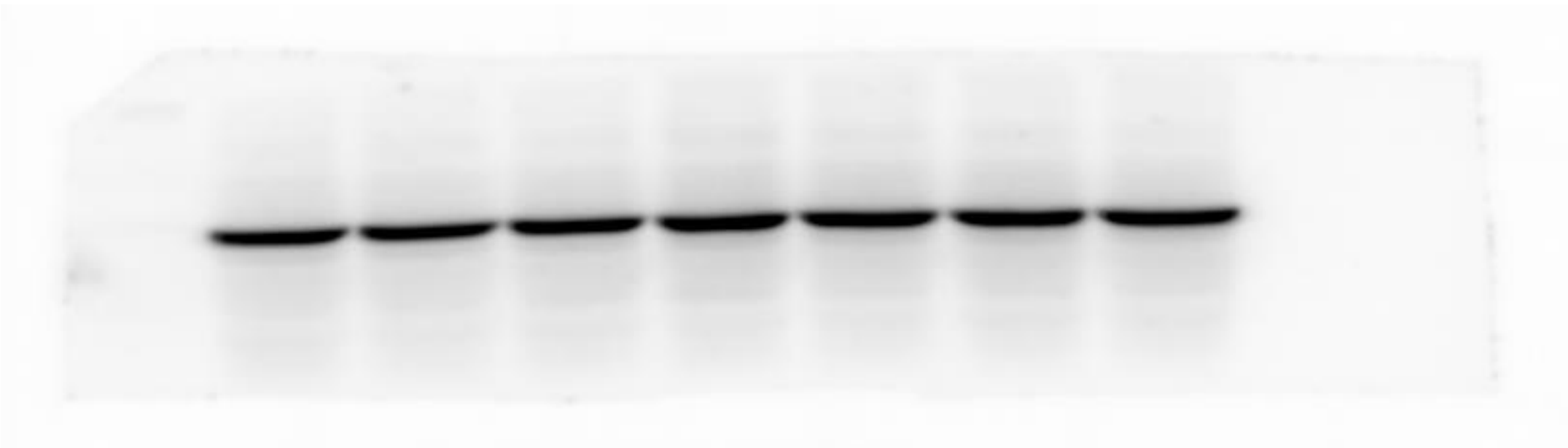

Figure 6C-AMPK Repeat 1

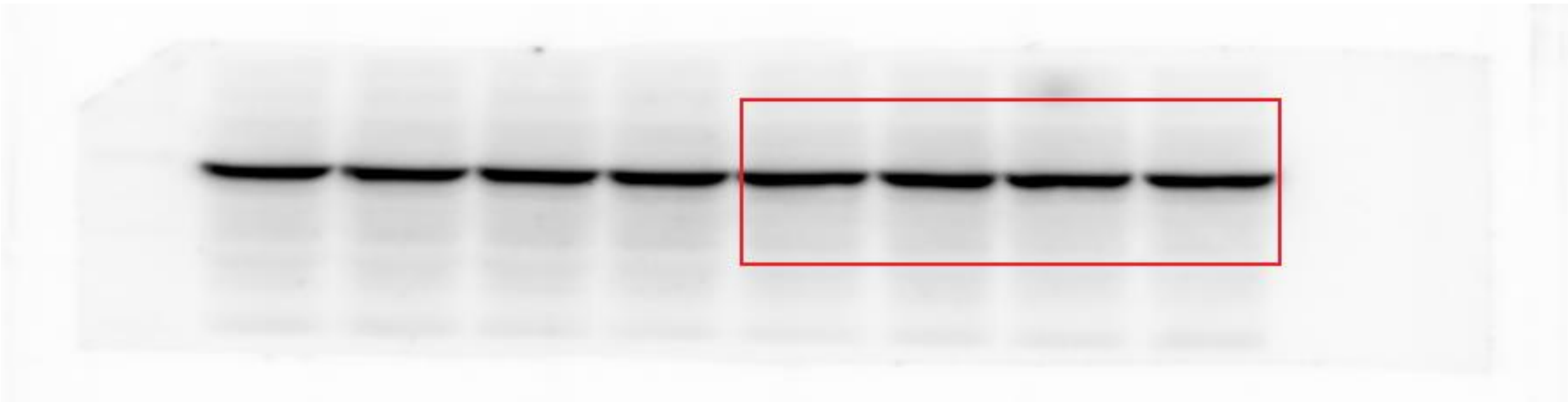

Figure 6C-AMPK Repeat 2 and 3

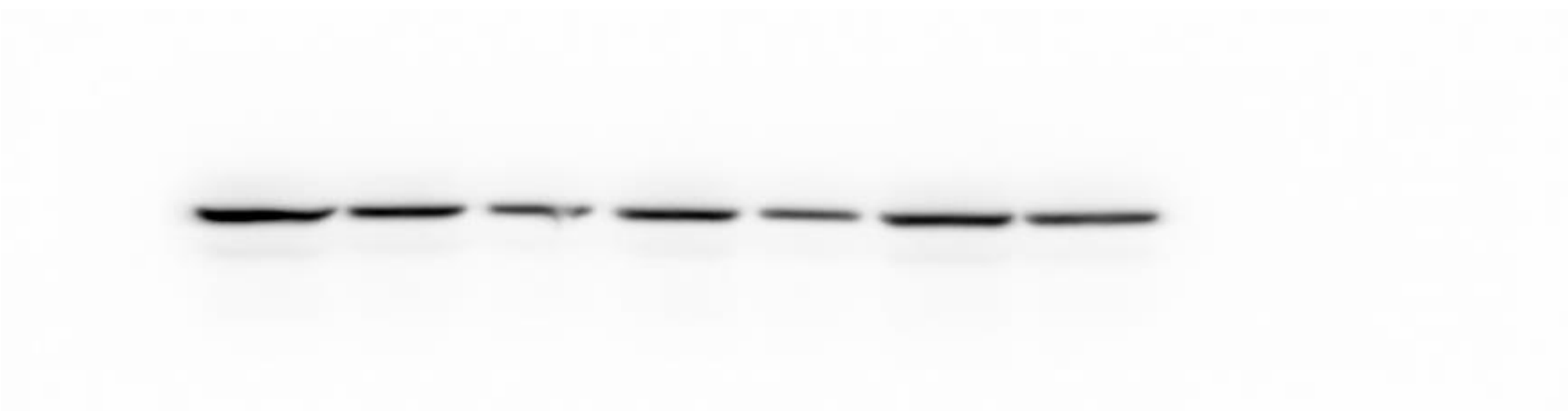

Figure 6C-p-AMPK Repeat 1

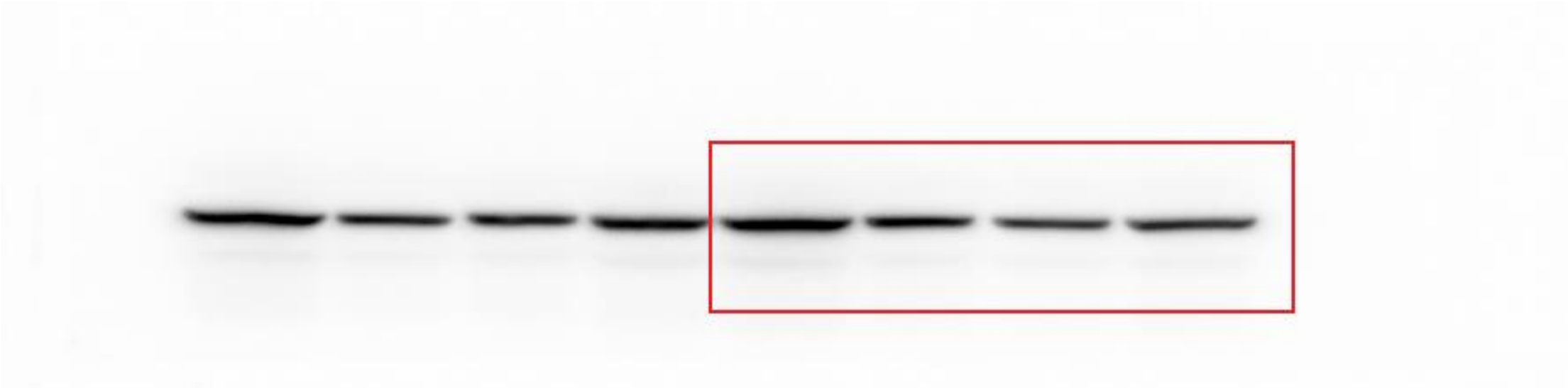

Figure 6C-p-AMPK Repeat 2 and 3

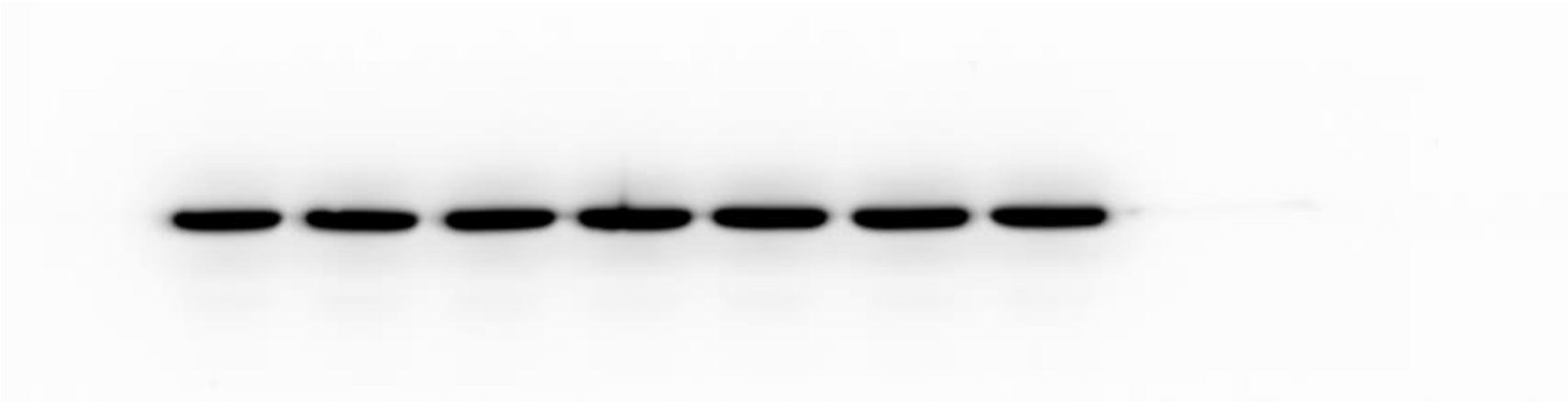

Figure 6C-p38 Repeat 1

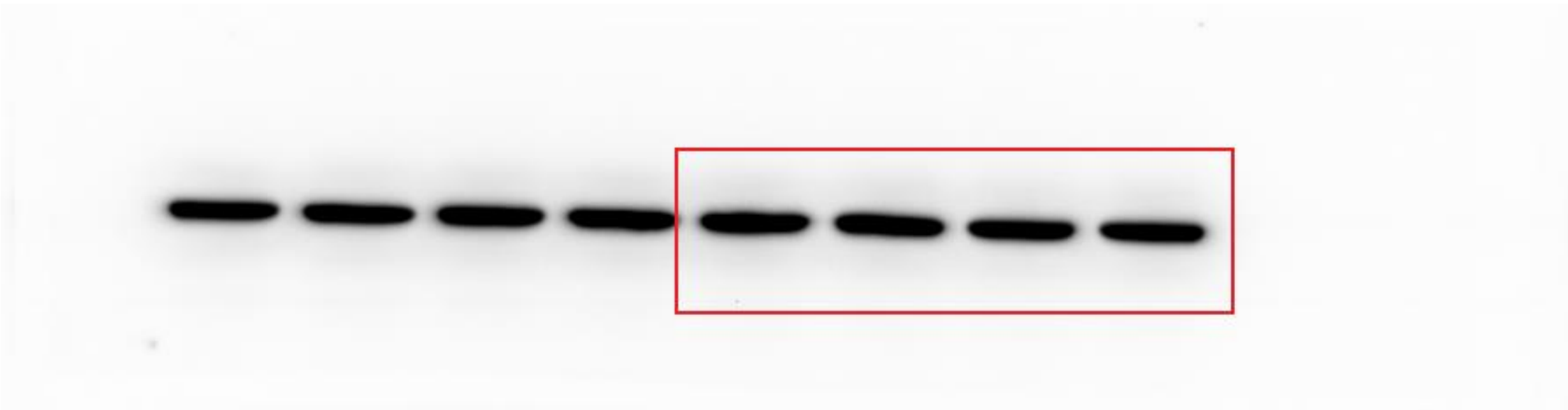

Figure 6C-p38 Repeat 2 and 3

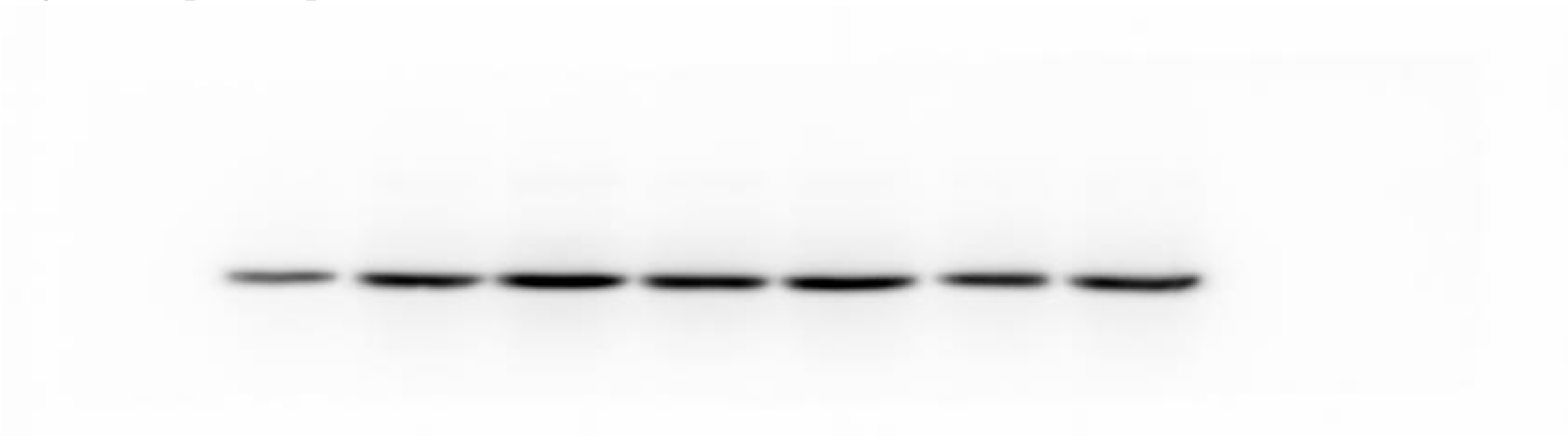

Figure 6C-p-p38 Repeat 1

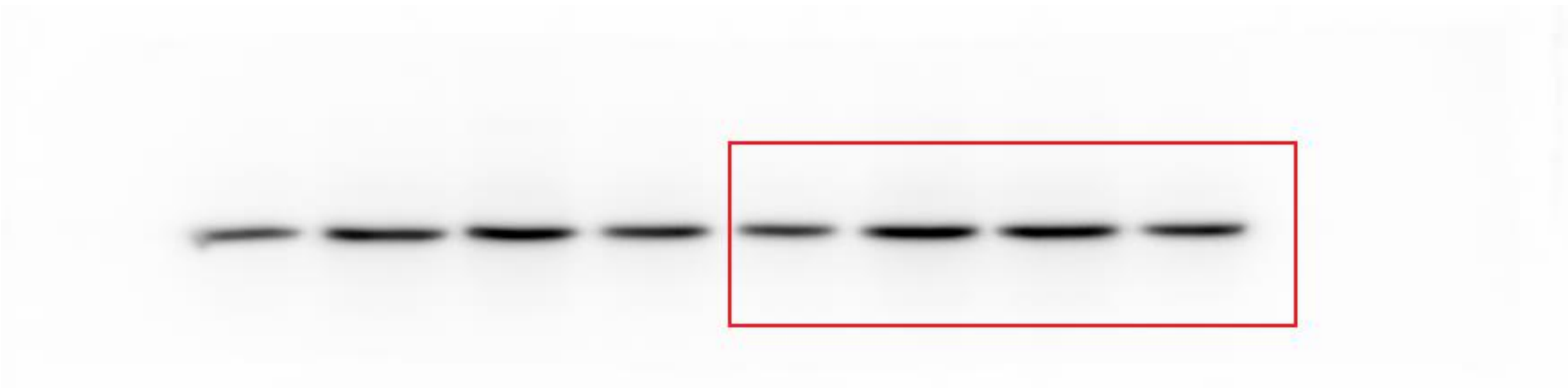

Figure 6C-p-p38 Repeat 2 and 3

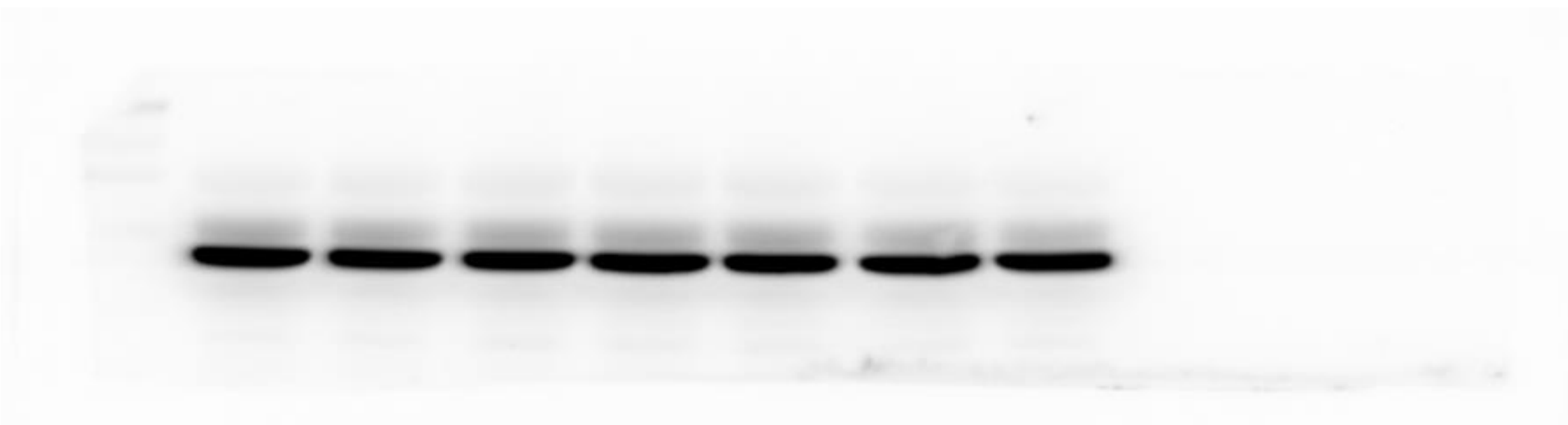

Figure 6C-β-actin Repeat 1

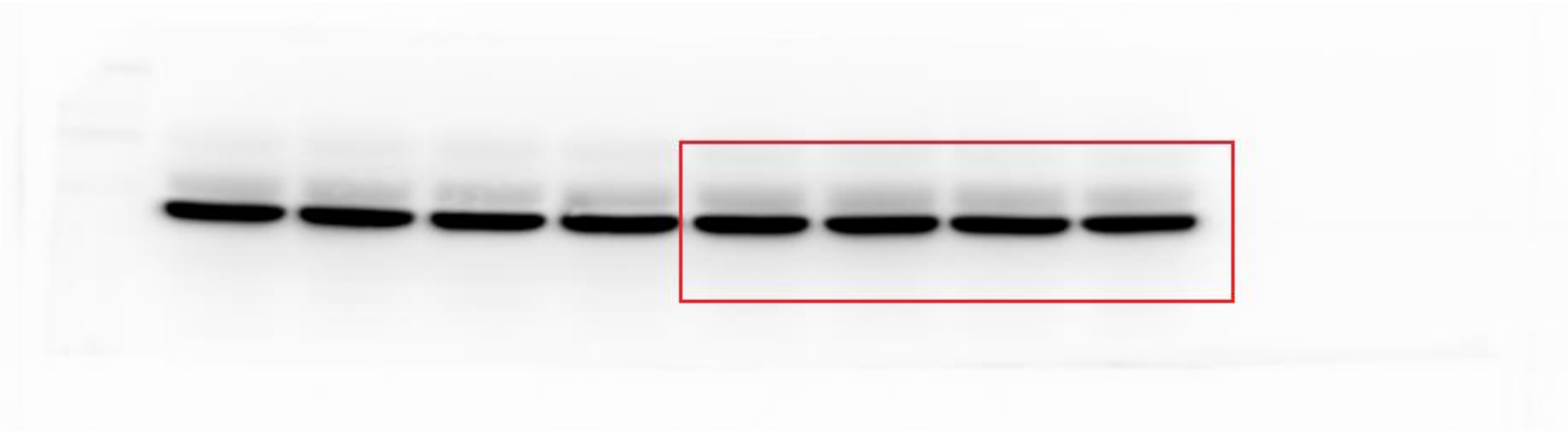

Figure 6C-β-actin Repeat 2 and 3
