# Supplementary material for: Targeting TWEAK to enhance mitochondrial biogenesis and attenuate age-related muscle mass loss
Source: Sci Rep. 2026 Aug 14;16:28221. doi: 10.1038/s41598-026-64401-2 (PMC13558719; doi:10.1038/s41598-026-64401-2)
Supplement: Supplementary file 2 [file 41598_2026_64401_MOESM2_ESM.pdf]

Figure 1F

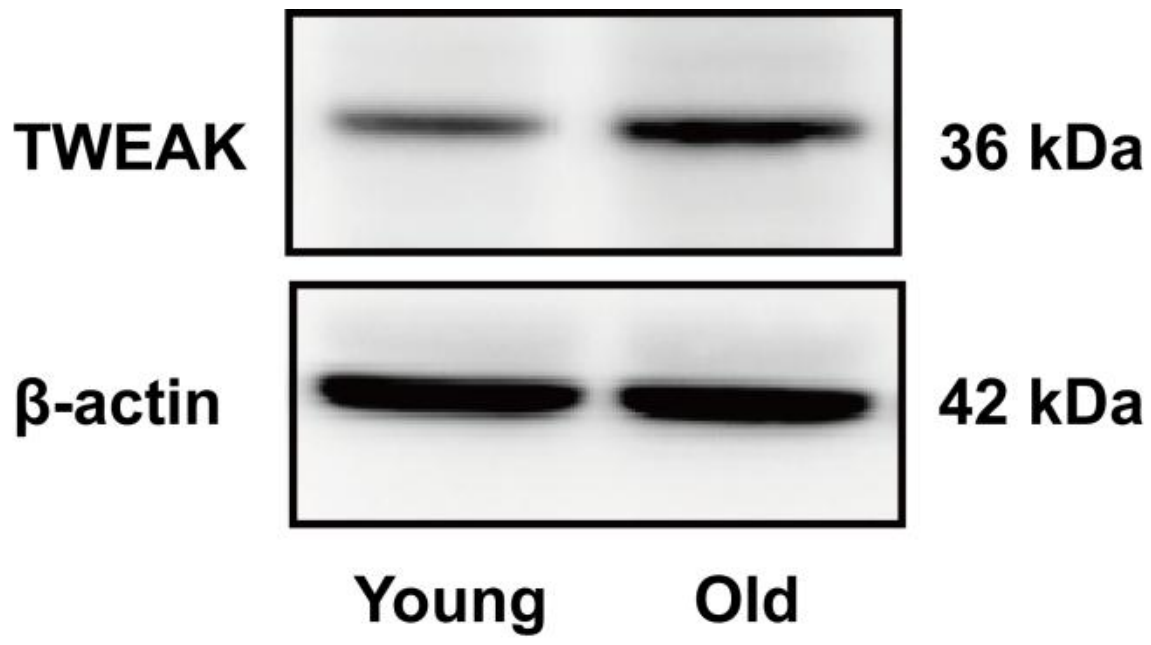

Figure 1F Raw Data

Panel A：TWEAK

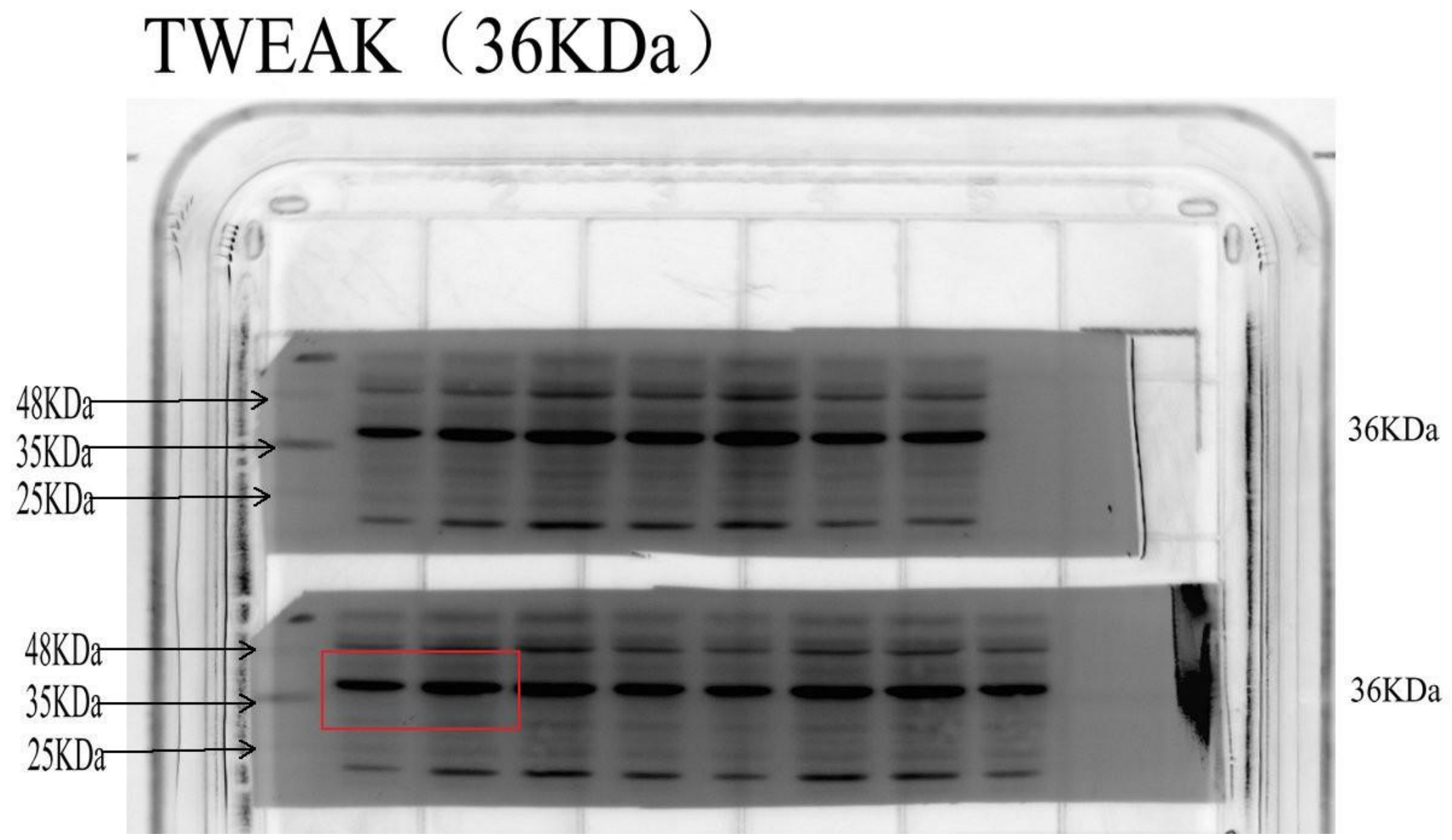

Details:

Protein: TWEAK

Predicted Size: 36 kDa

Marker Used: Solarbio Prestained Protein Marker

Cat#: PR1910

Note: The membrane was physically cut prior to antibody incubation. The lane loading order is as follows: Lane 1: Young group , Lane 2： Old group . This order matches the presentation in the manuscript.

Panel B：β-actin

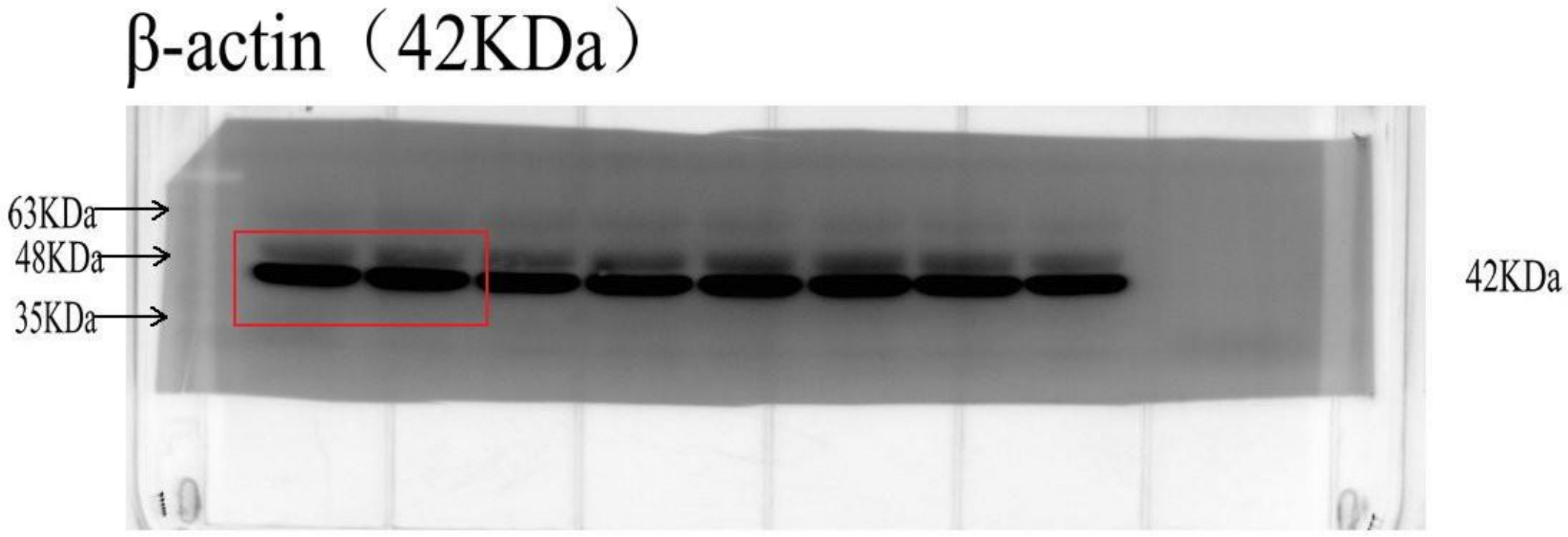

Details:

Protein: β-actin

Predicted Size: 42 kDa

Marker Used: Solarbio Prestained Protein Marker

Cat#: PR1910

Note: The membrane was physically cut prior to antibody incubation. The lane loading order is as follows: Lane 1: Young group , Lane 2： Old group . This order matches the presentation in the manuscript.

Figure 3H

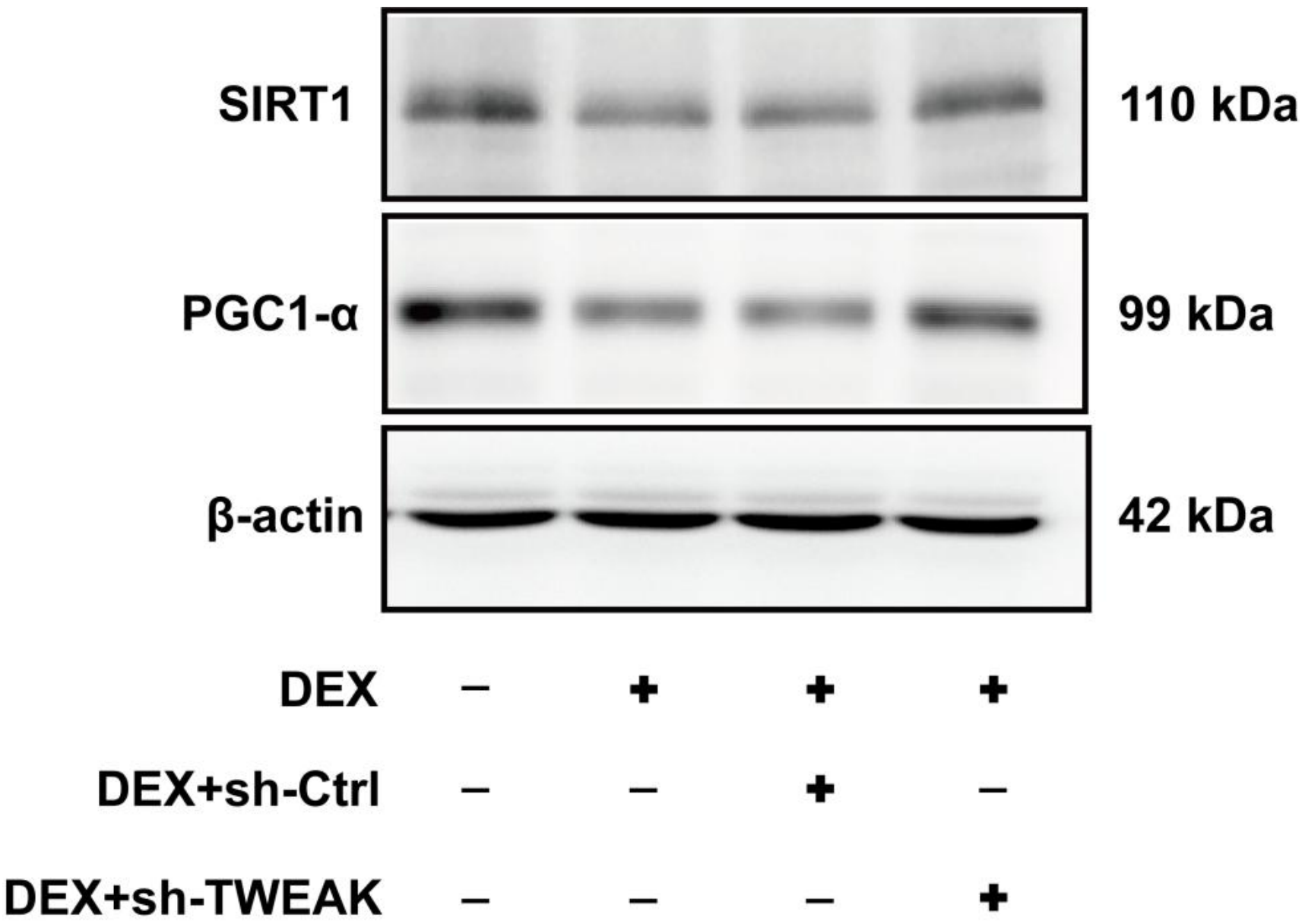
SIRT1
110 kDa
PGC1-α
99 kDa
β-actin
42 kDa
DEX – + + +
DEX+sh-Ctrl – – + –
DEX+sh-TWEAK – – – +

Figure 3H Raw Data

Panel A：SIRT1

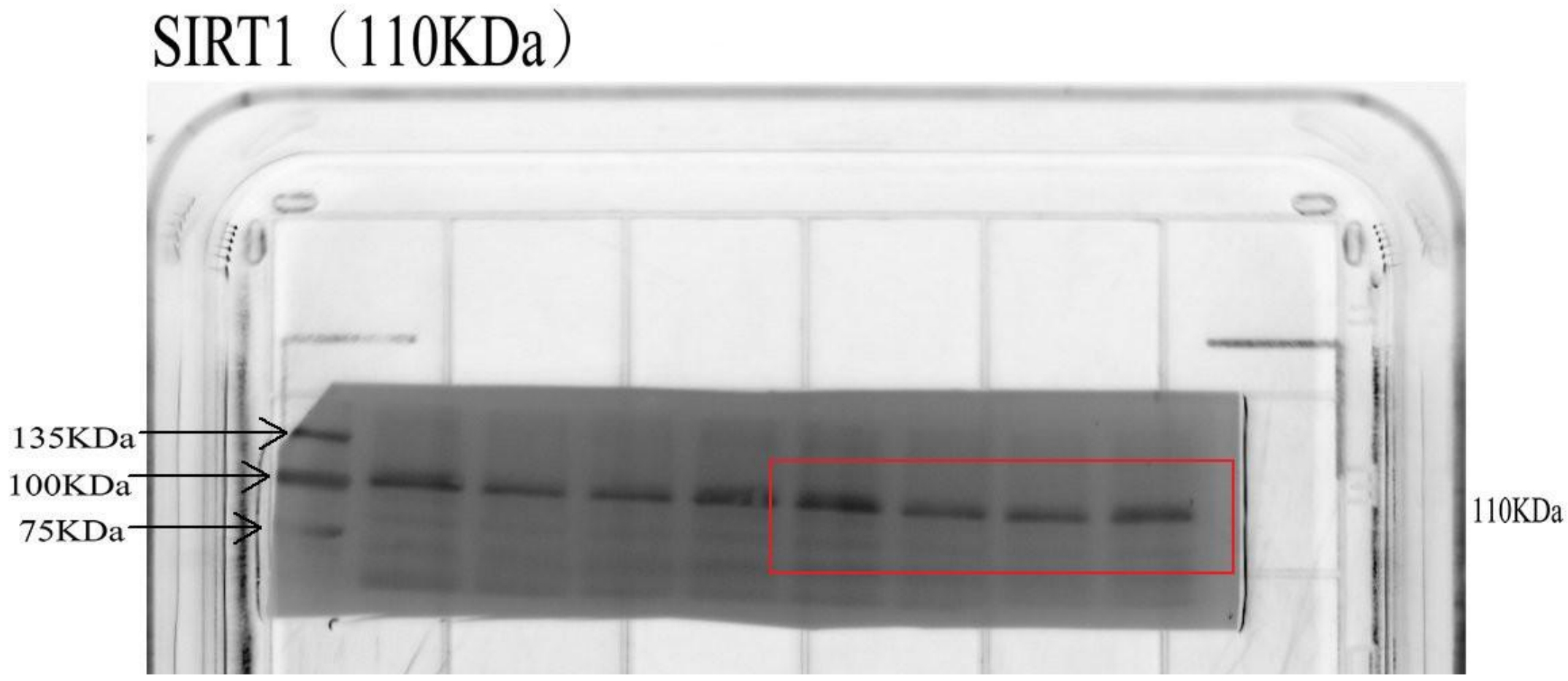

Details:

Protein: SIRT1

Predicted Size: 110 kDa

Marker Used: Solarbio Prestained Protein Marker

Cat#: PR1910

Note: The membrane was physically cut prior to antibody incubation. The lane loading order is as follows: Lane 5: Control group , Lane 6： DEX group, Lane 7：DEX+sh-Ctrl group , Lane 8：：DEX+sh-TWEAK group . This order matches the presentation in the manuscript.

Panel B：PGCα

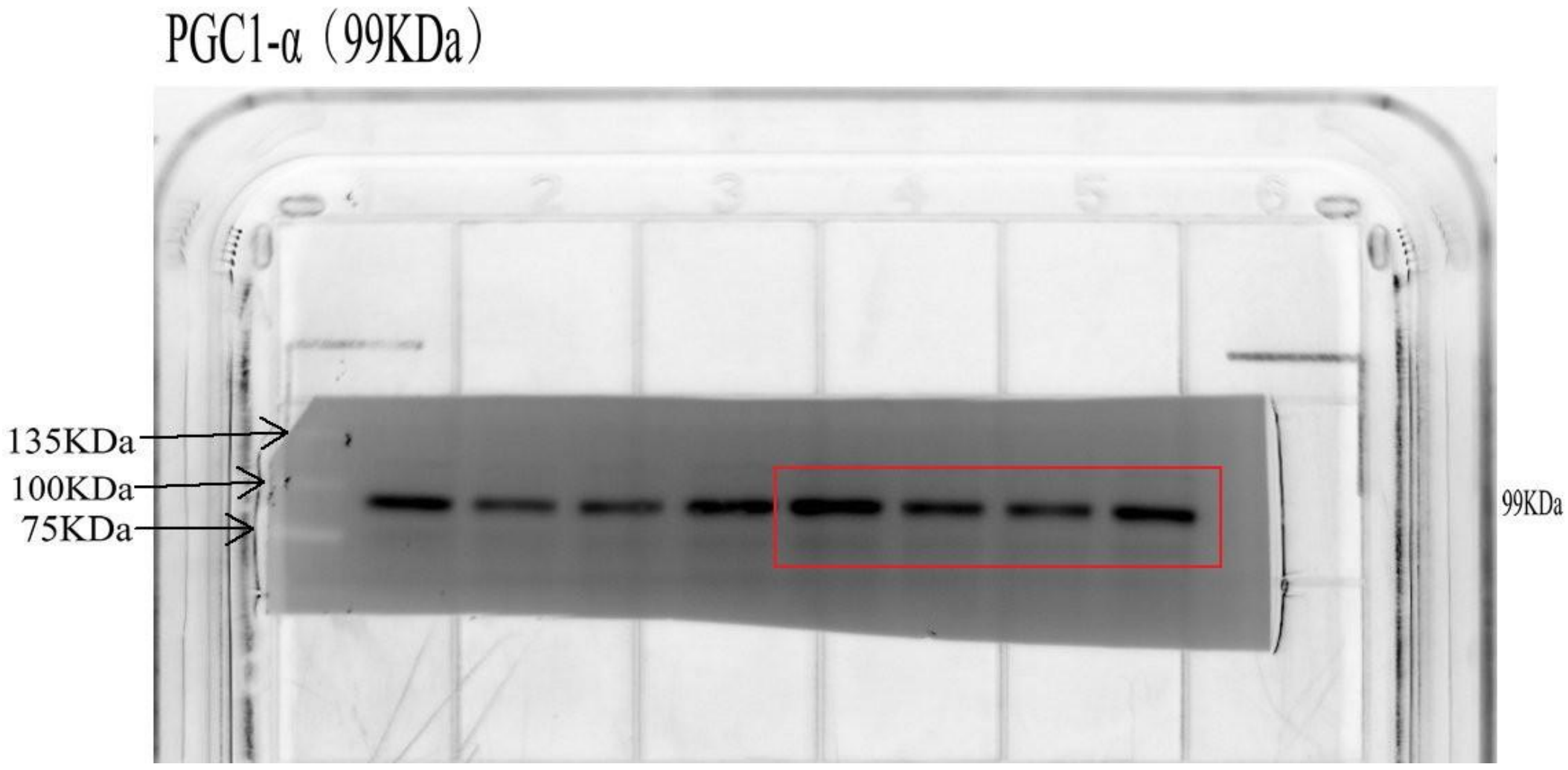

Details:

Protein: PGCα

Predicted Size: 99kDa

Marker Used: Solarbio Prestained Protein Marker

Cat#: PR1910

Note: The membrane was physically cut prior to antibody incubation. The lane loading order is as follows: Lane 5: Control group , Lane 6： DEX group, Lane 7：DEX+sh-Ctrl group , Lane 8：：DEX+sh-TWEAK group . This order matches the presentation in the manuscript.

Panel C：β-actin

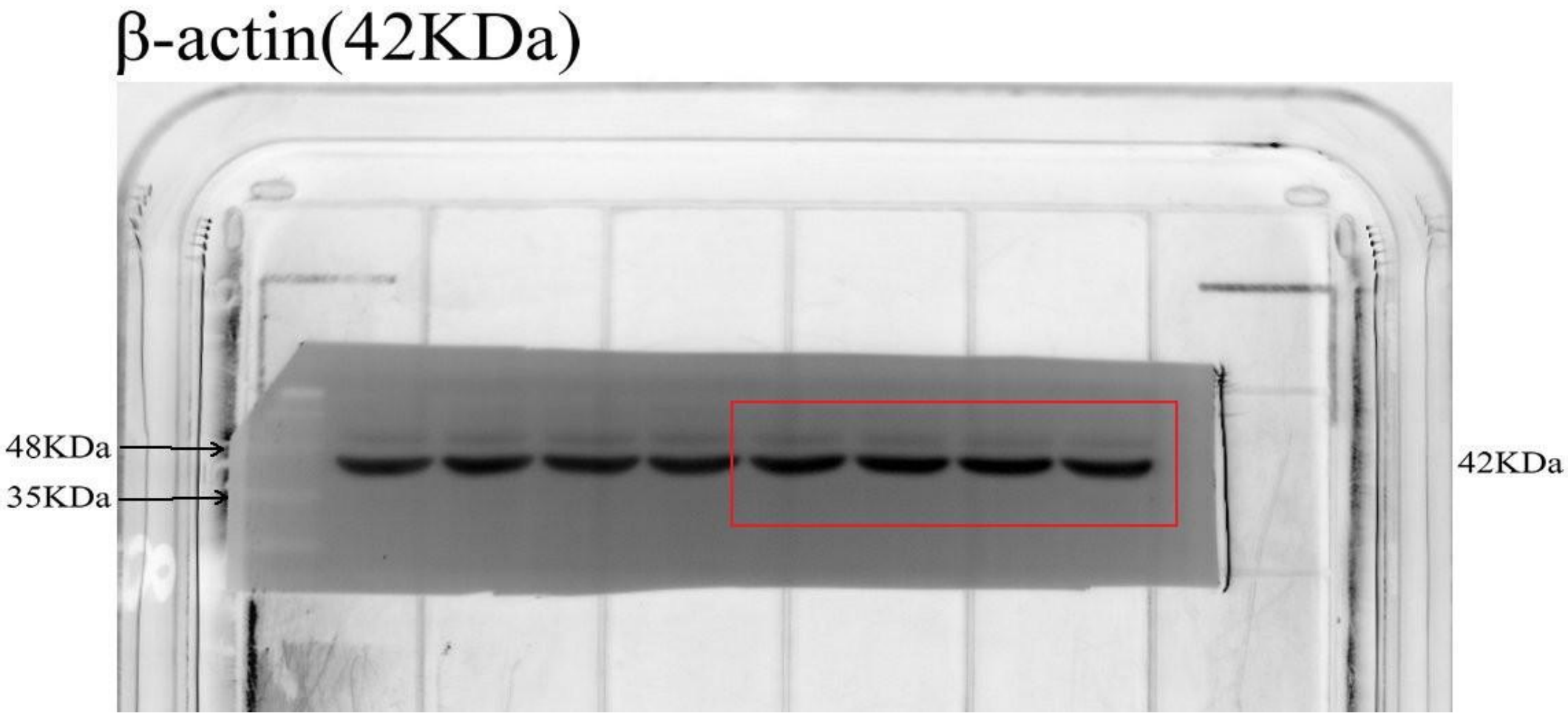

Details:

Protein: β-actin

Predicted Size: 42kDa

Marker Used: Solarbio Prestained Protein Marker

Cat#: PR1910

Note: The membrane was physically cut prior to antibody incubation. The lane loading order is as follows: Lane 5: Control group , Lane 6： DEX group, Lane 7：DEX+sh-Ctrl group , Lane 8：：DEX+sh-TWEAK group . This order matches the presentation in the manuscript.

Figure 4A

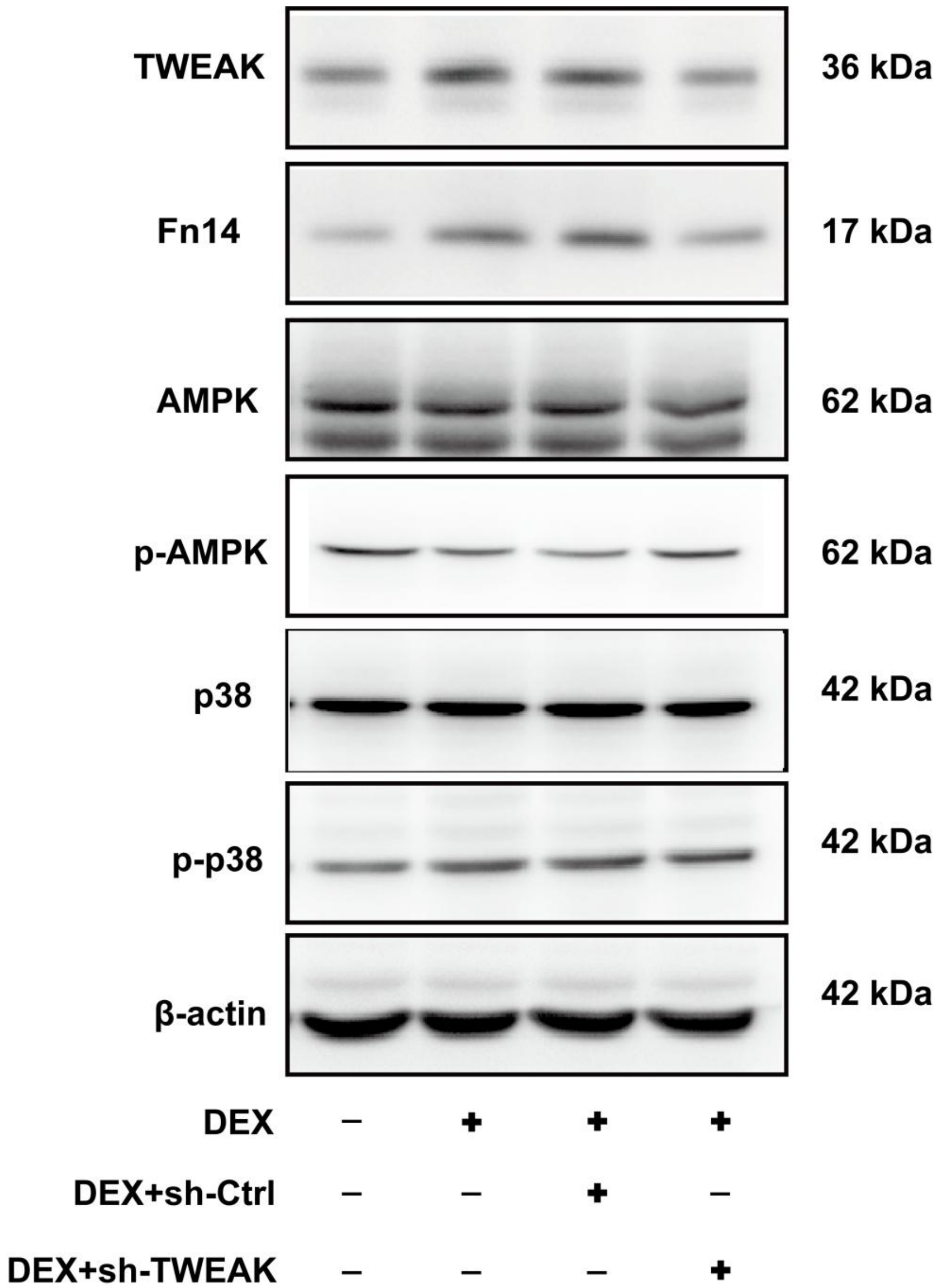
TWEAK
36 kDa
Fn14
17 kDa
AMPK
62 kDa
p-AMPK
62 kDa
p38
42 kDa
p-p38
42 kDa
β-actin
42 kDa
DEX – + + +
DEX+sh-Ctrl – – + –
DEX+sh-TWEAK – – – +

Figure 4A Raw Data

Panel A：TWEAK

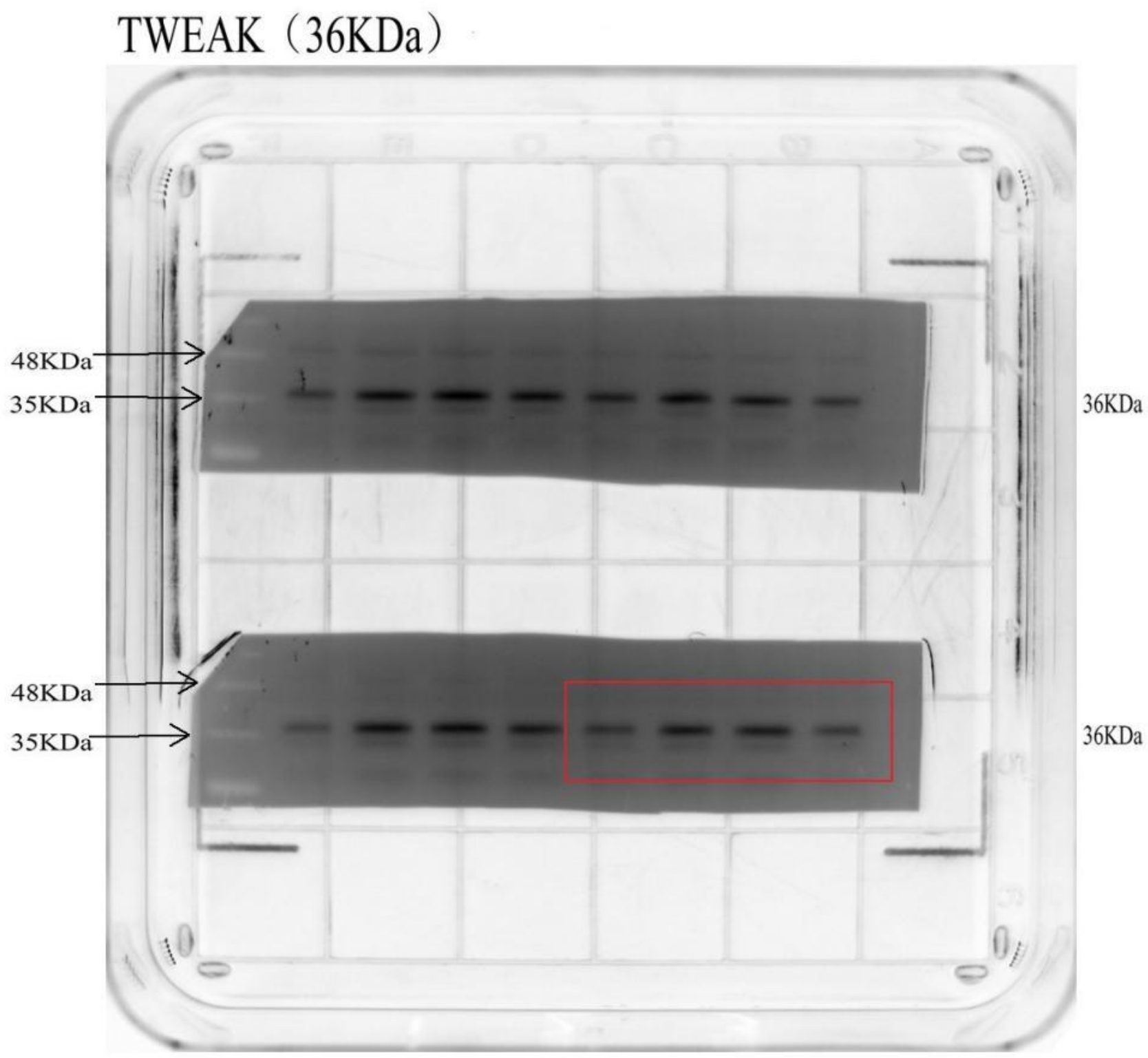

Details:

Protein: TWEAK

Predicted Size: 36kDa

Marker Used: Solarbio Prestained Protein Marker

Cat#: PR1910

Note: The membrane was physically cut prior to antibody incubation. The lane loading order is as follows: Lane 5: Control group , Lane 6： DEX group, Lane 7：DEX+sh-Ctrl group , Lane 8：：DEX+sh-TWEAK group . This order matches the presentation in the manuscript.

Panel B：Fn14

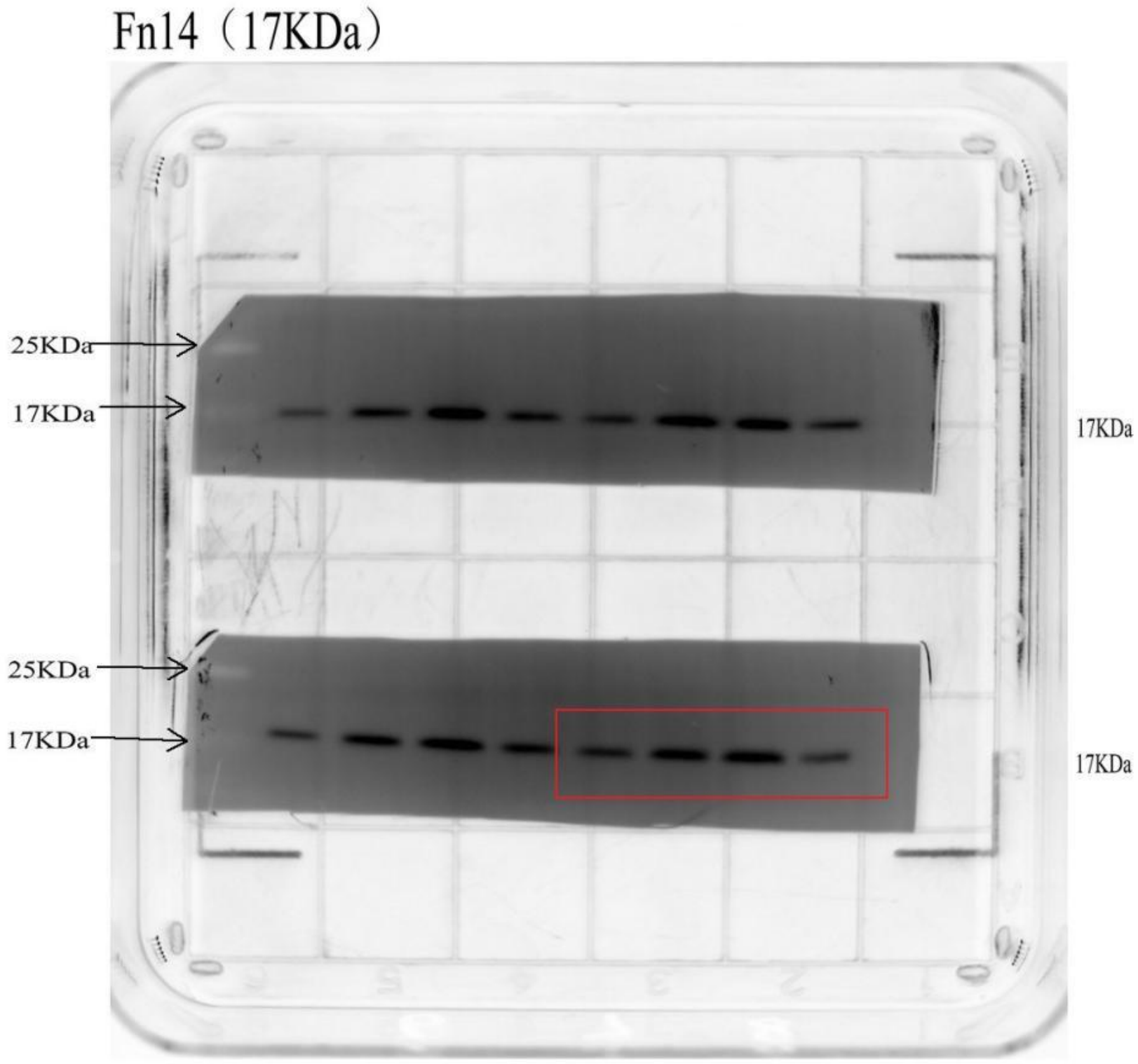

Details:

Protein: Fn14

Predicted Size: 17kDa

Marker Used: Solarbio Prestained Protein Marker

Cat#: PR1910

Note: The membrane was physically cut prior to antibody incubation. The lane loading order is as follows: Lane 5: Control group , Lane 6： DEX group, Lane 7：DEX+sh-Ctrl group , Lane 8：：DEX+sh-TWEAK group . This order matches the presentation in the manuscript.

Panel C： AMPK

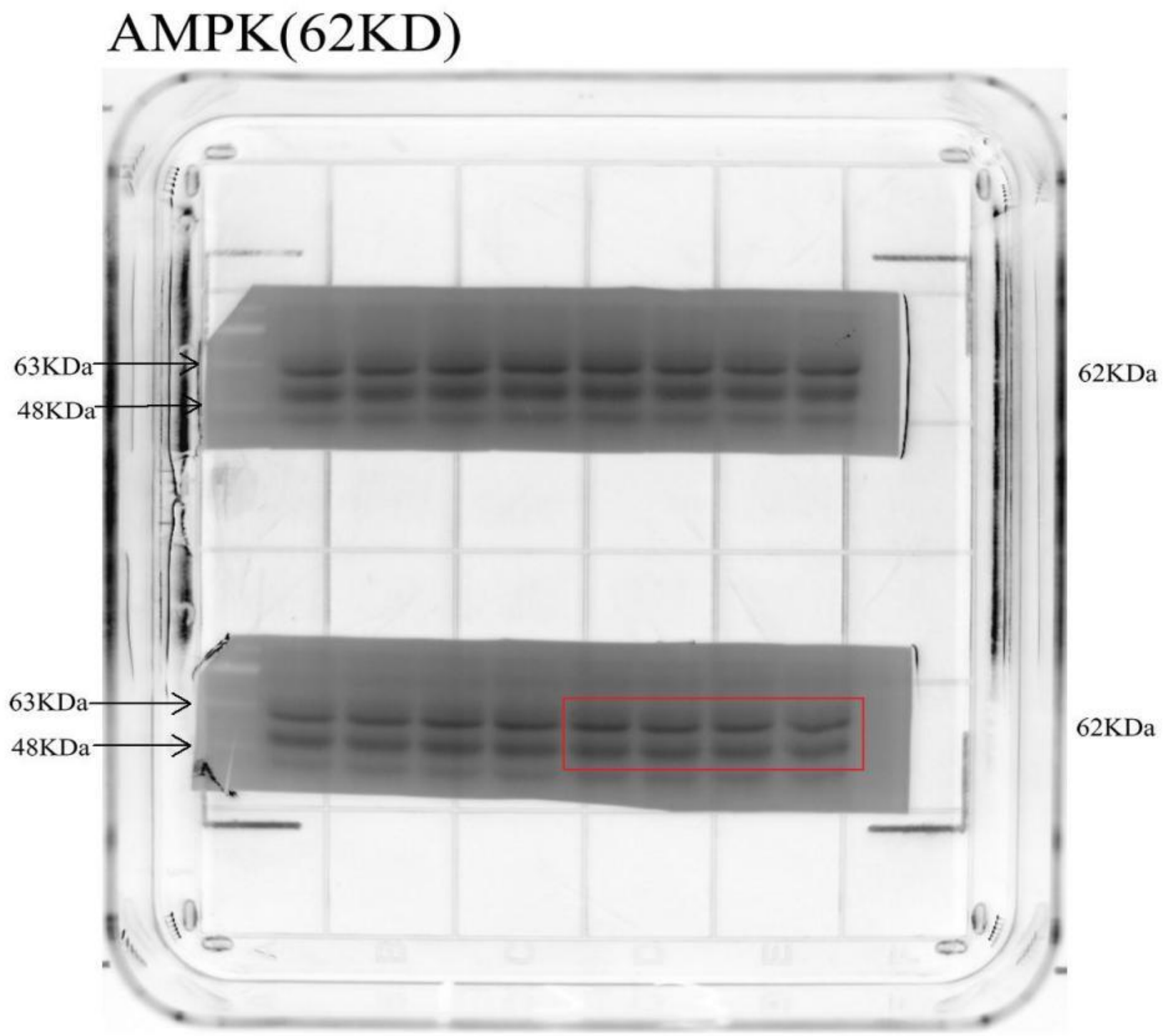

Details:

Protein: AMPK

Predicted Size: 62kDa

Marker Used: Solarbio Prestained Protein Marker

Cat#: PR1910

Note: The membrane was physically cut prior to antibody incubation. The lane loading order is as follows: Lane 5: Control group , Lane 6： DEX group, Lane 7：DEX+sh-Ctrl group , Lane 8：：DEX+sh-TWEAK group . This order matches the presentation in the manuscript.

Panel D：p-AMPK

p-AMPK（62KDa）

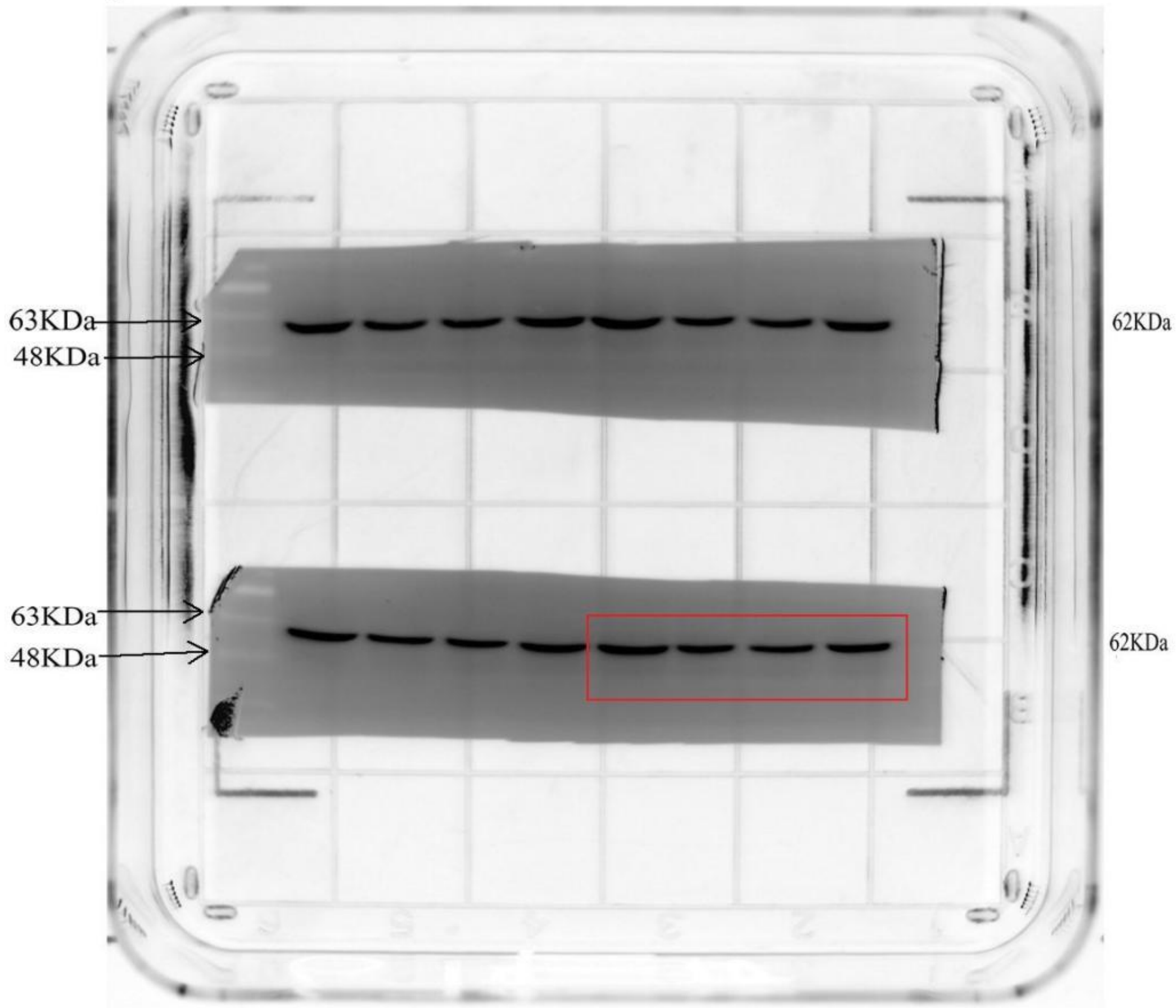

Details:

Protein: p-AMPK

Predicted Size:　62kDa

Marker Used: Solarbio Prestained Protein Marker

Cat#: PR1910

Note: The membrane was physically cut prior to antibody incubation. The lane loading order is as follows: Lane 5: Control group , Lane 6： DEX group, Lane 7：DEX+sh-Ctrl group , Lane 8：：DEX+sh-TWEAK group . This order matches the presentation in the manuscript.

Panel E：p38

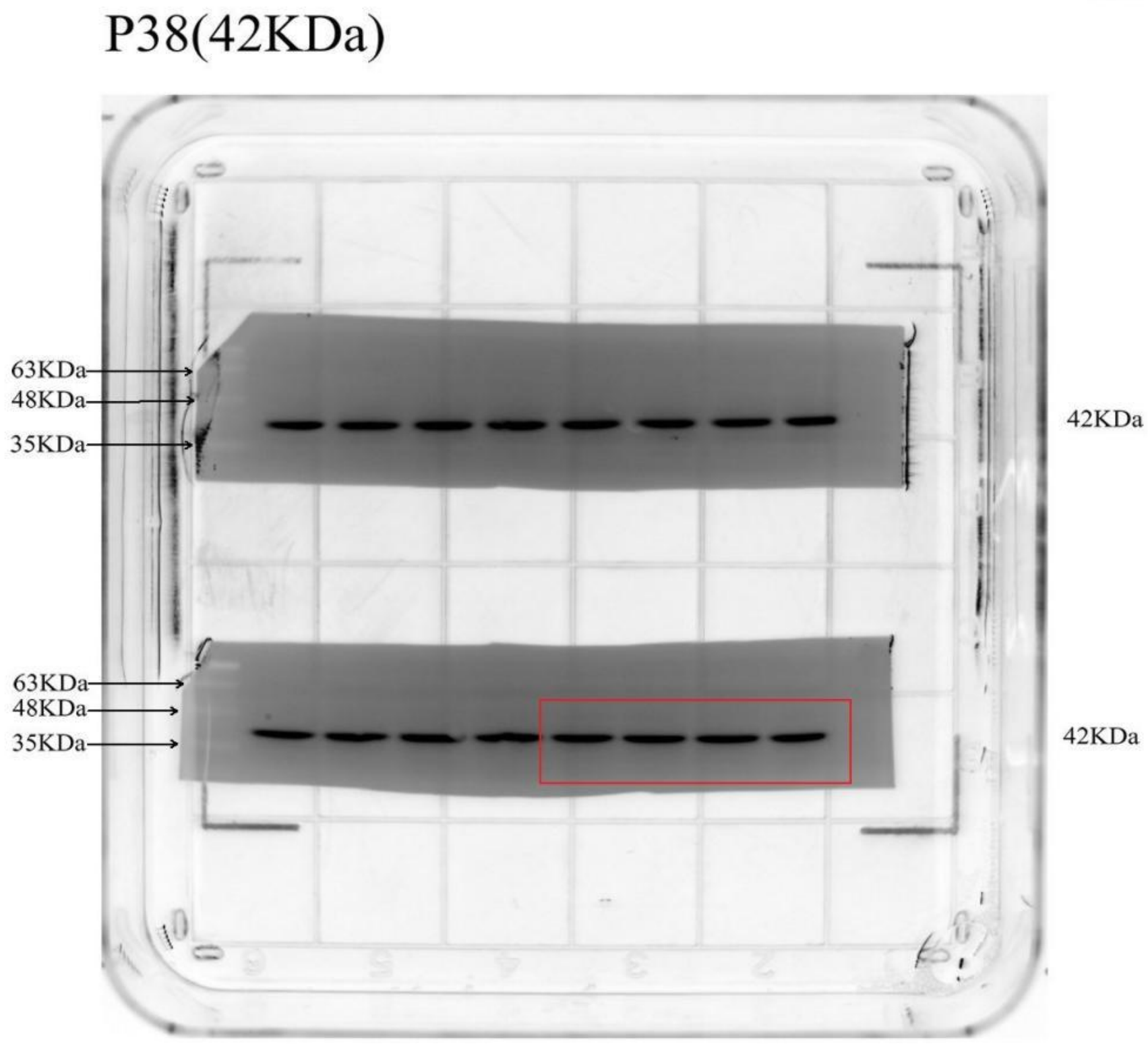

Details:

Protein: p38

Predicted Size: 42kDa

Marker Used: Solarbio Prestained Protein Marker

Cat#: PR1910

Note: The membrane was physically cut prior to antibody incubation. The lane loading order is as follows: Lane 5: Control group , Lane 6： DEX group, Lane 7：DEX+sh-Ctrl group , Lane 8：：DEX+sh-TWEAK group . This order matches the presentation in the manuscript.

Panel F：p-p38

p-p38（42KDa）

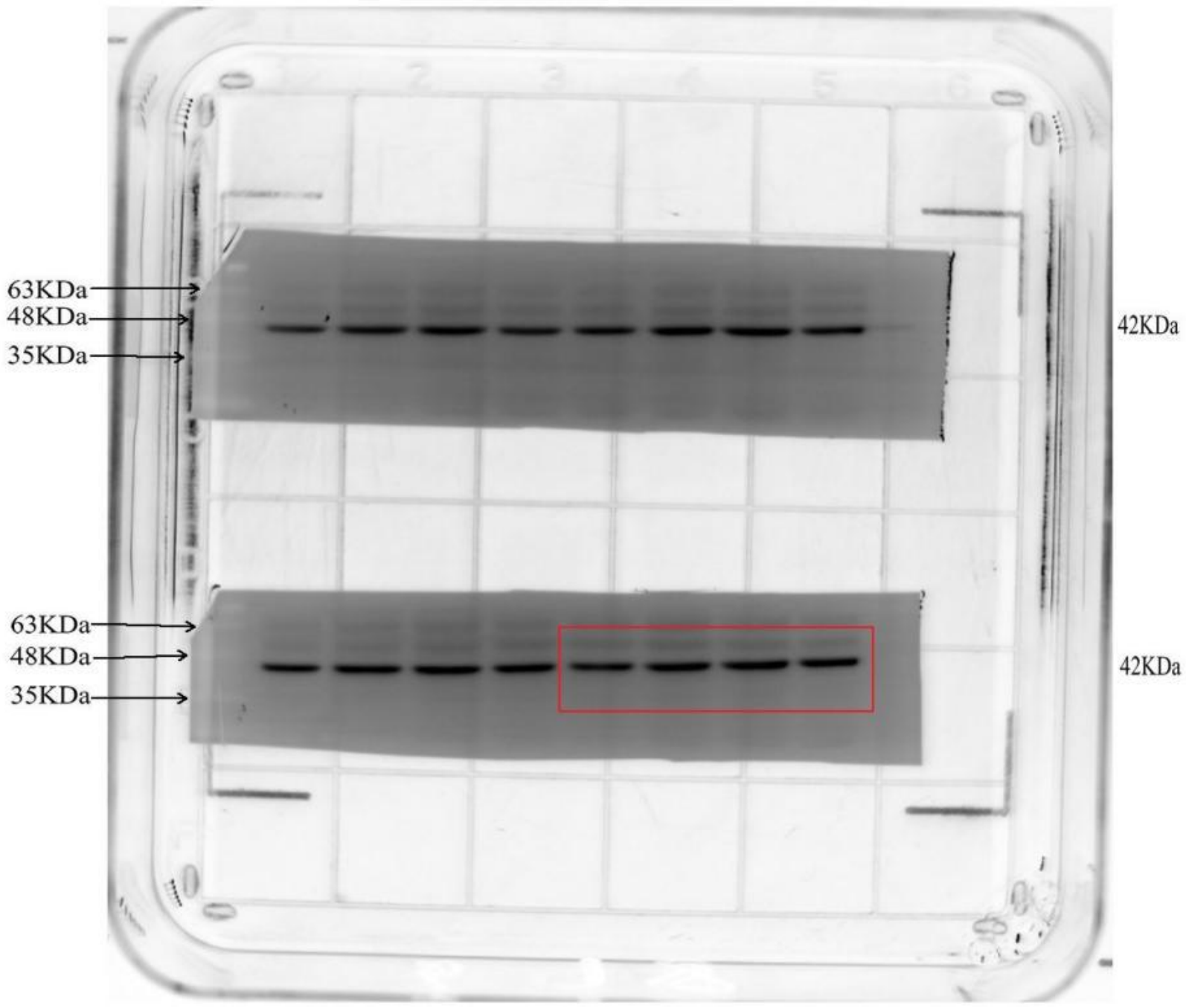

Details:

Protein: p-p38

Predicted Size: 42kDa

Marker Used: Solarbio Prestained Protein Marker

Cat#: PR1910

Note: The membrane was physically cut prior to antibody incubation. The lane loading order is as follows: Lane 5: Control group , Lane 6： DEX group, Lane 7：DEX+sh-Ctrl group , Lane 8：：DEX+sh-TWEAK group . This order matches the presentation in the manuscript.

Panel G：β-actin

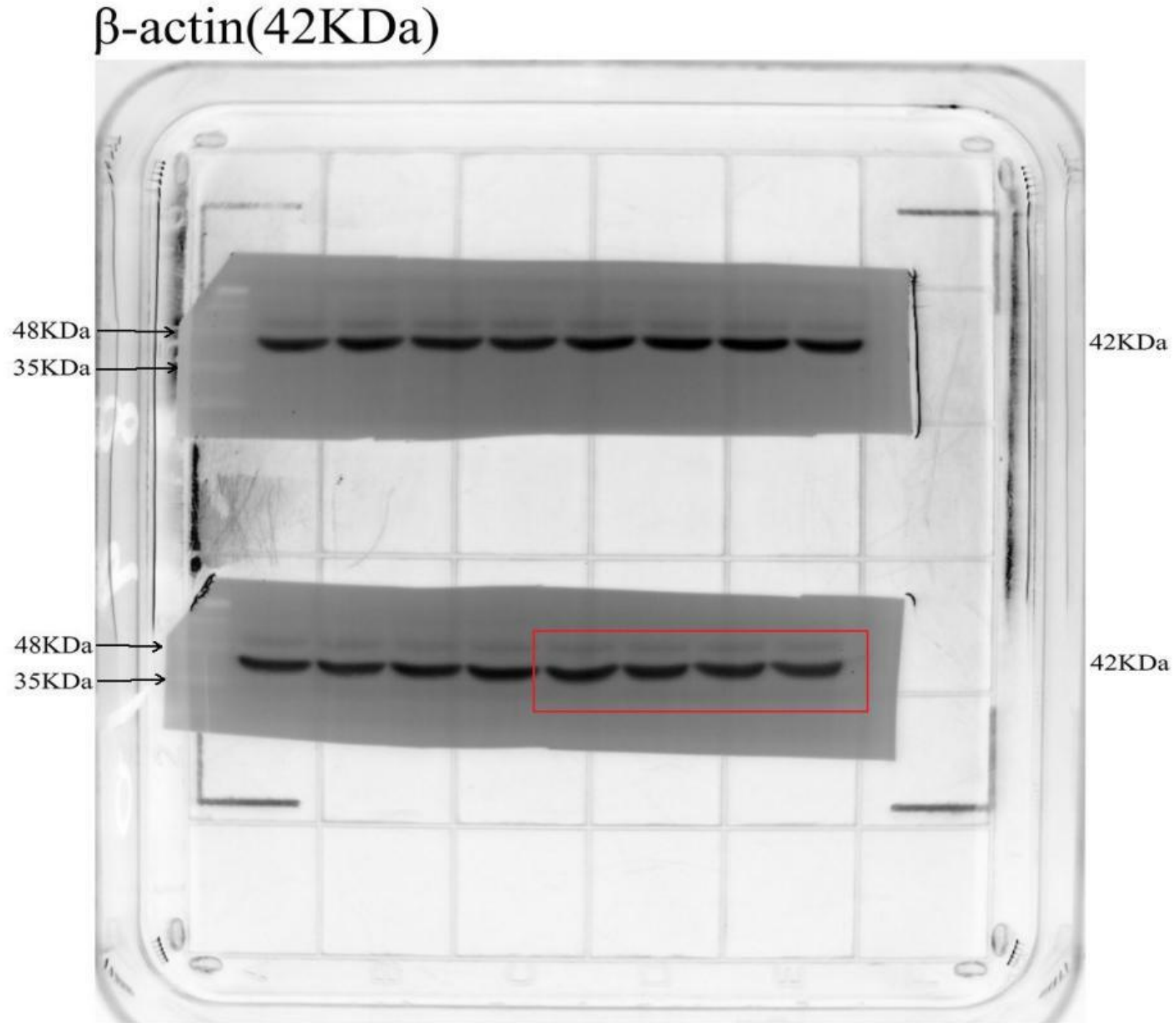

Details:

Protein: β-actin

Predicted Size: 42kDa

Marker Used: Solarbio Prestained Protein Marker

Cat#: PR1910

Note: The membrane was physically cut prior to antibody incubation. The lane loading order is as follows: Lane 5: Control group , Lane 6： DEX group, Lane 7：DEX+sh-Ctrl group , Lane 8：：DEX+sh-TWEAK group . This order matches the presentation in the manuscript.

Figure 6C

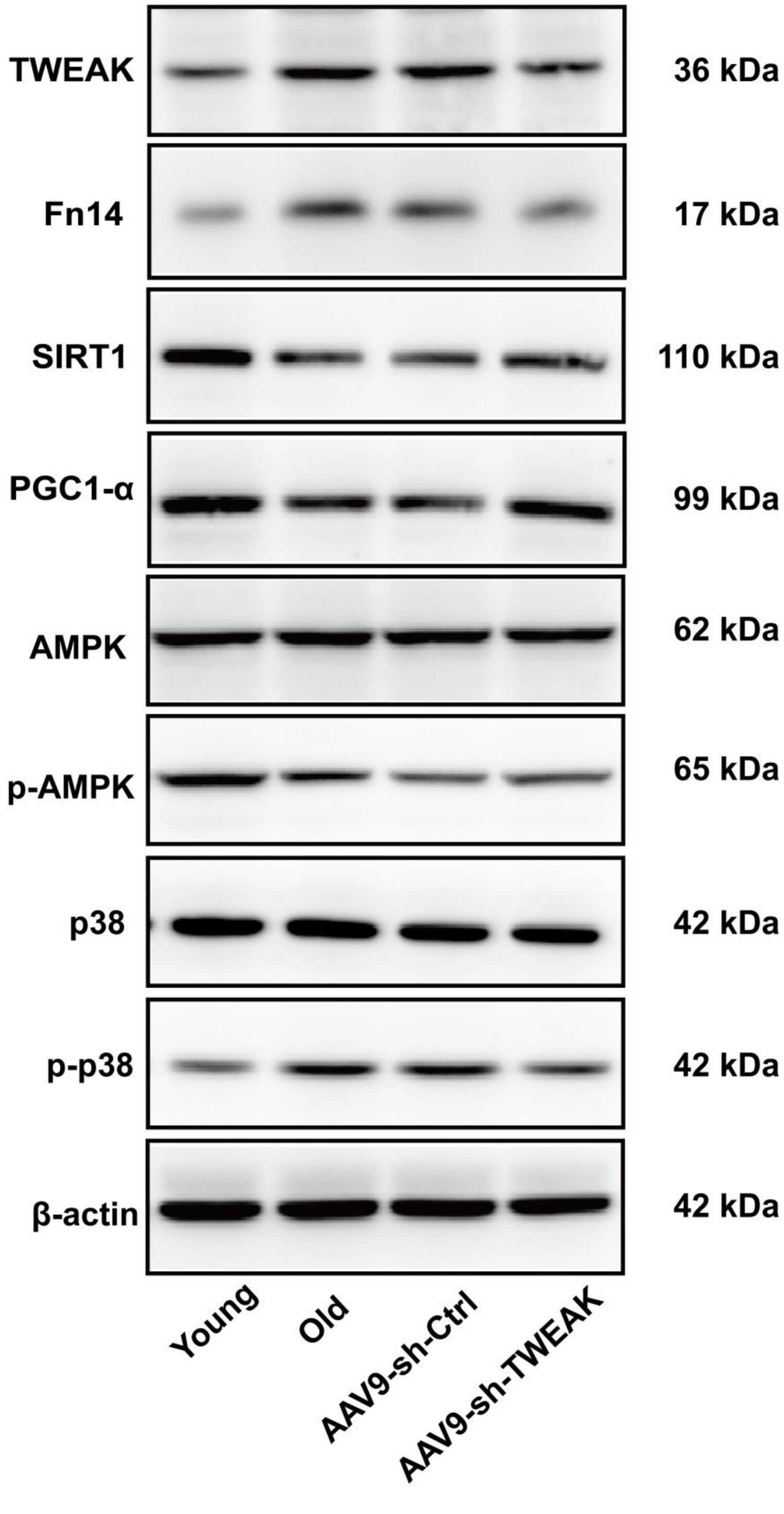

TWEAK
36 kDa
Fn14
17 kDa
SIRT1
110 kDa
PGC1-α
99 kDa
AMPK
62 kDa
p-AMPK
65 kDa
p38
42 kDa
p-p38
42 kDa
β-actin
42 kDa
Young
Old
AAV9-sh-Ctrl
AAV9-sh-TWEAK

Figure 6C Raw Data

Panel A：TWEAK

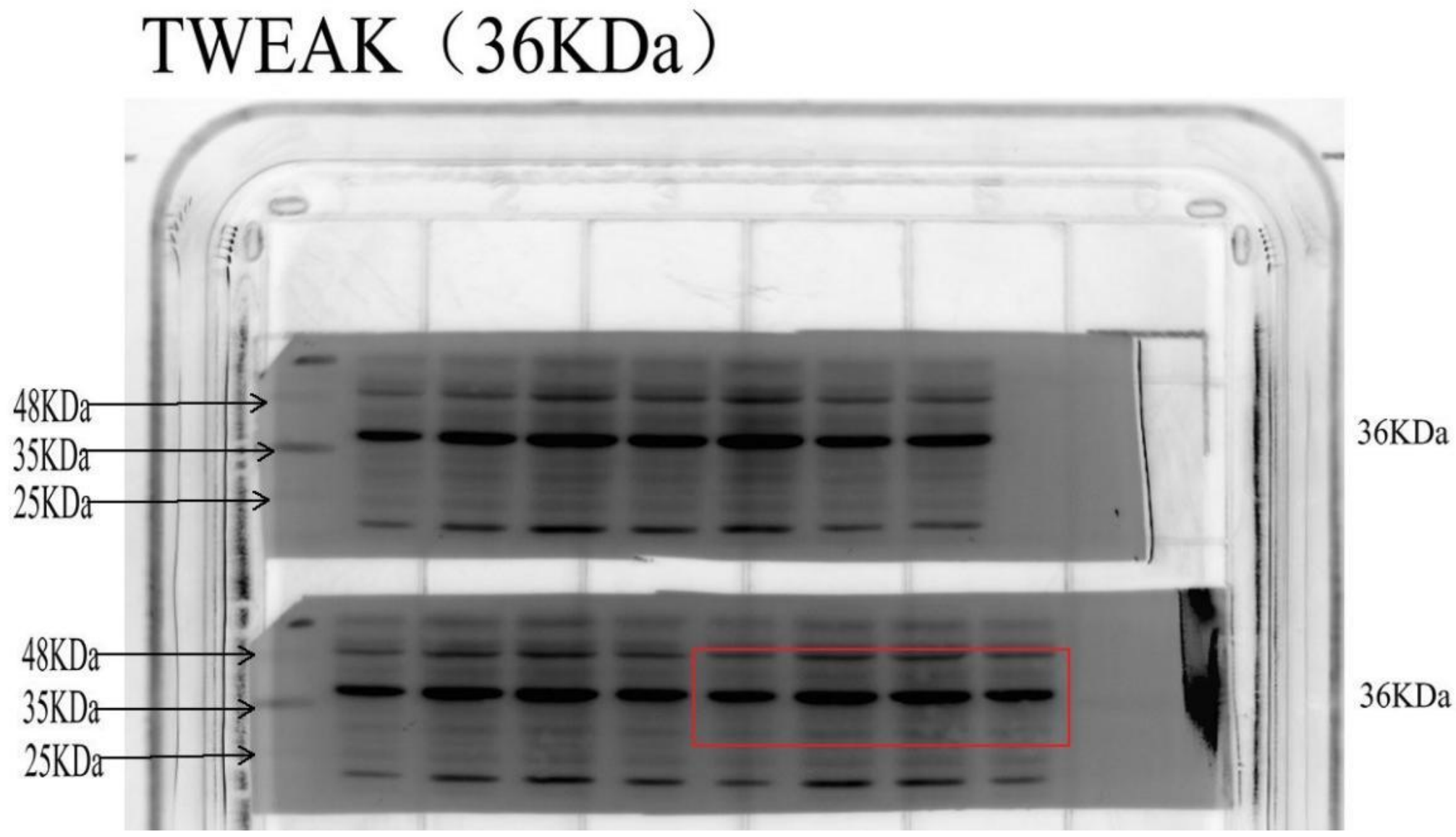

Details:

Protein: TWEAK

Predicted Size: 36kDa

Marker Used: Solarbio Prestained Protein Marker

Cat#: PR1910

Note: The membrane was physically cut prior to antibody incubation. The lane loading order is as follows: Lane 5: Young group , Lane 6：Old group, Lane 7： AAV9-sh-Ctrl group , Lane 8： AAV9-sh-TWEAK group. This order matches the presentation in the manuscript.

Panel B：Fn14

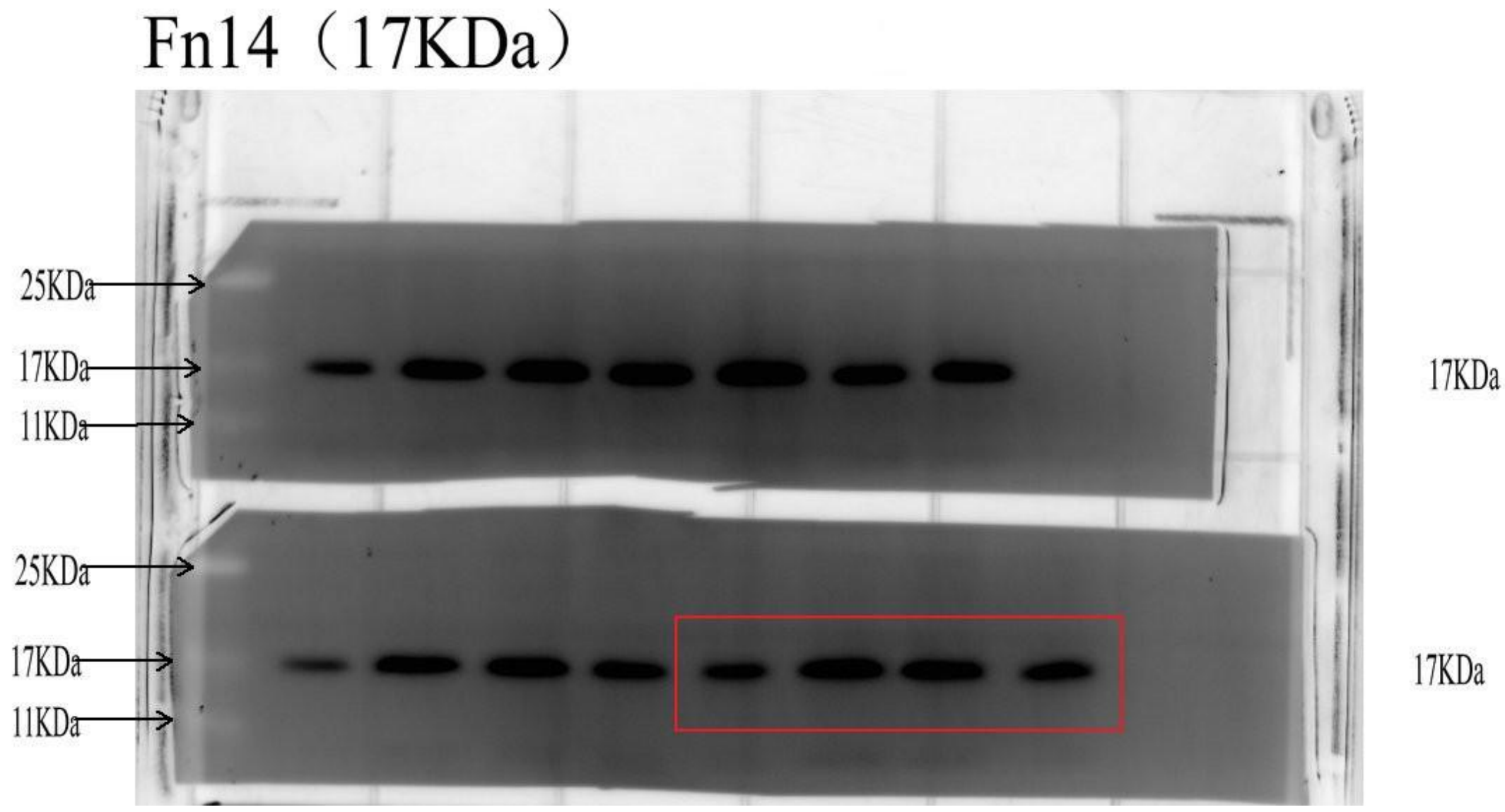

Details:

Protein: Fn14

Predicted Size: 17kDa

Marker Used: Solarbio Prestained Protein Marker

Cat#: PR1910

Note: The membrane was physically cut prior to antibody incubation. The lane loading order is as follows: Lane 5: Young group , Lane 6：Old group, Lane 7： AAV9-sh-Ctrl group , Lane 8： AAV9-sh-TWEAK group. This order matches the presentation in the manuscript.

Panel C：SIRT1

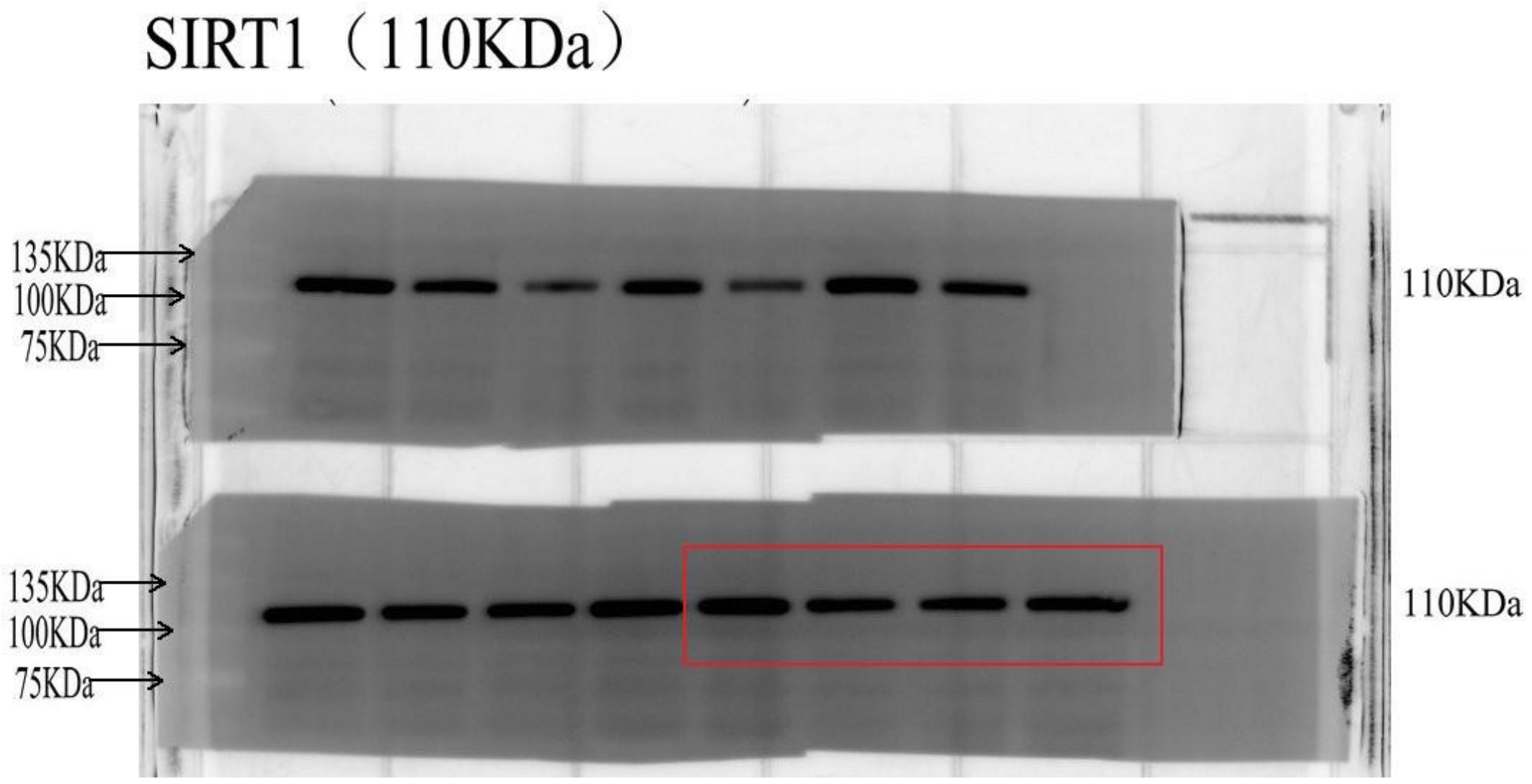

Details:

Protein: SIRT1

Predicted Size: 110kDa

Marker Used: Solarbio Prestained Protein Marker

Cat#: PR1910

Note: The membrane was physically cut prior to antibody incubation. The lane loading order is as follows: Lane 5: Young group , Lane 6：Old group, Lane 7： AAV9-sh-Ctrl group , Lane 8： AAV9-sh-TWEAK group. This order matches the presentation in the manuscript.

Panel D：PGC1-α

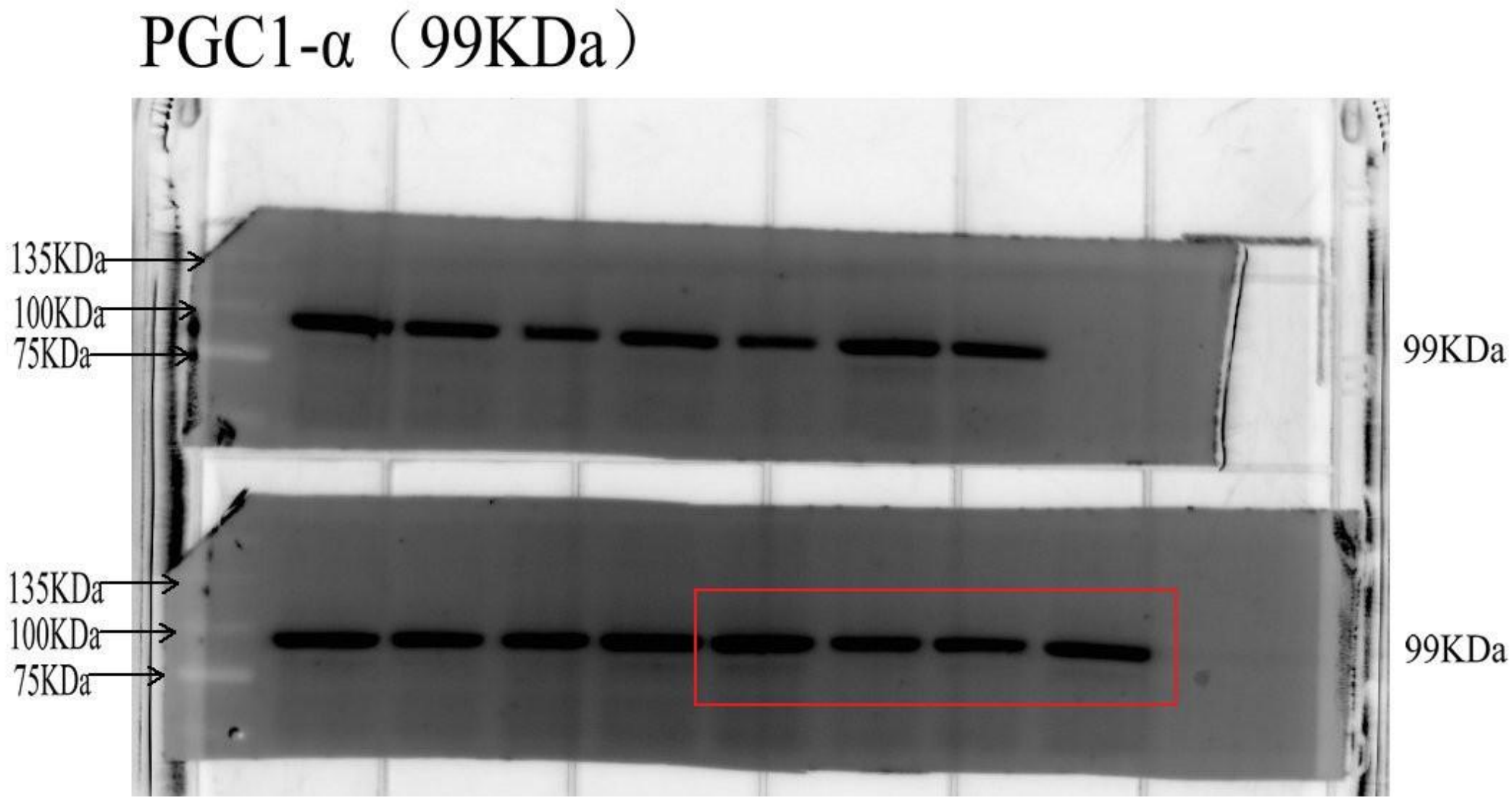

Details:

Protein: PGC1-α

Predicted Size: 99kDa

Marker Used: Solarbio Prestained Protein Marker

Cat#: PR1910

Note: The membrane was physically cut prior to antibody incubation. The lane loading order is as follows: Lane 5: Young group , Lane 6：Old group, Lane 7： AAV9-sh-Ctrl group , Lane 8： AAV9-sh-TWEAK group. This order matches the presentation in the manuscript.

Panel E：AMPK

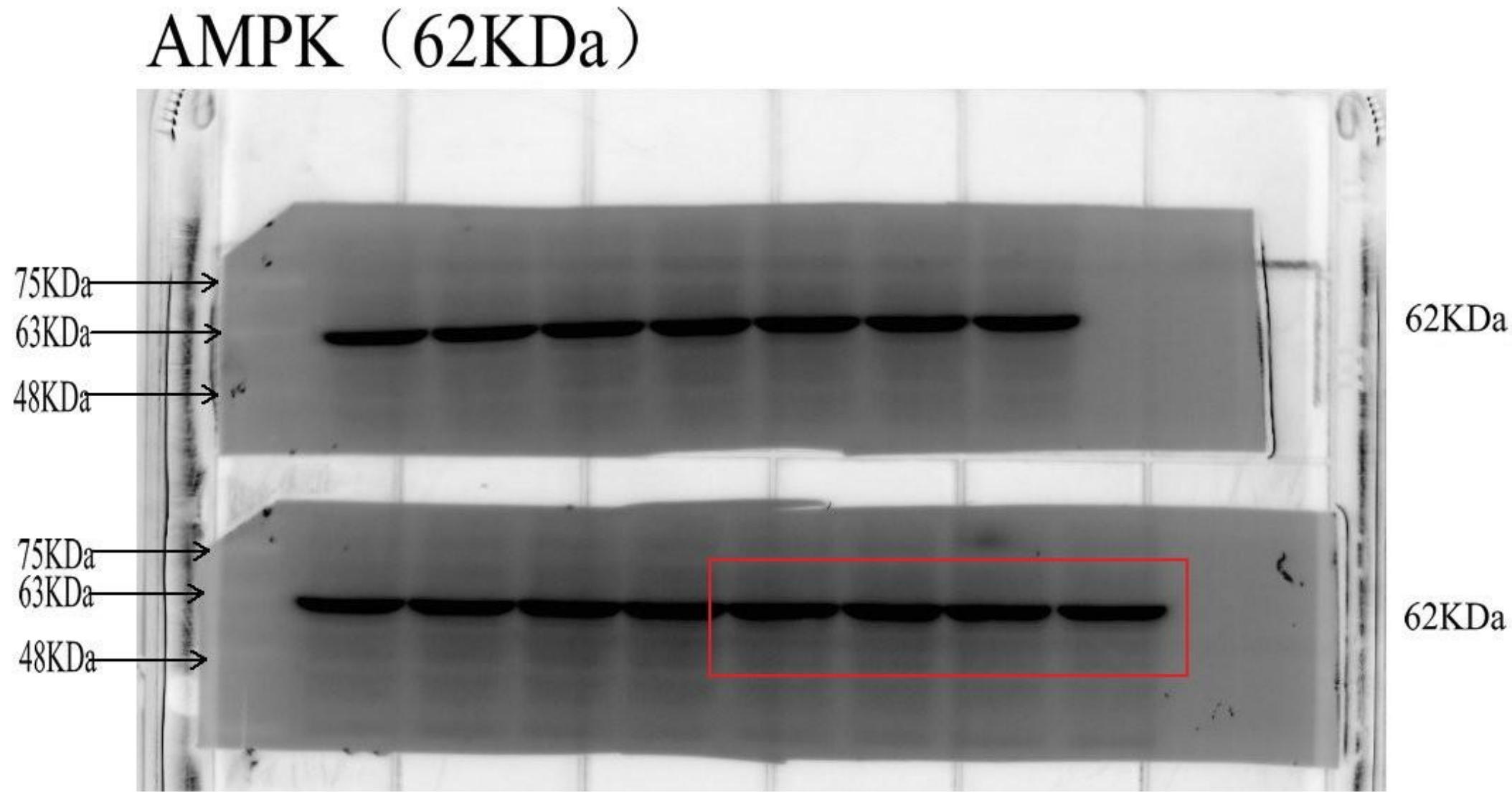

Details:

Protein: AMPK

Predicted Size: 62kDa

Marker Used: Solarbio Prestained Protein Marker

Cat#: PR1910

Note: The membrane was physically cut prior to antibody incubation. The lane loading order is as follows: Lane 5: Young group , Lane 6：Old group, Lane 7： AAV9-sh-Ctrl group , Lane 8： AAV9-sh-TWEAK group. This order matches the presentation in the manuscript.

Panel F：p-AMPK

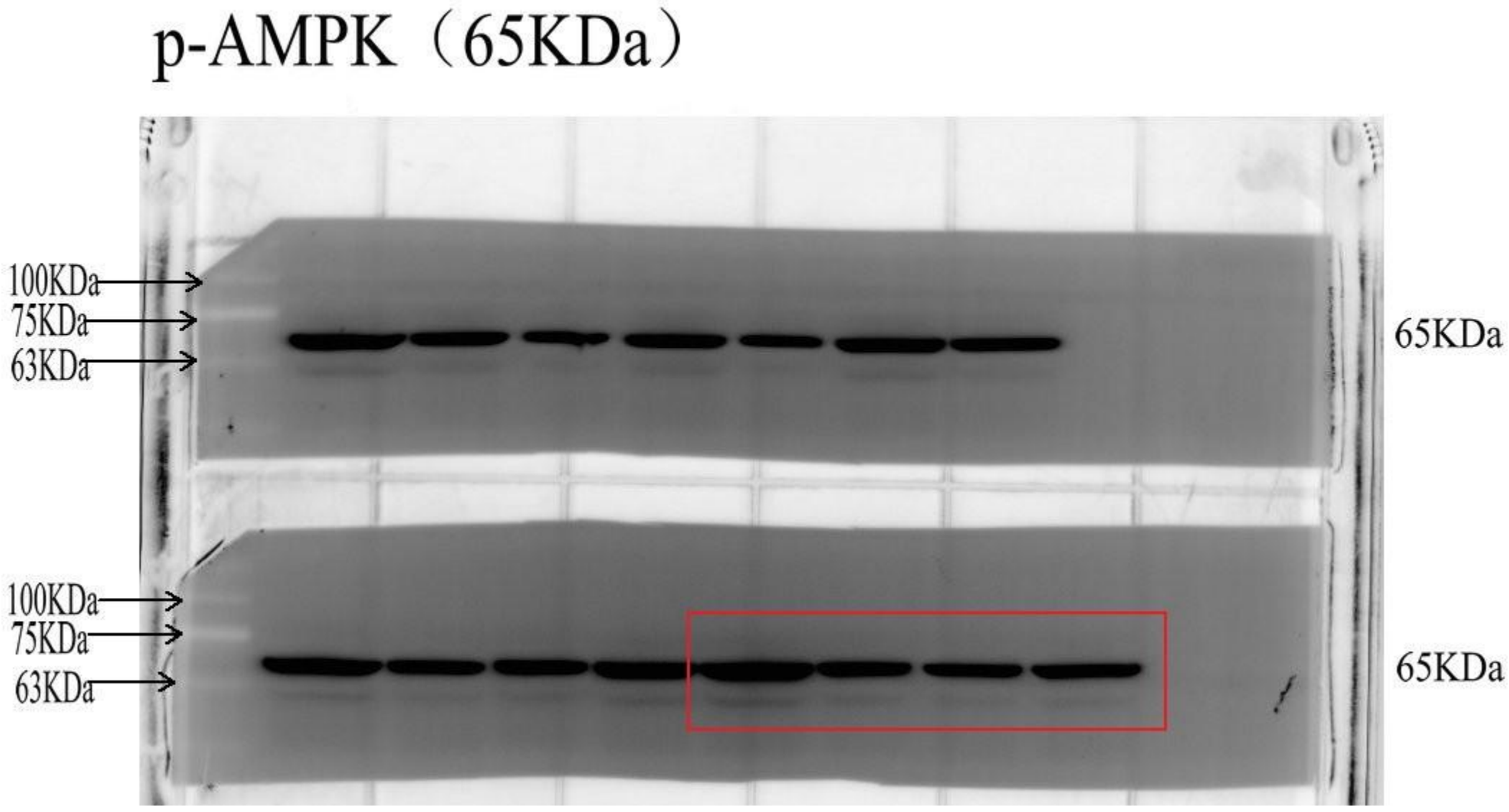

Details:

Protein: p-AMPK

Predicted Size: 65kDa

Marker Used: Solarbio Prestained Protein Marker

Cat#: PR1910

Note: The membrane was physically cut prior to antibody incubation. The lane loading order is as follows: Lane 5: Young group , Lane 6：Old group, Lane 7： AAV9-sh-Ctrl group , Lane 8： AAV9-sh-TWEAK group. This order matches the presentation in the manuscript.

Panel G：p38

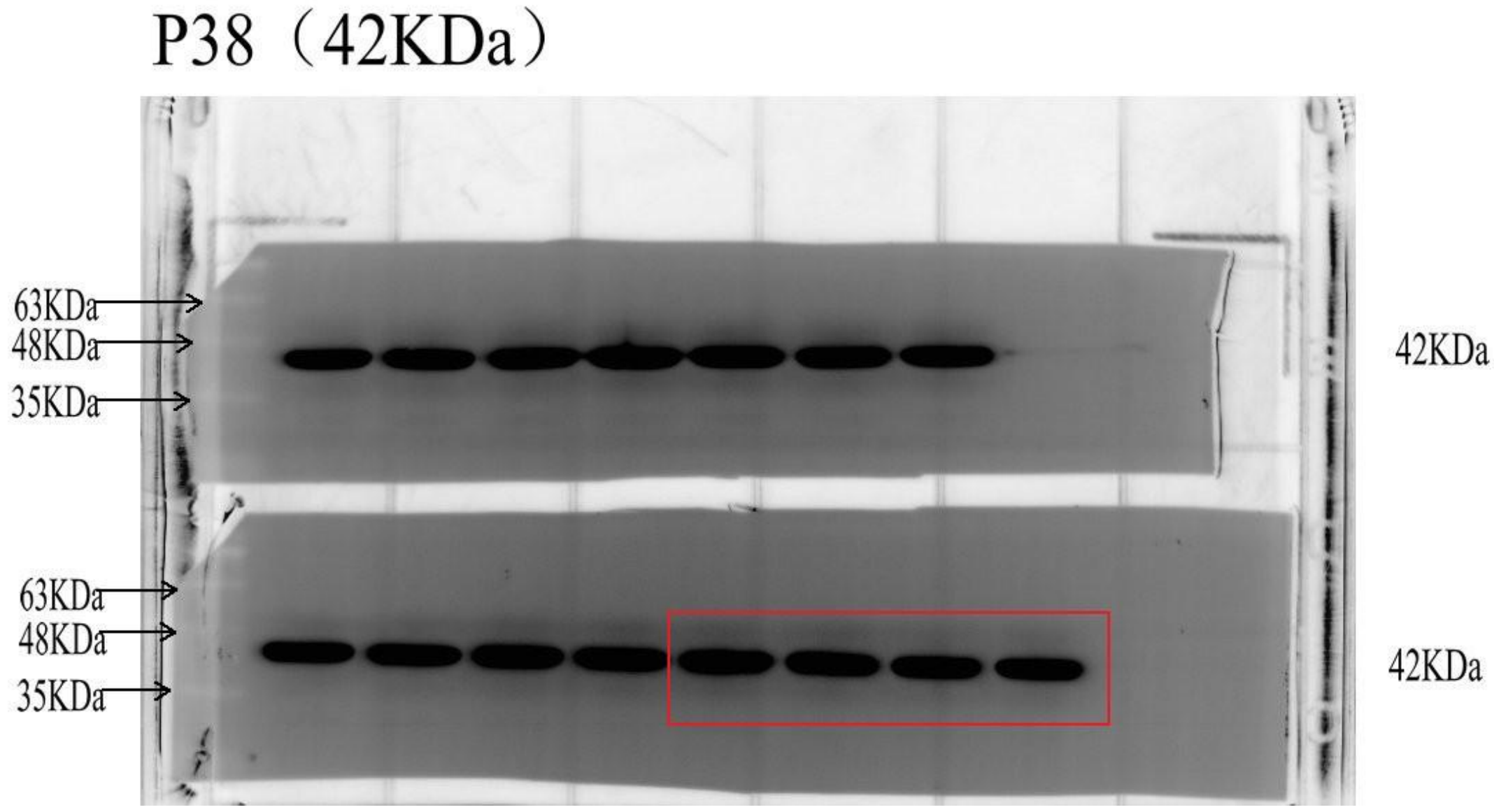

Details:

Protein: p38

Predicted Size: 42kDa

Marker Used: Solarbio Prestained Protein Marker

Cat#: PR1910

Note: The membrane was physically cut prior to antibody incubation. The lane loading order is as follows: Lane 5: Young group , Lane 6：Old group, Lane 7： AAV9-sh-Ctrl group , Lane 8： AAV9-sh-TWEAK group. This order matches the presentation in the manuscript.

Panel H：p-p38

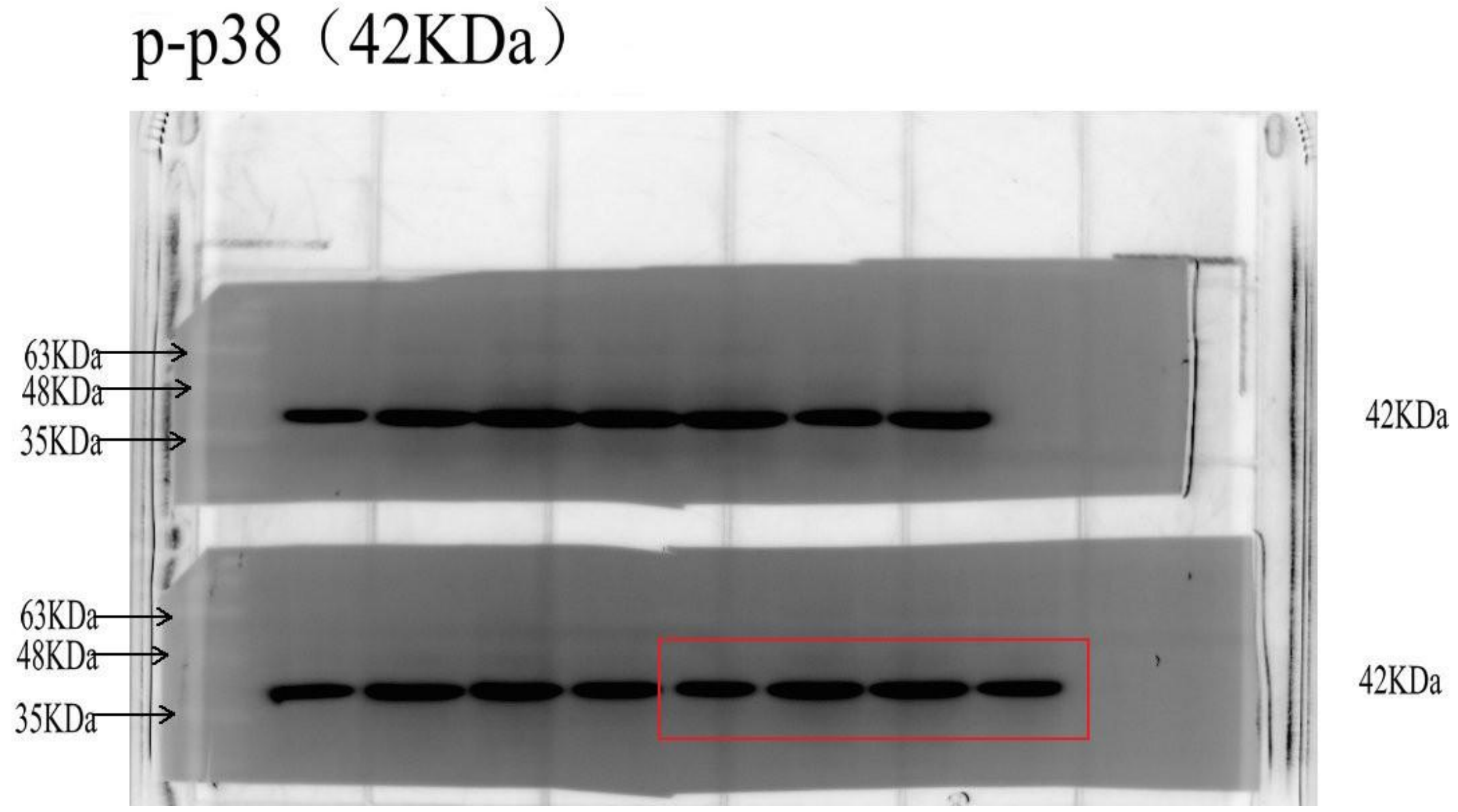

Details:

Protein: p-p38

Predicted Size: 42kDa

Marker Used: Solarbio Prestained Protein Marker

Cat#: PR1910

Note: The membrane was physically cut prior to antibody incubation. The lane loading order is as follows: Lane 5: Young group , Lane 6：Old group, Lane 7： AAV9-sh-Ctrl group , Lane 8： AAV9-sh-TWEAK group. This order matches the presentation in the manuscript.

Panel I：β-actin

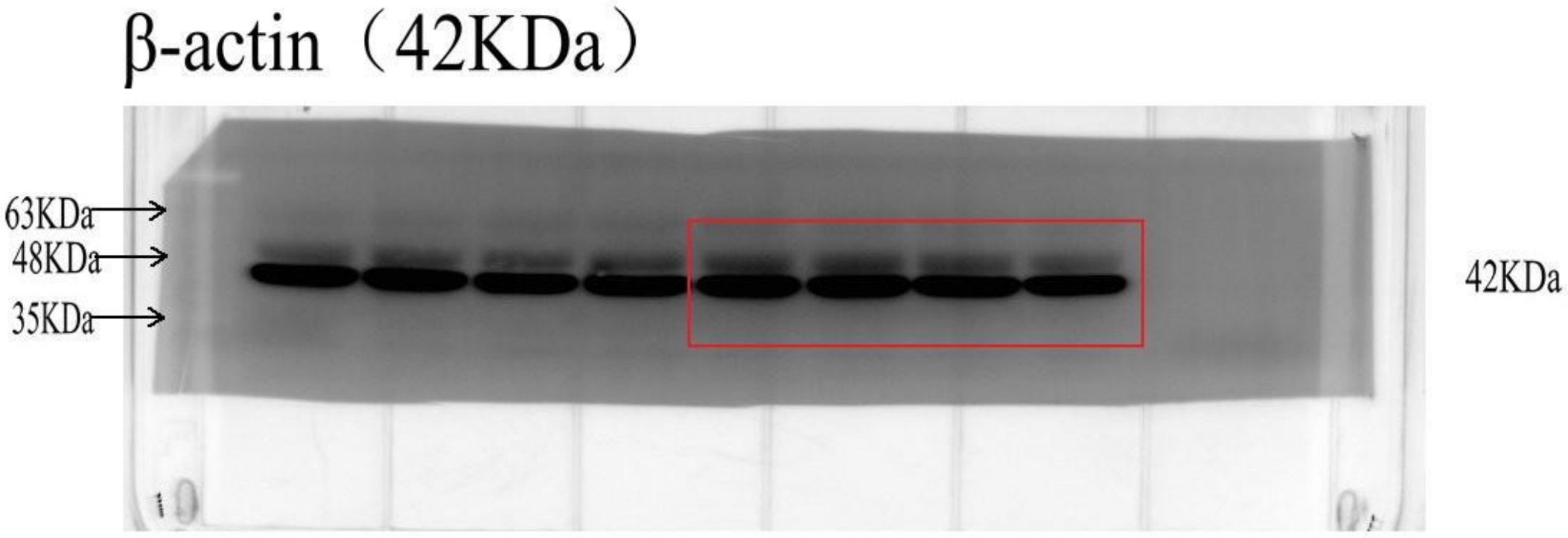

Details:

Protein: β-actin

Predicted Size: 42kDa

Marker Used: Solarbio Prestained Protein Marker

Cat#: PR1910

Note: The membrane was physically cut prior to antibody incubation. The lane loading order is as follows: Lane 5: Young group , Lane 6：Old group, Lane 7： AAV9-sh-Ctrl group , Lane 8： AAV9-sh-TWEAK group. This order matches the presentation in the manuscript.
